# Does the abiotic environment influence the distribution of flower and fruit colors?

**DOI:** 10.1002/ajb2.70044

**Published:** 2025-05-14

**Authors:** Agnes S. Dellinger, Leah Meier, Stacey Smith, Miranda Sinnott‐Armstrong

**Affiliations:** ^1^ Department of Botany and Biodiversity Research University of Vienna Rennweg 14 Vienna 1030 Austria; ^2^ Department of Ecology and Evolutionary Biology University of Colorado at Boulder 1800 Colorado Ave. Boulder 80309 USA CO

**Keywords:** abiotic factors, Bogert's rule, color diversity, flower color, fruit color, global distribution, Gloger's rule

## Abstract

**Premise:**

Color in flowers and fruits carries multiple functions, from attracting animal partners (pollinators, dispersers) to mitigating environmental stress (cold, drought, UV‐B). With research historically focusing on biotic interactions as selective agents, however, it remains unclear whether abiotic stressors impact flower and fruit colors across large spatial scales and shape their global distribution. Moreover, although flowers and fruits are developmentally linked and exposed to the same macroclimatic conditions, whether they have similar (correlated) responses to environmental stress remains unknown.

**Methods:**

Leveraging a data set of 2815 animal‐pollinated and animal‐dispersed species from 51 plant clades, we tested whether the diversity and distribution of flower and fruit colors (scored into eight categories) is shaped by temperature, aridity, and UV‐B irradiance.

**Results:**

Global diversity of flower and fruit colors was uncoupled, with flower color diversity generally lower than fruit color diversity and peaking in areas of high abiotic stress. Fruit color diversity peaked in tropical areas where the diversity of animal mutualists is highest. These distinct patterns were shaped by different responses of individual flower and fruit colors to abiotic stressors (for flowers, pink and red to cold temperatures, yellow and purple to UV‐B irradiance; for fruits, red to cold and wet conditions, black to warm, and yellow, green, and orange to UV‐B).

**Conclusions:**

Our results challenge the paradigm that flower and fruit colors are primarily shaped by animal partners but instead indicate that abiotic factors may set the macroecological stage for color evolution, with different selective factors acting on flowers and fruits.

Coloration in nature plays many roles, from attracting mates to camouflaging against a background to mediating environmental stress (Cuthill et al., 2017). These functions largely occur simultaneously, and selection on coloration must thus balance competing functions (Tenhumberg et al., 2023). Broadly, selective agents can be either biotic or abiotic, and the relative importance of biotic or abiotic selection presumably varies considerably (Verloop et al., 2020). In plants, the colors of flowers and fleshy fruits are primarily regarded as signals to attract mutualistic partners such as pollinators and dispersers (Valenta et al., 2018; Trunschke et al., 2021). The diversity of colors in both flowers and fruits is thus typically attributed to selection by animal mutualists, with distinct groups of pollinators and dispersers selecting for distinct colors (Schaefer et al., 2004; Lomáscolo and Schaefer, 2010; Valenta and Nevo, 2020; Trunschke et al., 2021). Other biotic selective agents have also been suggested, such as antagonistic interactors (i.e., herbivores, seed predators, pathogens), which may select for inconspicuousness. However, the contribution of antagonists as selective agents seems relatively minor in comparison to that of mutualists (Schaefer et al., 2004; Caruso et al., 2019; but see Schaefer, 2011; McCall et al., 2013).

In addition to these biotic drivers, abiotic factors are also important in shaping flower and fruit color variation by selecting on the pigments (type and quantity) used to produce flower and fruit colors. For example, across the ranges of several species, flowers (Lacey et al., 2010; Koski and Galloway, 2020) and fruits (Åkerström et al., 2010) have elevated pigment production and darker coloration at higher altitudes and/or latitudes, which may contribute to thermoregulation in colder environments (Bogert, 1949; Delhey, 2019). Both major classes of plant pigments (flavonoids and carotenoids) also have antioxidant capacities, and flavonoids in particular may mitigate stress from UV‐B exposure by acting as sunscreens, leading to increased production in areas of high UV‐B irradiance (Koski and Ashman, 2015; Peach et al., 2020; Li et al., 2021; Todesco et al., 2022). Moreover, flowers and fruits are highly susceptible to drought stress, which can be ameliorated by altering pigment production (Warren and Mackenzie, 2001; Vaidya et al., 2018; Espley and Jaakola, 2023; Burns, 2015).

While the protective function of pigments against abiotic stresses has been demonstrated repeatedly in plants, such associations have generally only been investigated in a small set of species or at the regional/intraspecific scale (Lev‐Yadun, 2015). These studies suggest that both flower and fruit colors vary non‐randomly across space (i.e., Vaidya et al., 2018; Sinnott‐Armstrong et al., 2021), but variation in methods and the variables tested makes it challenging to infer broad‐scale ecogeographic patterns. Links between coloration and the abiotic environment have a long history of study in animals, however. For animals, two major ecogeographic rules have been proposed: Bogert's rule and Gloger's rule. Bogert's rule predicts that ectothermic animals produce more darker pigments at higher latitudes or elevations to capture heat (Bogert, 1949; Delhey, 2019). In contrast, Gloger's rule predicts darker pigmentation (increased eumelanin) in warm and wet regions (near the equator), and more rufous coloration (increased phaeomelanin) in dry and warm regions (Gloger, 1833; Delhey, 2019). Although these two ecogeographic rules seemingly contradict each other, a recent assessment in birds has found both rules to apply and explain heterogeneity in climatic effects on plumage coloration (Delhey et al., 2019).

A similar assessment for plants—investigating how different abiotic climatic factors shape flower and fruit coloration at a global scale—is currently lacking. Furthermore, it is unclear whether to expect flower and fruit colors to follow the same or different ecogeographic rules. From a developmental perspective, flowers and fruits arise from the same set of organs and carry the same genetic toolkit for producing colorful pigments, which would predict similar color variation in both organs. On the other hand, flowers and fruits are often produced at different times of year and hence exposed to different climatic conditions and light environments (spring/fall; rainy/dry season; Valenta and Nevo, 2020). Given the well‐documented environmental associations for other plant functional traits (i.e., specific leaf area, Dwyer et al., 2014; growth form, Taylor et al., 2023; height, Moles et al., 2009; seed mass, Moles et al., 2007; wood density, Chave et al., 2009), we need to establish whether the global distribution of flower and fruit colors is also shaped by the abiotic environment.

If the abiotic environment is an important driver of the distribution of flower and fruit colors, we may expect major differences in the proportions of distinct flower and fruit colors across environments. Such differences can become apparent when comparing patterns of flower and fruit color diversity across regions and when testing whether distinct colors are associated with distinct environmental variables. Generally, flower and fruit colors do not seem to be represented evenly in communities and ecosystems (Kevan et al., 1996, Sinnott‐Armstrong et al., 2018; Delmas et al., 2020). In flowers, for example, white is the dominant (>75%) color across the wet tropics of Australia, while other colors (i.e., pink, red, green, yellow) are much less common. Similarly, in fruits, red and black fruits often make up more than 50% of the regional fruit color pool in the Australian wet tropics, while other colors (i.e., purple, blue, green) are less common (Delmas et al., 2020). Despite this general unevenness in color composition within communities, both the diversity of colors and their prevalence often shift widely across communities and broader scales (McEwen and Vamosi, 2010; Dalrymple et al., 2020; Delmas et al., 2020; Sinnott‐Armstrong et al., 2021), pointing to spatially varying factors shaping color variation (Narbona et al., 2024). For example, species in a community may shift away from a dominant color if this color is less successful at attracting mutualists (i.e., when outcompeted by co‐flowering plants of the same color, McEwen and Vamosi, 2010; Muchhala et al., 2014) or more susceptible to environmental stress (i.e., damaged by environmental stress; Ida and Totland, 2014). Thus, changes in color composition and diversity across large ecogeographic scales may be indicative of one or more selective agents in structuring the distribution of flower and fruit colors, although studies to date have focused on regional as opposed to global scales (Shrestha et al., 2014; Tai et al., 2020).

We here set out to explore whether and how abiotic climatic factors structure the global distribution of flower and fruit colors, using a data set of 2815 plant species across 51 clades (across 40 families and 23 orders; Appendix S1: Tables S2, S3). We focused on animal‐pollinated and animal‐dispersed (fleshy fruited) taxa to assess patterns of color variation in both flowers and fruits for the same species. We scored colors into eight categories and assessed patterns at the level of whole plant assemblages (10,097 1 × 1 degree grid cells) and single species (4,757,769 occurrences). Among the possible measures of color diversity, we chose Shannon's diversity index (Shannon, 1948) because it captures both the diversity of distinct colors in a community and their relative dominance (abundance) and has been found to perform well at the coarse resolutions used in our analyses (see more on performance and limitations in Qiao et al., 2024). Thus, a high Shannon diversity value would indicate an area with many distinct flower or fruit colors, which are all equally well represented, and a low Shannon diversity value would correspond to an area where only one or a few colors dominate the species pool. Since flower colors are generally skewed toward white, and fruit colors toward black and red (Delmas et al., 2020), an area of high Shannon diversity values for flower and fruit color would hence correspond to an area with relatively more non‐white flowers and non‐black and non‐red fruits.

Documenting changes in the Shannon diversity index for color and associations of distinct colors with distinct environmental stressors across large ecogeographic scales would be indicative of a strong effect of the abiotic environment in structuring the distribution of flower and fruit colors. With the known protective function of color pigments from abiotic stressors (e.g., frost, heat, drought, high UV‐B), we may envision two different patterns of color diversity in association with environmental factors. First, if a distinct color offers stronger protection from an abiotic stressor, we may expect selection to favor this color in environments where this stressor is dominant (Coberly and Rausher, 2003; Koski and Galloway, 2018; Vaidya et al., 2018), thereby increasing the relative abundance of this color and, if this color becomes dominant in the community, resulting in low Shannon diversity. Alternatively, it is plausible that various colors (potentially produced by various different pigments such as betalains, carotenoids, anthocyanins) can contribute to ameliorating stress, in which case we may expect elevated color diversity in extreme environments (i.e., potentially through increased production of pigments already expressed in non‐reproductive tissues; Jain and Gould, 2015). With our global flower and fruit color data set, we set out to characterize patterns of diversity and address three broad questions. First, is color diversity correlated between flowers and fruits? Correlated variation would indicate similar (biotic or abiotic) selective agents, while a lack of correlation would indicate that flower color diversity is shaped by different factors than fruit color diversity. Since we found a strong decoupling of flower and fruit color diversity, we next asked whether this lack of correlation can be explained by differential impacts of three abiotic factors (temperature, aridity, and UV‐B stress) that strongly affect plant pigmentation. The effects of these stressors on color diversity may be particularly strong in areas where they act in combination (i.e., increased cold and UV‐B stress in mountains and arctic ecosystems, increased heat and UV‐B stress in deserts). Finally, to resolve whether these different patterns are caused by distinct responses of individual colors to abiotic stressors, we analyzed how individual colors vary across space and environments. Through this set of analyses, we uncovered strong relationships between color diversity and the abiotic environment in addition to striking environment–color associations, although these varied markedly for flowers and fruits, consistent with their distinct roles in plant physiology and reproduction.

## MATERIALS AND METHODS

All analyses were run in R version 4.3.0 (R Core Team, 2023).

### Flower–fruit color data set

We based our study on the global data set of fruit colors of Sinnott‐Armstrong et al. (2018). From this data set, we selected only clades which are animal‐pollinated and animal‐dispersed and for which at least 10% of the total species in the clade had been covered (based on estimates of Christenhusz et al., 2017). We further added *Gaultheria*, *Cornus*, and *Scaevola*, yielding 51 clades across 40 plant families and 23 orders (Appendix S1: Tables S1, S2). For these clades, we extracted all taxa included in the phylogeny by Jin and Qian (2019), yielding a candidate list of 3250 taxa.

Fruit color data was readily available from Sinnott‐Armstrong et al. (2018) for 1339 taxa, scored into eight categories (black, blue, red, white, green, yellow, orange, brown). For all remaining taxa, we scored fruit color into these categories using species descriptions, published data sets (Lu et al., 2019; Lindelof et al., 2020; Hilgenhof et al., 2023) and iNaturalist images (https://www.inaturalist.org/). For flower colors, we searched for photos of each taxon in the databases of the Global Biodiversity Information Facility (GBIF; https://www.gbif.org/) or iNaturalist and scored colors into 45 categories using a color reference chart (Appendix S1: Figure S1). We only used images that were not clearly over/underexposed. To make our flower and fruit color data sets comparable, we had to summarize colors into the same number of categories. Given that fruit colors are commonly summarized into eight categories (Sinnott‐Armstrong et al., 2018), we summarized flower colors also into eight categories as done for other macroecological studies that categorized flower color (e.g., Delmas et al., 2020); fully white and light colors were categorized as “white” and with other categories being orange, red, pink, purple, yellow, green and dark (for details and discussion on alternative categorization, see Appendix S1: Figure S1, Table S3). In total, we scored 2890 taxa for both flower and fruit color.

### Collation and cleaning of occurrence data

To obtain occurrence records, we submitted the list of 2890 taxa to the GBIF portal (May 2023) through the rgbif interface (Chamberlain et al., 2023) and used custom filtering techniques implemented in CoordinateCleaner (Zizka et al., 2019; for details, see Appendix S1: Supplementary methods). We repeated the search for all synonyms identified through the R package Taxonstand (Zhang and Qiang, 2023). To filter out potential invasive ranges, we next submitted the taxa list to the GloNAF database (vanKleunen et al., 2019). We identified 314 taxa as invasive in a part of their range and removed the occurrences in the respective invasive ranges (9,338,392 records removed, leaving 10,773,711 records). After these filtering steps, our list included 2853 taxa with georeferenced localities.

Next, we plotted range maps for each clade (Appendix S1: Figure S2) and compared clade ranges to range information available on the Plants of the World Online database (https://powo.science.kew.org/, accessed August 2023). We manually removed occurrences outside of the documented clade ranges, screened for species commonly used in cultivation, and removed occurrences in cultivated ranges. For large clades with occurrences in the United States, Europe, and Australia (with herbaria often housing non‐native species), we additionally verified that no non‐native taxa were included. These cleaning steps reduced the data set to 6,169,815 records across 2815 taxa (Appendix S1: Table S1), and 4,757,769 records when only including one occurrence per species per 30 arc second (1 × 1 km) grid cell.

As a final assessment of the accuracy of our records, we calculated species richness for 1 × 1 degree grid cells (ca. 110 km at equator, 12,508 grid cells) using the function RichnessGrid in the R package speciesgeocodeR (Zizka and Antonelli, 2016; Töpel et al., 2017). We manually checked the resulting species lists in all grid cells (14) with more than 180 records to assure that these did not pertain to institution records but represent true centers of biodiversity (Appendix S1: Supplementary methods). For subsequent analyses, we subset the data set to only include grid cells with a minimum of three (10097 grid cells), five (8514 grid cells) or 10 (5979 grid cells) species. In our final 1 × 1 degree data set with a minimum of three species, each species occupied between 1 and 2658 grid cells, with a mean of 75 and a median of 29 grid cells. The maximum number of species per grid cell was 224, with a mean of 20 and a median of 12 species per 1 × 1 degree grid cell.

### Environmental variables

To test for color–environment associations, we downloaded the global shapefile for major world biomes designated by WWF (Olson et al., 2001) and extracted the biome category for each occurrence point (over, sf; Pebesma and Bivand, 2023). Next, we downloaded data on mean annual temperature (°C) from WorldClim at 30 arc‐sec resolution (ca. 1 km at the equator; Fick and Hijmans, 2017). For aridity, we retrieved data on the global aridity index (AI) from Zomer et al. (2022), calculated as mean annual precipitation divided by mean annual reference evapotranspiration (Trabucco and Zomer, 2018). The AI is a good estimate for closed versus open canopy biomes, thus capturing potential drought and light stress. A higher AI indicates more mesic biomes (Trabucco and Zomer, 2018). For UV‐B, we downloaded data on mean annual global UV‐B radiation (15 arc‐min resolution) from Beckmann et al. (2014). We extracted all climatic variables for each species' occurrences (4,757,769) in the 30 arc‐sec grid.

Next, for each of the 10,097 1 × 1 degree grid cells containing a minimum of three species, we calculated mean climatic variables based on the climatic values extracted for each species' occurrences in the 30 arc‐sec grid. We removed 20 grid cells with missing data, leaving 10,077 grid cells. To incorporate effects of evolutionary history into analyses (i.e., higher color similarity among closely related species), we calculated the mean phylogenetic distance (MPD, based on the pairwise cophenetic branch lengths distances of all taxa in the grid cell) for each grid cell using the mpd function in the R package picante (Kembel et al., 2010).

### Shannon diversity index for flower and fruit colors

For the 10,077 grid cells containing a minimum of three species, we calculated the Shannon diversity index of flower and fruit colors, treating color category as “species” and species number per grid cell as “abundance” (diversity function in R package vegan, Oksaanen et al., 2017). We further calculated the difference between the two diversity indices by subtracting fruit from flower color diversity, with a positive value indicating higher flower color diversity and a negative value higher fruit color diversity. To visualize patterns, we plotted diversity indices onto global maps. To evaluate the impact of different flower color categorization schemes (Appendix S1: Table S3), we also calculated flower color diversity for the different categorization methods, plotted results, and ran correlation tests.

Next, we tested whether biomes differ in color diversity using Kruskal–Wallis ANOVAs (uneven variance among biomes). We used Dunn's test as post hoc tests, with a Bonferroni correction to account for multiple comparisons. To assess whether color diversity is a mere function of species or clade richness, we ran additional correlation tests.

Finally, to test whether the distribution of the Shannon diversity values for flower and fruit color can be explained by abiotic factors, we employed boosted regression trees (BRT), a machine‐learning based ensemble method suitable for (often skewed) ecological data (Elith et al., 2008; Appendix S1: Supplementary methods). We employed the gbm.step function (R package dismo; Hijmans et al., 2023) to fit BRT through 10‐fold cross‐validation (data is split into 10 subsets), using either flower color diversity, or fruit color diversity, or difference in color diversity as response, and the abiotic environmental variables mean annual temperature, aridity index, UV‐B irradiance, and phylogenetic diversity per grid cell as predictors. We ran models for grid cells with a minimum of three, five, and 10 taxa to assess potential bias related to low species numbers per grid cells (Appendix S1: Figure S3; Sabatini et al., 2022). We chose this approach to retain the relatively fine‐grained character of our analysis (100 × 100 km) to more accurately assess the impact of environmental factors on color diversity. An alternative approach not pursued by us could be choosing a grid with a coarser resolution (e.g., 200 × 200 km, 500 × 500 km), which would ultimately lead to more species per grid cell and could hence alleviate issues related to low species numbers but would result in more environmental heterogeneity per cell.

Using the general recommendations of Elith et al. (2008), we fit initial models of tree complexity 5, a learning rate of 0.01, a bag fraction of 0.5, and a Gaussian family distribution for each data set. Next, we systematically varied the parameters by gradually reducing tree complexity to 1 (to avoid overfitting of models), reducing the learning rate to 0.001, and increasing the bag fraction to 0.75. We used the cross‐validation statistics to evaluate model performance and to choose model parameters with minimal predictive deviance. Overall, models with a learning rate of 0.01, tree complexity of 5, and bag fraction of 75% had the lowest predictive deviance (Appendix S1: Table S4). Next, we explored whether the relative importance of predictors changes across different parameter settings. Since models based on grid cells including a minimum of three species sometimes gave different results than models based on a minimum of five or 10 species, we report results for all data sets. Finally, we checked whether interactions were detected among predictors, using the function gbm.interactions.

### Distribution of distinct flower and fruit colors

To explore the global distribution of distinct colors, we calculated 1 × 1 degree grid‐cell species richness for each flower and fruit color category separately. We scaled each color's grid cell's species richness by the corresponding grid cell's total species richness (Appendix S1: Figure S3), thereby creating a proportional index of color richness (1, all species in the cell of a certain color; 0, no species in the cell of a certain color). We visualized these patterns by plotting the scaled color richness grids and the most dominant color per grid cell onto global maps. We further split the data set into the different WWF biomes and visualized the relative color composition of flowers and fruits in each biome using bar plots. We repeated this step for the different flower color categorization schemes (Appendix S1: Table S3) to assess potential caveats of color categorization. Finally, we tested whether the abundance of a specific color within a biome differs significantly from random expectations using *χ*² tests.

To explicitly test whether temperature, aridity or UV‐B irradiance explain differences in the distribution of flower and fruit colors, we used multinomial logistic regressions. We treated flower or fruit color category as the response variable, specifying “white” as the reference for flowers (the most common color) and “green” as the reference for fruits (since most fruits start out as green, see Sinnott‐Armstrong et al., 2018). We included plant clade as a random effect grouping variable to account for phylogenetic non‐independence. We started out with a model including an interaction between all climatic variables and compared this model using Akaike's information criterion (AIC) against models including additive effects or single variables only (Appendix S1: Table S5). We fit models using the mblogit function (R package mclogit; Elff, 2022) and extracted estimated marginal means for the fitted models to plot the effects of climatic variables on flower and fruit colors (Lenth, 2023). We ran initial models averaging each climatic variable across each species' occurrences and then repeated analyses 100 times by randomly selecting a single occurrence and its associated climatic variables per species, thereby alleviating bias through mid‐domain effects stemming from averaged variables. We evaluated whether models based on species averages gave the same results as models using single occurrences.

## RESULTS

### Is the Shannon diversity index for color correlated between flowers and fruits?

**Figure 1:**
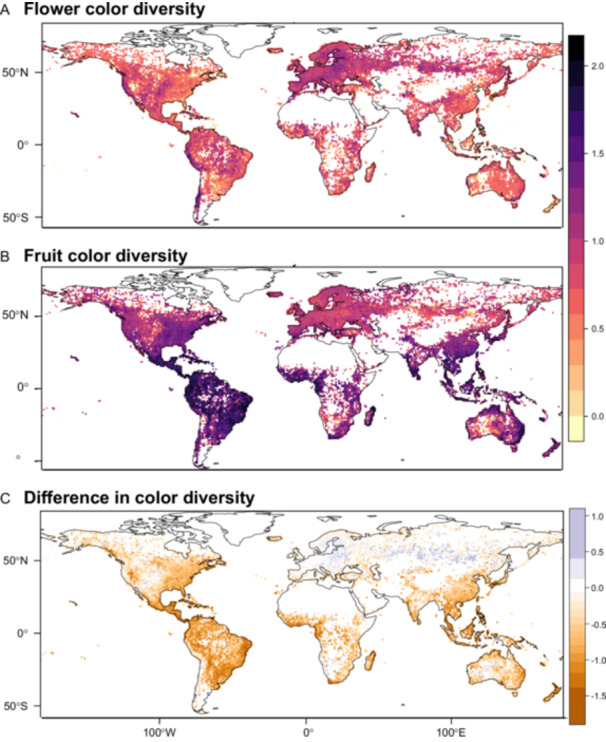
Shannon diversity index for (A) color of flowers and (B) fruits and (C) the difference between the diversity indices for flower and fruit color. Flower color diversity is consistently lower than fruit color diversity in most areas of the world, but most prominently in the tropics, where fruit color diversity is consistently high. Flower color diversity is slightly higher than fruit color diversity in some areas in the palearctic, wet areas in southwestern South America, and arid regions of the central United States, southwestern Africa and central Australia.

Binning flower and fruit colors into eight categories each, we found that Shannon diversity values for flower and fruit color were globally uncorrelated (Figure 1; Appendix S1: Figure S4, *R*² –0.096). The flower color diversity values (0–1.67) were generally lower than the fruit color diversity values (0–2.03). Tropical environments had lower flower color diversity than temperate environments (tropical vs. temperate: *χ*² 190, *P* < 0.01), indicative of a more even distribution of different color categories in temperate environments. In fruits, in contrast, color diversity was higher in tropical environments (*χ*² 1998, *P* < 0.01, Appendix S1: Table S6, Figure S5). Flower color diversity showed only weak correlations to species richness (*R*² 0.147) and clade richness (*R*² 0.248), but fruit color diversity was positively correlated with species (*R*² 0.745) and clade richness (*R*² 0.645, Appendix S1: Figure S4).

For flowers, different color categorization schemes (i.e., keeping light colors as separate categories or lumping all colors into their main category irrespective of saturation) affected the distribution of the Shannon diversity values and led to an overall increase in color diversity in the tropics (more even distribution of different colors; Appendix S1: Figure S6). Generally, however, color diversity was positively correlated across our categorization schemes (keeping light colors separate: *R*² 0.61; lumping all colors into main color category irrespective of saturation: *R*² 0.56).

### Do abiotic factors differently affect Shannon diversity values for flower and fruit color?

**Figure 2:**
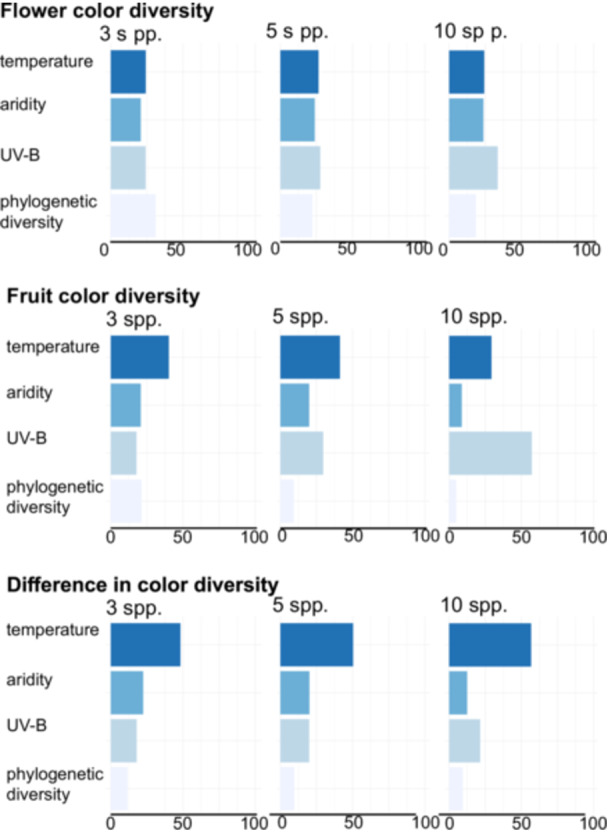
Relative influence of predictors on patterns of color diversity for boosted regression trees based on grid cells with a minimum of three, five, or 10 species. All three abiotic stressors and phylogenetic diversity influenced flower color diversity to a similar extent, with highest flower color diversity values at low and high UV‐B exposure, intermediate temperatures, and high phylogenetic diversity (Appendix S1: Figure S6). Fruit color diversity was most strongly influenced by mean annual temperature (three and five species) or UV‐B exposure (10 species, more grid cells in lower latitudes), with highest fruit color diversity under high UV‐exposure in dry and warm environments (Appendix S1: Figure S6). The difference between flower and fruit color was influenced most strongly by temperature, capturing the marked latitudinal gradient of fruit color diversity, with the difference being strongest under warm conditions and high UV‐B exposure and smallest at intermediate temperatures with low UV‐B exposure and high phylogenetic diversity (Appendix S1: Figure S6).

For flower color, Shannon diversity index was best explained by a combination of climatic factors, with mean annual temperature, aridity index, UV‐B irradiance and phylogenetic diversity all being important (ranging between 15% and 25% in importance), and varying in ranking depending on whether our analyses were based on a data set with a minimum of three, five, or 10 species per grid cell (Figure 2; Appendix S1: Table S7). Flower color diversity peaked at intermediate temperatures (ca. 10°C) in localities with either low or high UV‐B irradiance and generally increased with increasing phylogenetic diversity (Appendix S1: Tables S7 and S8, Figure S7).

In contrast to this relatively even contribution of multiple factors, fruit color diversity was most clearly explained by mean annual temperature (models based on a minimum of three or five species: ca. 40%, Figure 2; Appendix S1: Tables S7, S8) or UV‐B irradiance (models based on a minimum of 10 species, ca. 57%). Fruit color diversity peaked under warm conditions with high UV‐B irradiance (lowest at low temperatures and low UV‐B irradiance) and was highest in wet environments with high UV‐B irradiance (Appendix S1: Figure S7).

Finally, differences in flower and fruit color diversity were most clearly explained by temperature across all models (48–57%, Figure 2). The differences (with much higher fruit than flower color diversity) were strongest in warm areas with high UV‐B irradiance and smallest (almost equal flower and fruit color diversity) in cool environments with low UV‐B irradiance (Appendix S1: Figure S7, Tables S7, S8).

### Do different abiotic stressors explain the distribution of distinct flower and fruit colors?

**Figure 3:**
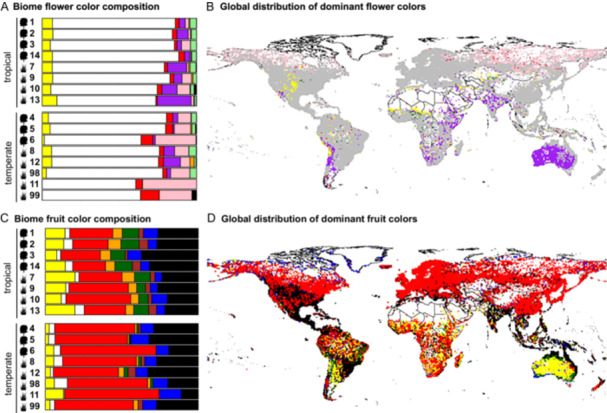
Marked differences in flower and fruit color composition between tropical and temperate environments (data subsampled to one occurrence per species per 1 × 1 degree grid cell, also see Appendix S1: Figure S7). (A, B) In flowers, white (grey on map) is the dominant color across environments (summarized here as biomes), but the mostly anthocyanin‐based colors red and pink are increasingly common at higher latitudes (biomes 6, 11, 99), while yellow and purple are more common in arid grasslands both in the tropics (biomes 7, 10, 13) and temperate zone (biomes 8, 12). (C, D) In fruits, red and black are the most common colors, with red becoming particularly dominant at higher latitudes (temperate biomes), while tropical biomes show a more even distribution of all fruit colors; yellow fruits are more common in arid grasslands (biomes 7, 13). Biomes: 1, tropical and subtropical moist broadleaf forests; 2, tropical and subtropical dry broadleaf forests; 3, tropical and subtropical coniferous forests; 4, temperate broadleaf and mixed forests; 5, temperate coniferous forests; 6, boreal forests, taiga; 7, tropical and subtropical grasslands, savannas and shrublands; 8, temperate grasslands, savannas and shrublands; 9, flooded grasslands, savannas and shrublands; 10, montane grasslands, savannas and shrublands; 11, tundra; 12, Mediterranean forests, woodlands, and scrub or sclerophyll forests; 13, deserts and xeric shrublands; 14, mangroves; 98, lakes; 99, Arctic (rock and ice/Greenland). Tree icons represent forested habitats; grass icons represent non‐forested habitats.

Individual flower and fruit colors showed non‐random patterns in their global distribution (Figure 3; Appendix S1: Table S9). As expected based on previous studies (Delmas et al., 2020; Tai et al., 2020), among flowers, white was the dominant color across 71% of grid cells and found in all environments. However, pink and red flowers were more common at high latitudes (Figure 3A) and the dominant colors in 8.7% and 0.6% of grid cells, respectively (Figure 3B). Purple and yellow flowers were more common in open canopy environments (i.e., savannas, scrublands, deserts, mountains; Figure 3A; Appendix S1: Figures S8–S11) and dominant in 7.6% and 2.2% of grid cells (Figure 3B), respectively. These patterns were consistent when categorizing flower colors differently (Appendix S1: Figure S13): White remained the most common color across environments, but in lower relative proportion (reduced from ca. 82% of species to ca. 50% (lumping color shades into main color category) and 55% (retaining light color categories separately) (Appendix S1: Table S3). Pink and red flowers continued to be most common in temperate environments, with mostly unchanged relative abundance (Appendix S1: Figure S13). The largest effect of different categorization schemes was seen among light‐yellow and light‐green flowers and, to a lesser extent, among light‐purple flowers, which increased in relative abundance in tropical environments (Appendix S1: Figure S13).

**Figure 4:**
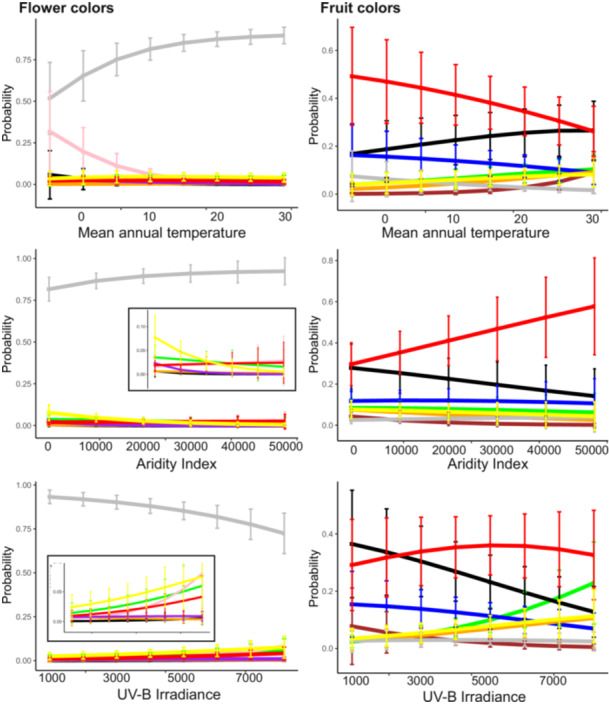
Multinomial logistic regression models showing the probability of finding distinct flower or fruit colors in relation to mean annual temperature, aridity index (higher indicates wetter conditions), and UV‐B irradiance, including plant clade as random effects grouping variable. In flowers, white flowers are generally most common, but pink flowers are associated with low temperatures and increased UV‐B irradiance, yellow and green flowers with arid climates and high UV‐B irradiance, red flowers with high UV‐B irradiance in both dry and humid habitats, and purple flowers with dry habitats. Inserts in the flower color plots are close‐ups of the *y*‐axis to better display the probability of non‐white flower colors. In fruits, red fruits are more common in cold and wet environments and under intermediate UV‐B irradiance; black fruits are more common in warm and dry environments and under low UV‐B irradiance. Green, yellow, and orange fruits are more common under high UV‐B irradiance in warm environments; blue fruits are associated with low temperatures and low UV‐B irradiance.

Our multinomial logistic regression models supported the hypothesis that different abiotic environmental stressors are associated with different flower colors (Figure 4). Specifically, pink flowers were significantly more common in colder temperatures (*z* –6.3, *P* < 0.001, 100/100 randomly resampled data sets), and both pink and red flowers were significantly more common under increased UV‐B irradiance (Figure 4, pink: *z* 5.5, *P* < 0.001, 100/100 resamples; red: *z* 2.3, *P* < 0.05, 54/100 resampled data sets, Appendix S1: Table S10). Purple flowers increased in abundance with increasing aridity and under lower temperatures (*z* –2.7, *P* < 0.01, 84/100 resamples, Figure 4), and yellow flowers peaked in abundance in dry and warm conditions (*z* –2.6, *P* < 0.01, 61/100 resamples, Figure 4). The most pigmented (“dark”) flowers were associated with low temperatures (*z* –2.5, *P* < 0.05, 56/100 resamples).

In fruits, differences in color distribution could mostly be attributed to latitudinal differences between tropical and temperate environments (Figure 3). Red and black fruits were generally the most common colors across environments and dominant in 49.2% and 22.5% of grid cells, respectively (Figure 3D). Tropical environments had comparatively higher proportions of yellow, green, orange, and brown fruits, with these colors dominant in 9.5% (yellow), 2.3% (green), 1.7% (orange), and 0.5% (brown) of grid cells (Figure 3C, D; Appendix S1: Figures S8–S10, S12). Multinomial logistic regressions revealed that UV‐B irradiance was the main driver of fruit color distribution, with black, blue, and brown fruits significantly less common under high UV‐B irradiance (Figure 4; black: *z* –3.7, *P* < 0.001, 100/100 resamples; blue: *z* –3.2, *P* < 0.01, 70/100 resamples; brown: *z* –2.5, *P* < 0.05, 50/100 resamples). Red fruits were significantly more common in cool and wet environments of intermediate UV‐B irradiance (temperature × UV: *z* 2.5, *P* < 0.05, 65/100 resamples, aridity × UV: *z* 2.4, *P* < 0.05, 22/100 resamples, Appendix S1: Table S10).

## DISCUSSION

Although flowers and fruits are developmentally continuous and use the same classes of pigments to produce colors, we here showed that flower and fruit color had vastly different macroecological patterns and abiotic drivers of those patterns. This decoupling of flower and fruit color is true both at the level of whole plant assemblages (decoupling of flower and fruit color diversity) and at the level of single species (different associations between distinct color categories and abiotic factors). Importantly, the large‐scale ecogeographic associations between abiotic factors (temperature, aridity, UV‐B irradiance) and distinct flower and fruit colors suggest that the abiotic environment may, to some extent, constrain pollinator‐ and disperser‐mediated selection on flower and fruit colors. Our results also indicate, however, that coloration patterns of flowers and fruits cannot be explained by a unified ecogeographic rule such as the color rules proposed for animals (Lev‐Yadun, 2015; Delhey, 2019). Quantifying the relative importance of biotic and abiotic agents of selection will thus be essential in the future to identify the “rules” underpinning the non‐random distributions of flower and fruit colors.

Spatial patterns in the Shannon diversity values for color resulted not only from abiotic factors that might increase or decrease diversity, but also from the factors that independently drive the evolution of individual colors. The global decoupling of the Shannon diversity values for flower and fruit color at the assemblage level supports the idea that different mechanisms structure the large‐scale distribution of flower and fruit colors. If the abiotic environment were the paramount driver of color diversity across both organs, then we would have expected joint variation in diversity patterns. The documented lack of co‐variation may be rooted in the general difference of which color is dominant in flowers (white) and fruits (red). At the scale of our analyses (100 ×100 km grid cells), we observed increased flower color diversity in grid cells encompassing a high proportion of environments of high abiotic stress (cold, drought and UV‐B stress in (arid) grasslands and mountain ranges on the west coast of North and South America, Great Plains in the United States, temperate Europe, Eurasian steppe). In these grid cells, the relative proportion of white was reduced in favor of other colors (pink, red, purple, yellow), suggesting that different classes of pigments can all contribute to stress responses. In fruits, on the other hand, cold temperate environments were dominated by one (red) color only, resulting in a low Shannon diversity value and suggesting that this color is disproportionately selected in cold environments. In comparison, fruit color diversity peaked in the (wet) tropics (lower relative abundance of red), possibly indicating lower selection for red and/or increased selection for other colors. Accordingly, we observed temperature as best explaining this latitudinal diversity gradient. Since species and clade richness were both strongly associated with fruit color diversity, however, this association between temperature and fruit color diversity may result more from a similar latitudinal pattern than a causal relationship.

Our results suggest that distinct environmental stressors select for distinct colors in flowers. As expected (Kevan et al., 1996; Delmas et al., 2020), white was the dominant color across environments, but white flowers decreased in relative abundance in cold, dry, or high UV‐B environments. Pink, dark, and red flowers increased in relative abundance in cold environments, while dry and high UV‐B environments associated with more purple and yellow flowers (Figures 3, 4; Appendix S1: Figure S13). The pigments producing these colors (commonly anthocyanins for pink, red, and purple flowers and carotenoids or chalcones for yellow; Narbona et al., 2021) carry protective functions against abiotic stressors (Gould, 2004) and may greatly affect fitness (Rausher, 2008). Increased pigmentation in flowers, for example, may mitigate cold stress by increasing floral temperature (i.e., after chilly nights; Tikhomirov et al., 1960), thereby also increasing attractiveness to pollinators (i.e., through increased scent release; Farré‐Armengol et al., 2020). Increased accumulation of flavonoids (including anthocyanins) together with hydroxycinnamates (together forming UV‐absorbing phenylpropanoids) may further help protect the photosynthetic apparatus against photodamage under high UV‐B exposure (“sunscreen”) and help control water loss by reducing stomatal transpiration under drought stress (Schemske and Bierzychudek, 2001; Cirillo et al., 2021; Narbona et al., 2024). Anthocyanins are also excellent antioxidants, which can scavenge free radicals generated by various abiotic stressors, including heat, frost, and UV‐B (Li and Ahammed, 2023). The strong associations between distinct colors and abiotic stressors documented here align well with these protective functions of pigments and raise exciting questions on eco‐evolutionary relationships. For example, are flower colors freer to adapt to the visual preferences of their pollinators in environments of low environmental stress compared to stressful environments (where they might be constrained by selection on their protective function)?

The environmental associations of flower colors are only partially matched for fruit colors, indicating that the same colors in fruits might have evolved in response to different selective pressures. While the prevalence of vibrant red (anthocyanins and/or carotenoids), black and blue (both typically flavonoids) fruits in cold environments is consistent with the idea that increased pigmentation may aid in thermoregulation (Espley and Jaakola, 2023), it does not explain the dominance of black fruits (which have the highest concentration of pigments [proanthocyanins]) in warm environments. Similarly, the higher proportion of dull fruits (yellow, orange, green, brown; from chlorophyll and/or carotenoids; Khoo et al., 2011) in association with high UV‐B exposure in the tropics seems surprising and does not match the idea that increased pigmentation acts as a “sunscreen”. Furthermore, carotenoids and chlorophylls do not absorb UV‐B (but note that fruits often contain phenolic compounds, which may capture UV‐B; Krebs and Schummer, 2024). One possible explanation for the relative abundance of non‐UV absorbing pigments in fruits is that pollen grains and ovules are more exposed to UV‐B radiation than seeds (Zhang et al., 2014; Koski and Galloway, 2018), and thus selection to protect reproductive organs is stronger in flowers than in fruits. Alternatively, this pattern might result from an interaction between temperature and UV‐B. In naturally hot, tropical environments, high sun exposure might intensify heat stress and select for less pigmented fruits as a mechanism to avoid desiccation (see also Whitney and Lister, 2004; Espley and Jaakola, 2023). This idea is matched by the fact that black fruits are most strongly associated with low UV‐B and less‐pigmented fruits (desiccation avoidance) with high UV‐B.

Although biotic interactions are commonly regarded as primary drivers of flower and fruit color evolution (Trunschke et al., 2021), the strong associations with abiotic factors underscore that mutualists are not the only selective agents on the global distribution of flower and fruit colors (Dalrymple et al., 2020). In this context, the global decoupling of flower and fruit color diversity is particularly interesting. If biotic interactions (rather than stressful environments) were most important in driving the global‐scale diversity of flower and fruit colors, we might have expected correlated diversity peaks in the tropics, where the functional diversity of mutualists with different visual systems (and hence the capacity to select for different colors) is highest (Ollerton et al., 2017; Sinnott‐Armstrong et al., 2021). Although a test of this hypothesis is currently lacking, a baseline expectation for a strong impact of mutualists on Shannon diversity values for color might be higher diversity because more different mutualists select for more different colors and thereby increase evenness among colors. Regardless of which flower color categorization scheme we used (Appendix S1: Figures S6, S13), however, such a tropical increase in evenness was only found in fruits. While this pattern does not replace a future test of the relative importance of biotic and abiotic agents of selection on fruit color, it is noteworthy that green, yellow, orange, and brown fruits (more common in the tropics in our data set) are generally associated with mammal dispersers (disperser syndrome hypothesis; Valenta and Nevo, 2020), which are most important in the tropics. Such patterns are not paralleled in flowers, where, for example, red flowers classically associated with bird pollination (most common in the tropics, Chen et al., 2020) peak in temperate zones (see León‐Osper and Narbona, 2022 for a similar red–temperate association). Clearly, evaluating to what extent abiotic and biotic agents of selection jointly or separately drive the distribution of flower and fruit colors represents an important next step (Dalrymple et al., 2020).

Our finding of distinct environment–color associations underlines the value of recording color into distinct categories (with caveats, see below). While the color rules proposed for animals are based on estimates of “darkness” (i.e., averages across green/blue/red color channels), they likely gloss over the potential function of distinct colors in distinct environments. This lumping of colors into darkness estimates may, in part, explain the controversy around color rules in animals (Delhey et al., 2019). The fact that Gloger's rule, as initially formulated, holds different predictions on environment associations for black phaeo‐melanins (common where wet and warm) and rufous eu‐melanins (common where warm and dry) indicates that also in animals, distinct pigments may have different functions in distinct environments. Even with distinct colors associating with distinct environments, however, large‐scale quantification of flower and fruit pigmentation may help clarify whether pigmentation *within* color categories varies with abiotic factors and hence is indicative of general pigmentation rules. Based on our results, for example, we hypothesize that pink and red flowers become increasingly darker pink and red as cold stress increases, while pigmentation in pink, yellow and purple flowers increases with increasing UV‐B stress and heat (i.e., Appendix S1: Figure S13). In fruits, we hypothesize increasing pigmentation in red, blue, and black fruits in cold environments as frost protection, but decreasing pigmentation in yellow, orange, brown and green fruits under heat stress to avoid desiccation. Testing whether these predictions hold true at large spatial scales represents a critical step to clarify the impact of the abiotic environment in structuring flower and fruit coloration.

### Limitations of the study and suggestions for future studies

Several important caveats in our data set warrant further explanation and future examination with expanded data sets. First, for the broad sampling across clades and geographic areas that we attempted, collecting information on flower and fruit colors for the same species required combining various scoring techniques (from images, species descriptions, herbarium labels). While we followed common practices in binning flower and fruit colors into eight categories each (e.g., Sinnott‐Armstrong et al., 2018; Delmas et al., 2020), binning a naturally quantitative trait such as color always introduces bias. For example, scoring colors from images (such as done for flowers here) suffers from caveats such as enhanced red tones (making images more appealing to human vision; Stevens et al., 2007). The fact that our findings of increased pink and red flowers in temperate environments and yellow and purple flowers in dry and high UV environments hold across different color categorization schemes (Appendix S1: Figure S13) is reassuring in that the reported ecogeographic patterns of coloration are not mere artifacts of data categorization. The resulting differences in the Shannon diversity values for flower color, however, highlight the importance of follow‐up studies to record and categorize flower and fruit colors the same way and evaluate the impact of different categorization schemes on both organs.

Second, an aspect which will always be missed when binning colors into categories is related to the continuous and multivariate nature of colors. Color properties such as hue, gloss, spectral purity, chroma and within‐ and across‐organ contrasts (i.e., petal patterning, petals versus androecium) play important roles in communication with biotic mutualists (van der Kooi and Spaethe, 2022; Lunau and Dyer, 2024). Whether they are functionally relevant and also vary with abiotic factors remains unknown, however. Further, different mutualists differ in their sensory capacities and see colors differently (Lomáscolo and Schaefer, 2010). Previous studies on flower and fruit colors have attempted to account for these perceptional differences by modelling (quantitatively measured) colors (i.e., spectral data) in the visual spaces of different mutualists (i.e., Stournaras et al., 2013). For macroecological studies, which combine species pollinated and dispersed by various different mutualists with different visual properties, however, there simply is no joint visual space which can be applied across all study taxa. For understanding the role of color pigments as protection against environmental stressors, however, we believe that, instead of measuring spectral data (with the inherent problem of choosing the appropriate visual space; Stournaras et al., 2013), measuring the concentration of pigments (i.e., flavonoids, carotenoids) might give a more informative and unbiased answer. Based off our results on color patterns, we may formulate clear expectations on the concentration of pigments (i.e., higher concentration of anthocyanins in frost‐prone environments in flowers, higher concentration of carotenoids in hot and dry environments in fruits), which may be tested across a broad set of taxa and habitats.

Third, with the strong association we documented between distinct colors and UV‐B exposure, it would be interesting to incorporate UV reflectance and absorbance into future analyses. Such an inclusion would again reshuffle the categorization of “white” flowers, since human‐vision white flowers are almost always strongly UV absorbent and strongly chromatic in bee visual space (Kevan et al., 1996). Further, although spatial variation with environmental factors is widely recognized for flowers (Koski and Ashman, 2015; Koski et al., 2020), it is less well known that UV reflectance in fruits is also common (Willson and Whelan, 1990; Middleton et al., 2024). Thus, incorporation of UV absorbance/reflectance through spectral measurements would provide valuable insight into its role in mitigating environmental stress in both organs.

Finally, we focused here on animal‐pollinated flowers and animal‐dispersed fruits, but many plant species do not interact with animals during reproduction (i.e., wind pollination and wind dispersal). Incorporating such species would likely provide significant insight into the relative importance of environment vs. biotic selection for coloration in plant reproductive structures. For example, demonstrating similar color–environment associations also for abiotically pollinated/dispersed species would greatly support the role of pigments as protective agents in sensitive floral tissues (i.e., Lacey et al., 2010). Furthermore, while our data set included more than 2800 species across 43 plant families, this sample still captures less than 1% of angiosperms. Even with this smaller sample, our species richness maps overall matched well with global patterns of vascular plant species richness (compare Appendix S1: Figure S3; Mutke and Barthlott, 2005), with slight under sampling in southern Africa and Southeast Asia. Although new AI tools will not solve limitations of color data collection from images outlined above, they may help increase sample sizes beyond our data set in the future to evaluate robustness of the patterns reported here (Elliott and Fortes, 2023).

## CONCLUSIONS

Taken together, the global distributions of flower and fruit colors seem to follow different color‐specific rules, with temperature, drought, and UV‐B stress explaining the distribution of pink, red, purple and yellow flowers, and a mix of heat and desiccation stress, potentially coupled with selection by mutualists (Valenta et al., 2018), may explain the higher proportion of green, yellow, brown, and orange fruits in the tropics. Our results highlight the need of more cross‐organ (i.e., flower‐fruit) and cross‐discipline (i.e., biotic and abiotic factors) investigations in the future to better resolve whether and how multiple, potentially opposing agents of selection shape the reproductive spectrum of angiosperms.

## AUTHOR CONTRIBUTIONS

A.S.D. and M.S.A. conceptualized the study; L.M. and S.D.S. coded the color data; A.S.D. ran the analyses; A.S.D., M.S.A., and S.D.S. wrote the manuscript.

## Supporting information


**Figure S1.** Color reference chart used to score flower colors from images.
**Figure S2.** Pruned ranges of study clades.
**Figure S3.** Species richness when considering all grid cells.
**Figure S4.** Shannon diversity values for flower (flavg) and fruit (fravg) color.
**Figure S5.** Color diversity (*y*‐axis) for the different biomes (top panels) and difference in flower and fruit color diversity (bottom panels) for biomes and realms.
**Figure S6.** Shannon diversity values for flower and fruit color.
**Figure S7.** The two strongest interactions between predictor variables of boosted regression tree models.
**Figure S8.** Flower and fruit color composition differed across tropical and temperate biomes.
**Figure S9.** Flower and fruit color composition mapped for the different biomes.
**Figure S10.** Flower and fruit color composition mapped for the different biomes.
**Figure S11.** Global distribution of individual flower colors of the 2815 species of fleshy‐fruited clades included in our data set.
**Figure S12.** Global distribution of individual fruit colors of the 2815 species of fleshy‐fruited clades included in our data set.
**Figure S13.** Comparison of the different color categorizations for flowers.
**Table S1.** Number of GBIF occurrences and species retained.
**Table S2.** Family and order information and DOI links to individual data sets downloaded from GBIF for each plant clade.
**Table S3.** Number of species per color category.
**Table S4.** Parameter optimization in BRT models on flower and fruit color diversity and difference in diversity.
**Table S5.** Model selection for multinomial regression models on flower and fruit color categories.
**Table S6.** Pairwise comparison (*z*‐values) of flower color diversity (lower diagonal) and fruit color diversity (upper diagonal) among biomes.
**Table S7.** Relative influence of the environmental variables mean annual temperature, aridity, UV‐B, and phylogenetic diversity on flower and fruit color diversity.
**Table S8.** Interactions between variables included in BRT models.
**Table S9.** Comparison of *χ*² residuals shows different patterns in the frequency of different flower and fruit colors among biomes.
**Table S10.** Parameters for multinomial regression model with lowest AIC.

## Data Availability

All code required to run the analyses and all relevant data sets have been deposited on the public repository Phaidra (https://phaidra.univie.ac.at/o:2098641).
