# Supplementary material for: Does the abiotic environment influence the distribution of flower and fruit colors?
Source: Am J Bot. 2025 May 14;113(1):e70044. doi: 10.1002/ajb2.70044 (PMC12816447; doi:10.1002/ajb2.70044)
Supplement: Supplementary file 1 — Figure S1. Color reference chart used to score flower colors from images. Figure S2. Pruned ranges of study clades. Figure S3. Species richness when considering all grid cells. Figure S4. Shannon diversity values for flower (flavg) and fruit (fravg) color. Figure S5. Color diversity (y‐axis) for the different biomes (top panels) and difference in flower and fruit color diversity (bottom panels) for biomes and realms. Figure S6. Shannon diversity values for flower and fruit color. Figure S7. The two strongest interactions between predictor variables of boosted regression tree models. Figure S8. Flower and fruit color composition differed across tropical and temperate biomes. Figure S9. Flower and fruit color composition mapped for the different biomes. Figure S10. Flower and fruit color composition mapped for the different biomes. Figure S11. Global distribution of individual flower colors of the 2815 species of fleshy‐fruited clades included in our data set. Figure S12. Global distribution of individual fruit colors of the 2815 species of fleshy‐fruited clades included in our data set. Figure S13. Comparison of the different color categorizations for flowers. Table S1. Number of GBIF occurrences and species retained. Table S2. Family and order information and DOI links to individual data sets downloaded from GBIF for each plant clade. Table S3. Number of species per color category. Table S4. Parameter optimization in BRT models on flower and fruit color diversity and difference in diversity. Table S5. Model selection for multinomial regression models on flower and fruit color categories. Table S6. Pairwise comparison (z‐values) of flower color diversity (lower diagonal) and fruit color diversity (upper diagonal) among biomes. Table S7. Relative influence of the environmental variables mean annual temperature, aridity, UV‐B, and phylogenetic diversity on flower and fruit color diversity. Table S8. Interactions between variables included in BRT models. Table S9. Compa [file AJB2-113-e70044-s001.docx]

**Does the abiotic environment influence the distribution of flower and fruit colors?**

Agnes S. Dellinger, Leah Maier, Stacey Smith, Miranda Sinnott-Armstrong

**Appendix S1.** Supplementary figures and tables.

**Figure S1.** Color reference chart used to score flower colors from images.

**Figure S2.** Pruned ranges of study clades.

**Figure S3.** Species richness when considering all grid cells.

**Figure S4.** Shannon diversity values for flower (flavg) and fruit (fravg) color.

**Figure S5.** Color diversity (*y*-axis) for the different biomes (top panels) and difference in flower and fruit color diversity (bottom panels) for biomes and realms.

**Figure S6.** Shannon diversity values for flower and fruit color.

**Figure S7.** The two strongest interactions between predictor variables of boosted regression tree models.

**Figure S8.** Flower and fruit color composition differed across tropical and temperate biomes.

**Figure S9.** Flower and fruit color composition mapped for the different biomes.

**Figure S10.** Flower and fruit color composition mapped for the different biomes.

**Figure S11.** Global distribution of individual flower colors of the 2815 species of fleshy-fruited clades included in our data set.

**Figure S12.** Global distribution of individual fruit colors of the 2815 species of fleshy-fruited clades included in our data set.

**Figure S13.** Comparison of the different color categorizations for flowers.

**Table S1.** Number of GBIF occurrences and species retained.

**Table S2.** Family and order information and DOI links to individual data sets downloaded from GBIF for each plant clade.

**Table S3.** Number of species per color category.

**Table S4.** Parameter optimization in BRT models on flower and fruit color diversity and difference in diversity.

**Table S5.** Model selection for multinomial regression models on flower and fruit color categories.

**Table S6.** Pairwise comparison (*z*-values) of flower color diversity (lower diagonal) and fruit color diversity (upper diagonal) among biomes.

**Table S7.** Relative influence of the environmental variables mean annual temperature, aridity, UV-B, and phylogenetic diversity on flower and fruit color diversity.

**Table S8.** Interactions between variables included in BRT models.

**Table S9.** Comparison of *χ*² residuals shows different patterns in the frequency of different flower and fruit colors among biomes.

**Table S10.** Parameters for multinomial regression model with lowest AIC.


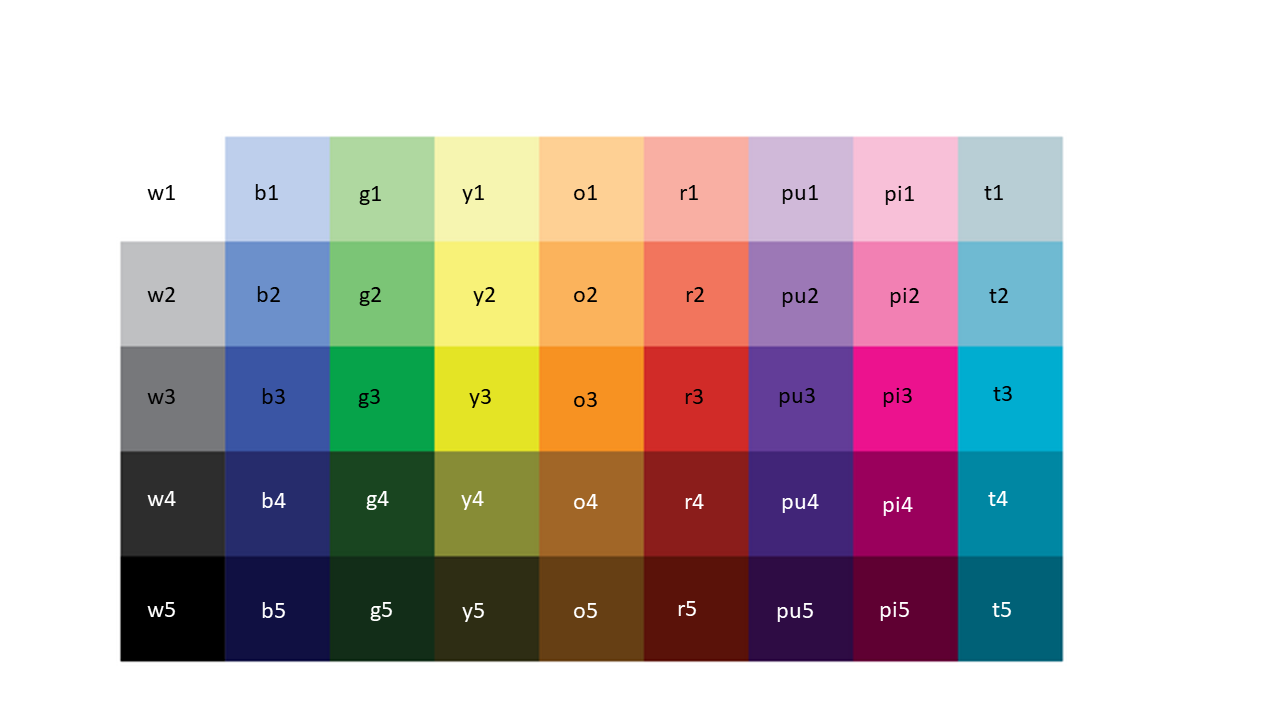


**Figure S1. Color reference chart used to score flower colors from images**. Note that we did not find flowers in each color category. We present the number of species scored into each category for flower colors in Table S3. Next, we used different schemes of categorizing flower colors and assessed how many species were present per category. Finally, to make flower color categories comparable to fruit color categories for comparative analyses of color diversity, we summarized colors into eight categories in the following way: “white”—w1, b1, g1, y1, o1, r1, pu1, pi1; “pink”—pi2, pi3, pi4; “purple”—pu2, pu3, pu4 (including blue [b2, b3] since there were only 4 spp. with these colors); “red”—r2, r3, r4; “orange”—o2, o3, o4; “green”—g2, g3, g4; “yellow”—y2, y3, y4; “dark”—g5, o5, pi5, pu5, r5. Merging very light colors (b1, g1, y1, o1, r1, pu1, pi1, t1) with white is consistent with other studies on color diversity that used categories (i.e., Delmas et al., 2020). The number of species scored for each color are B1, 2; B2, 3; G1, 238; G2, 100; G3, 5; G4, 1; G5, 1; O1, 8; O2, 5; O3, 6; O4, 9; O5, 1; Pi1, 72; Pi2, 78; Pi3, 37; Pi4, 7; Pi5, 2; pu1, 49; pu2, 223; pu3, 5; pu4, 6; pu5, 1; r1, 14; r2, 21; r3, 47; r4, 24; r5, 6; w1, 1383; w2, 1; y1, 296; y2, 129; y3, 41; y4, 5.

**
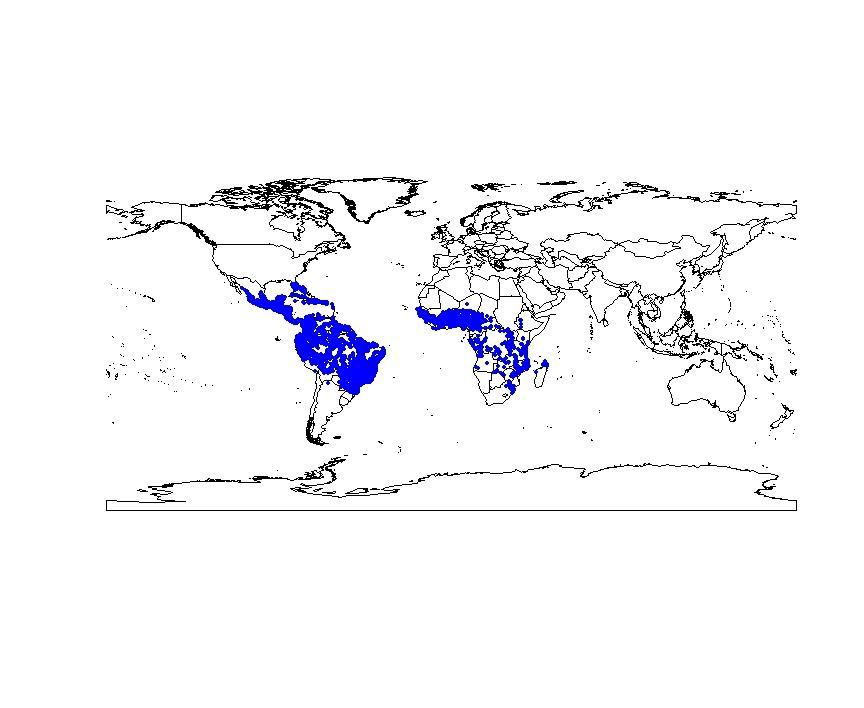
**

***Annona***

**
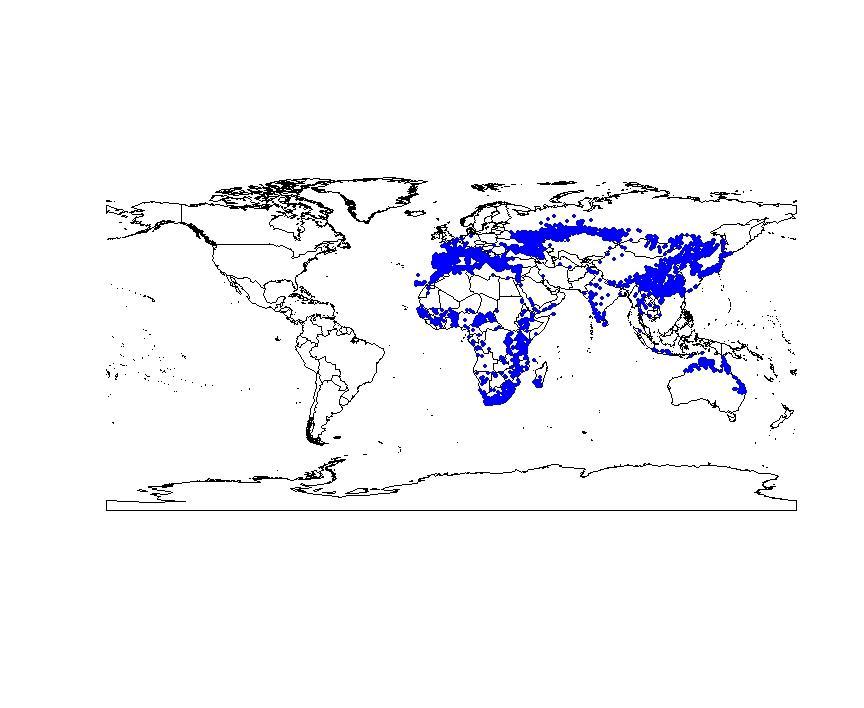
**

***Asparagus***

**
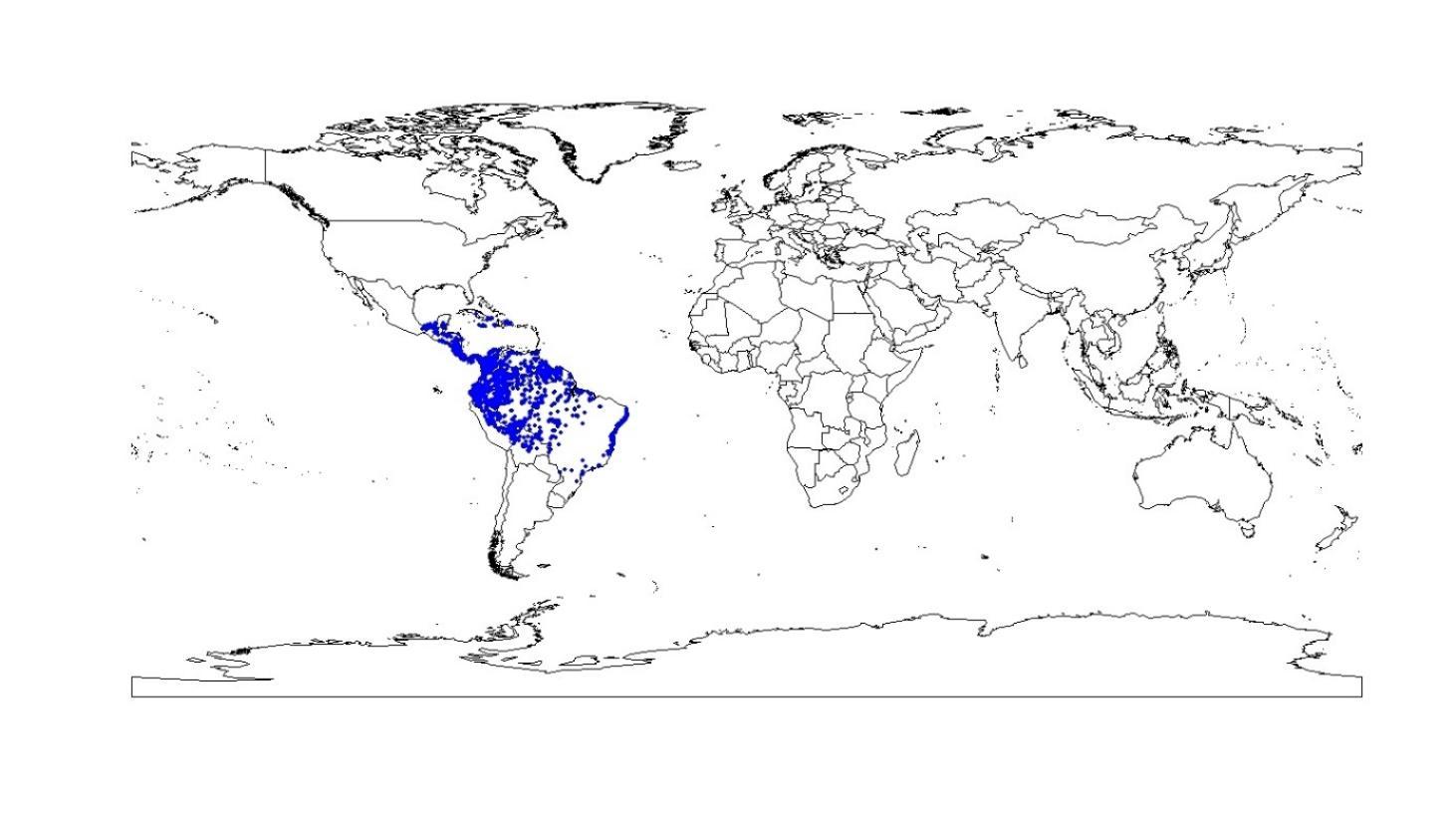
**

***Bactris***

**
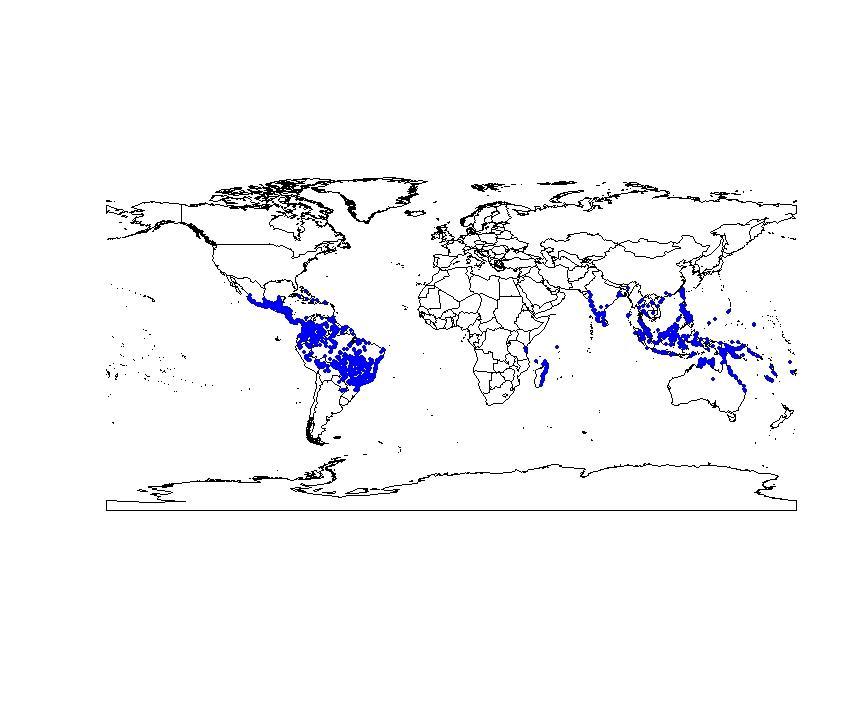
**

***Calophyllum***

**
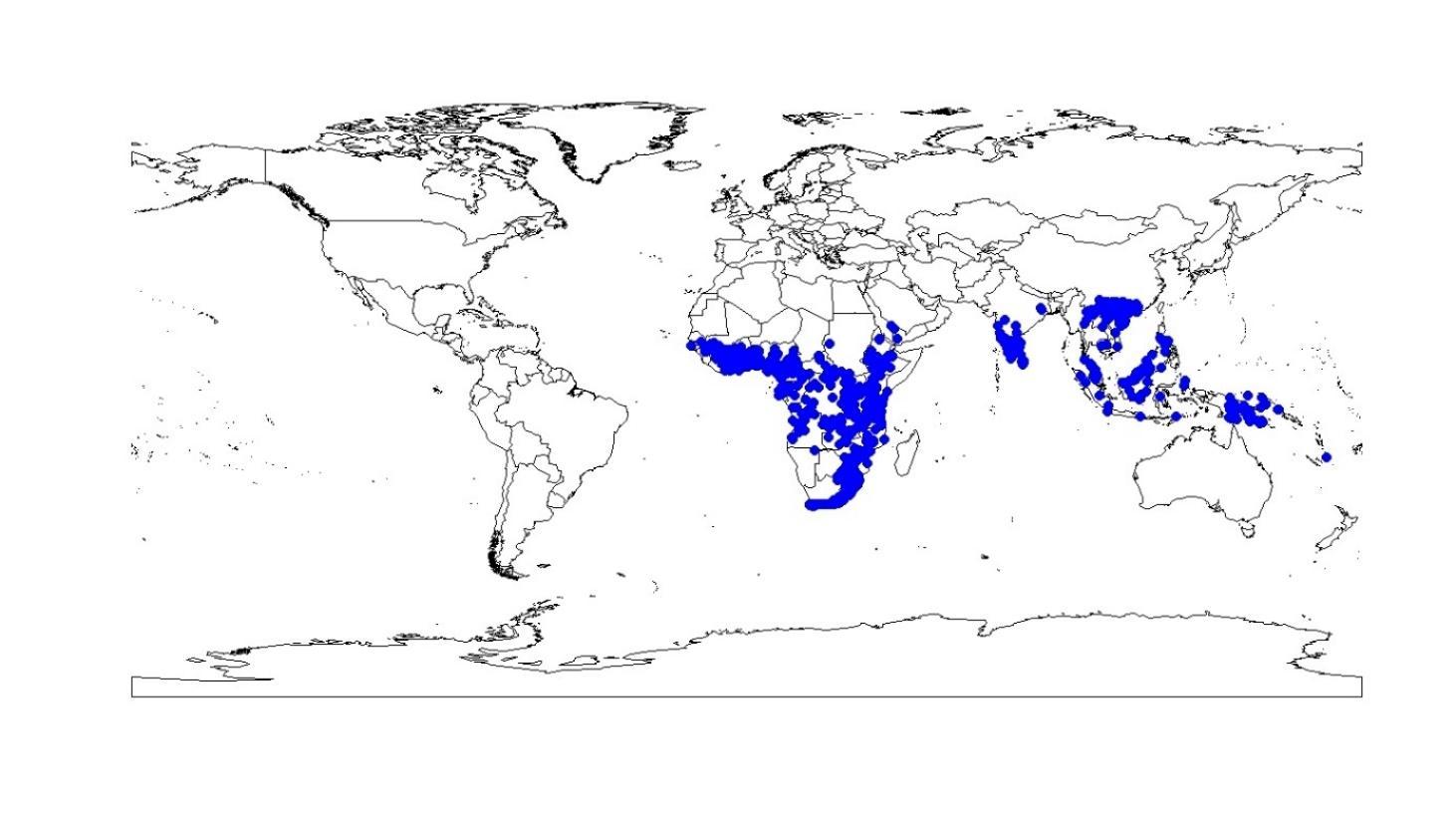
**

***Canthium***

**
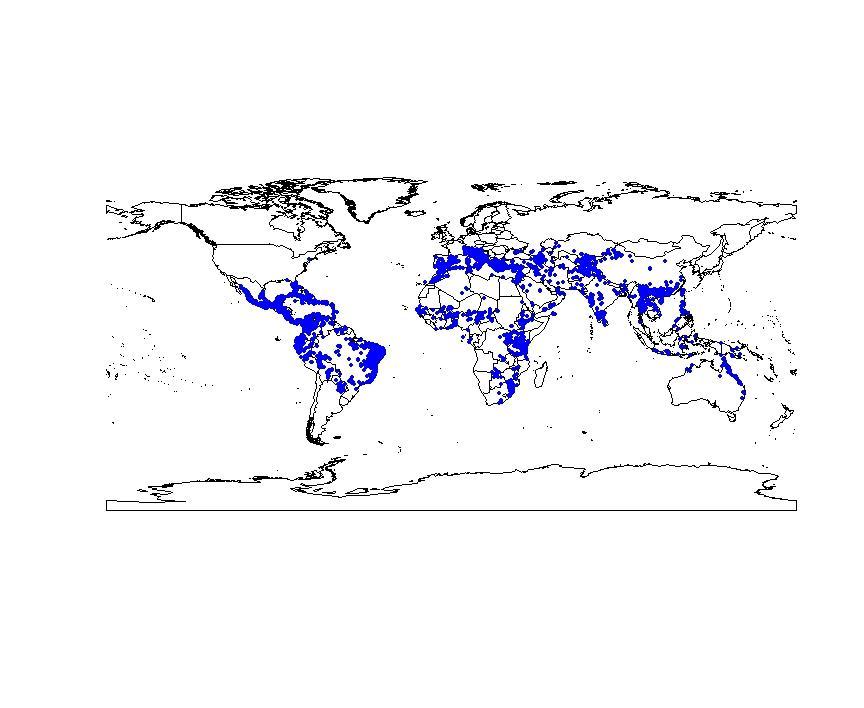
**

***Capparis***

**
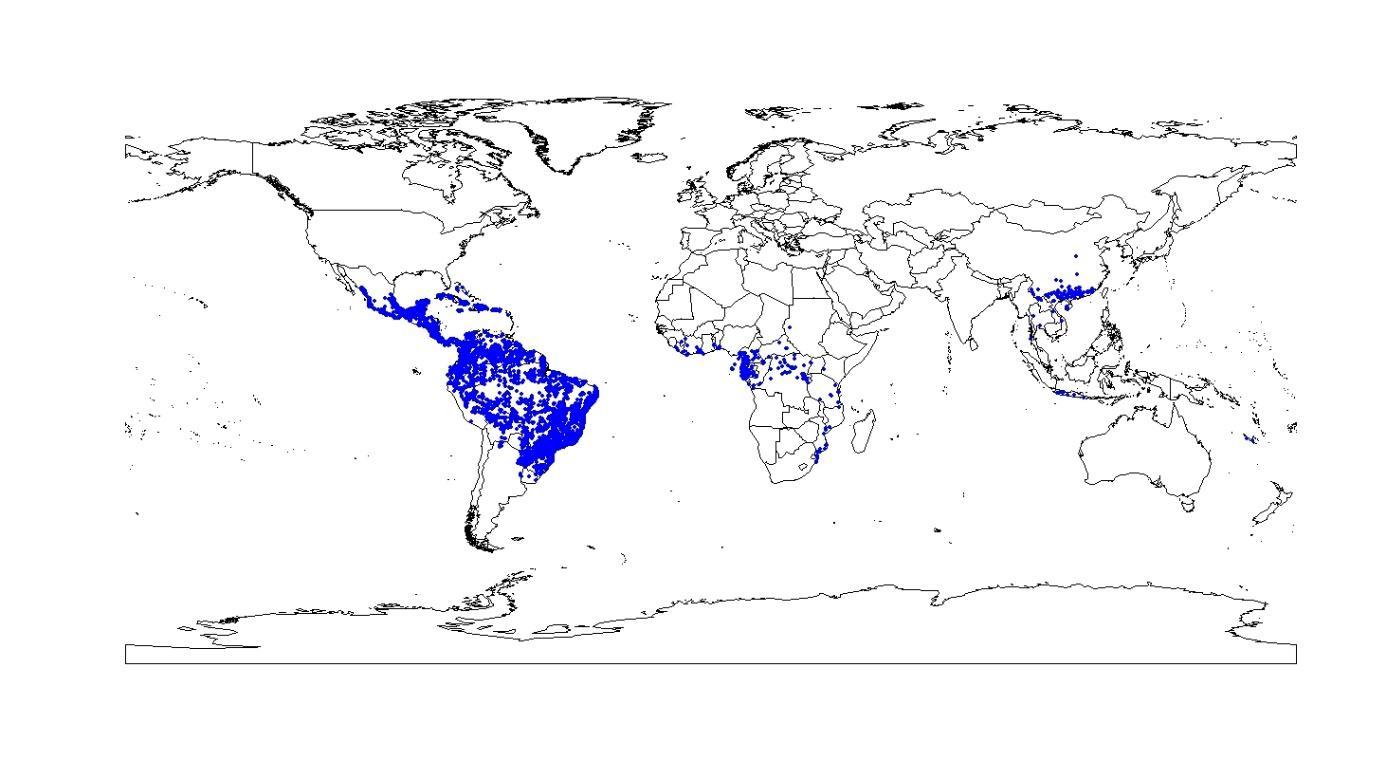
**

***Casearia***

**
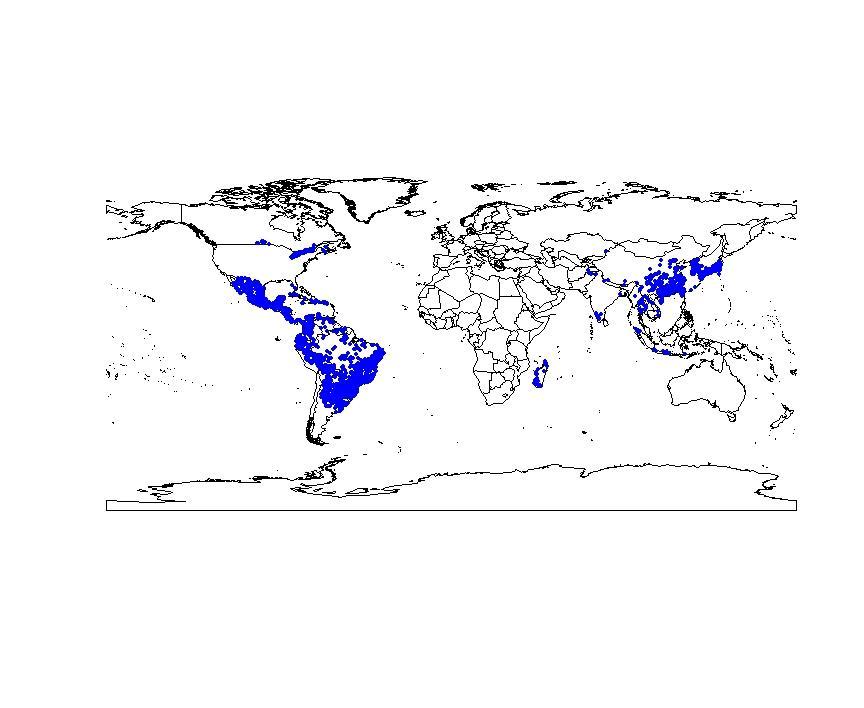
**

***Celtis***

***
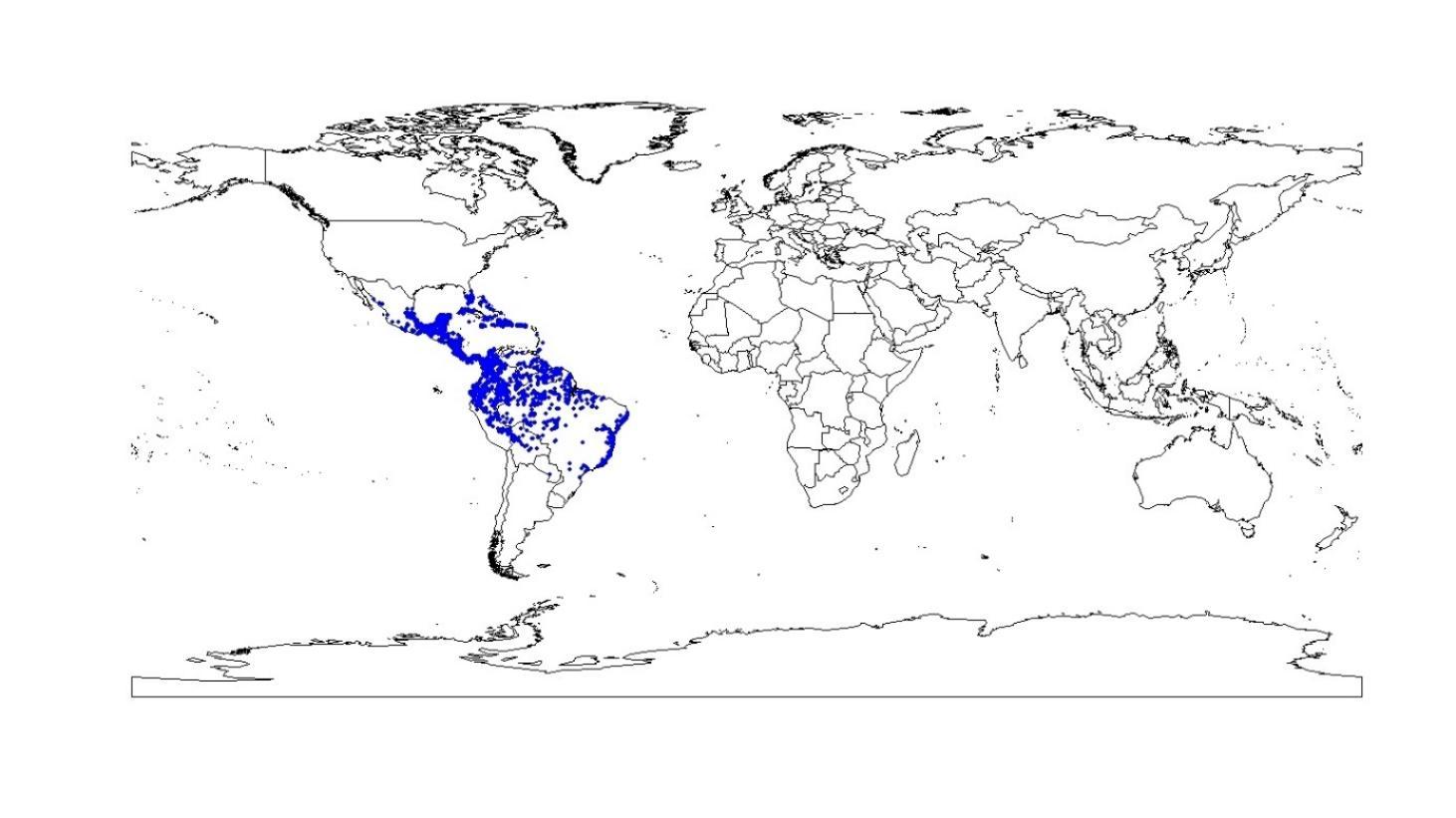
***

***Chrysophyllum***

**
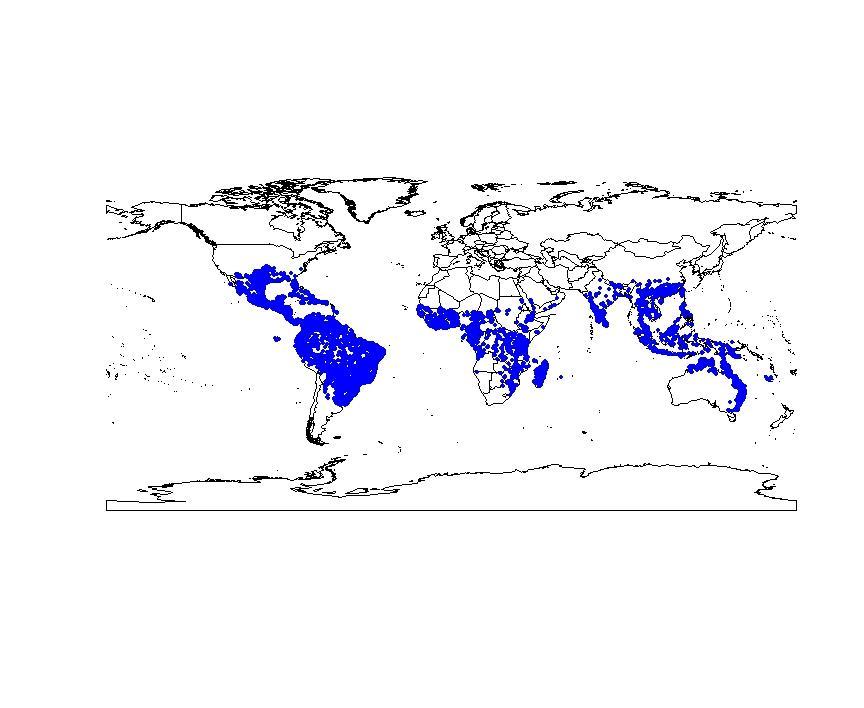
**

***Cissus***

**
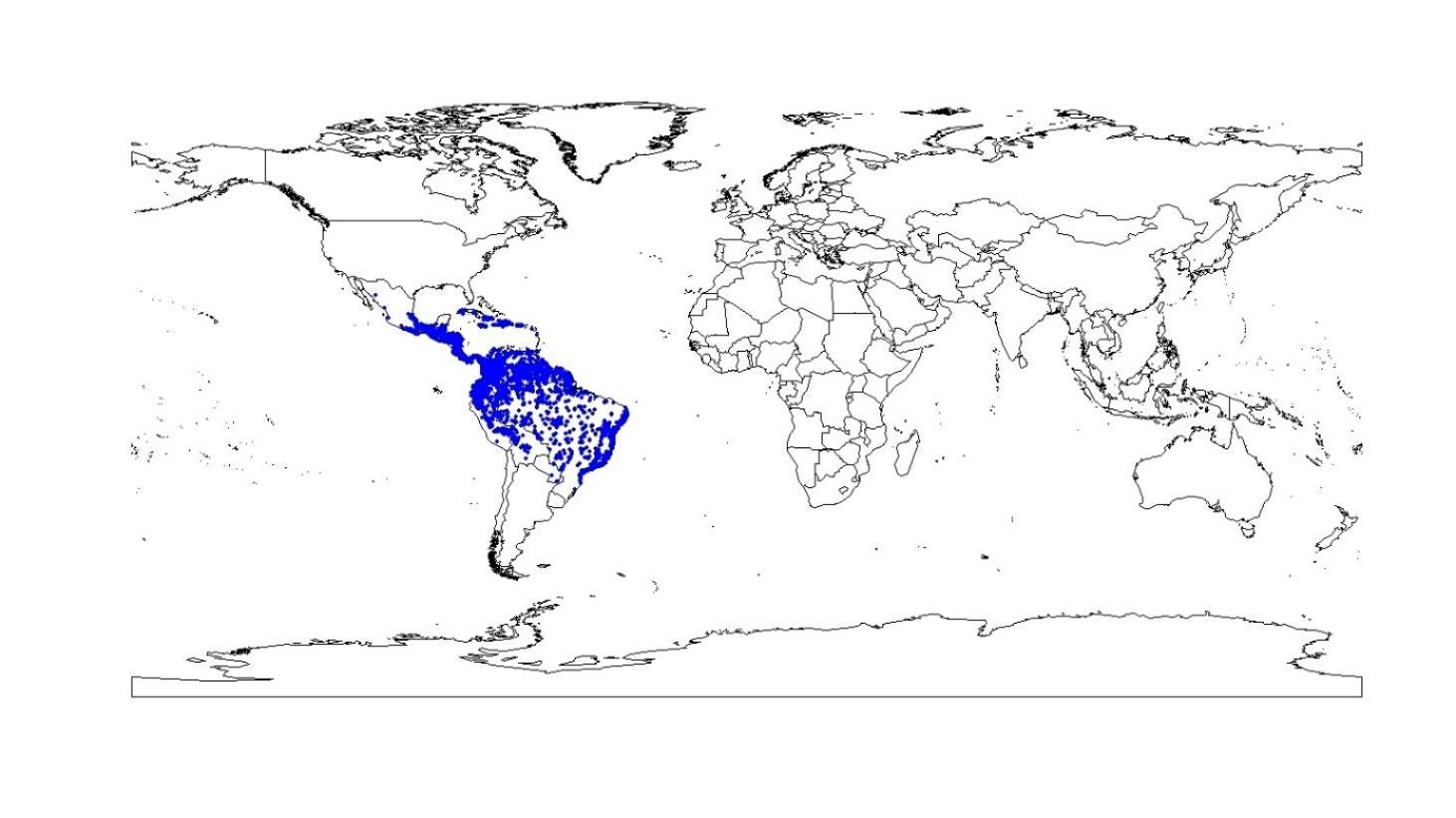
**

***Clidemia***

**
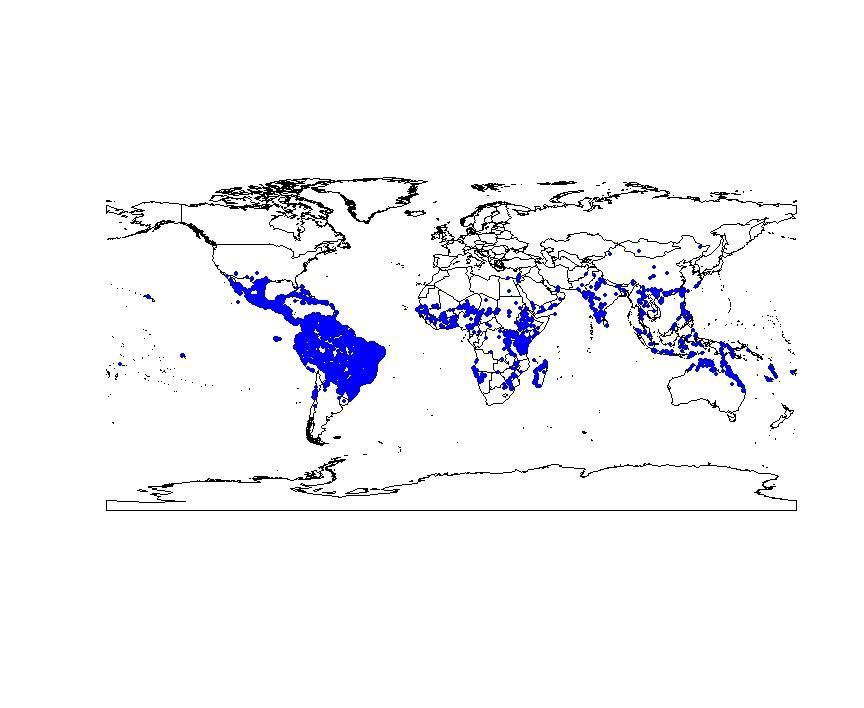
**

***Cordia***

**
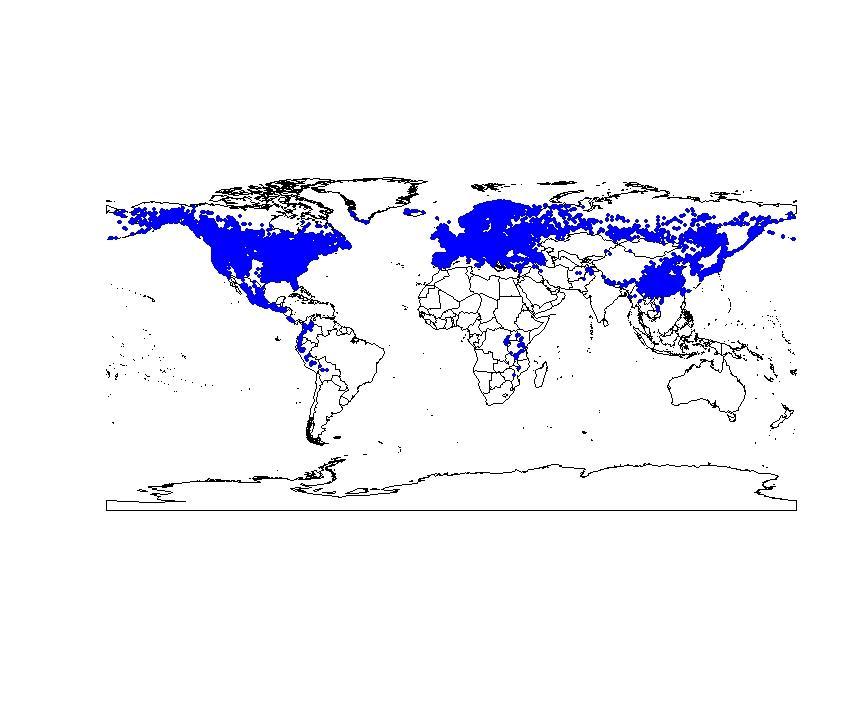
**

***Cornus***

**
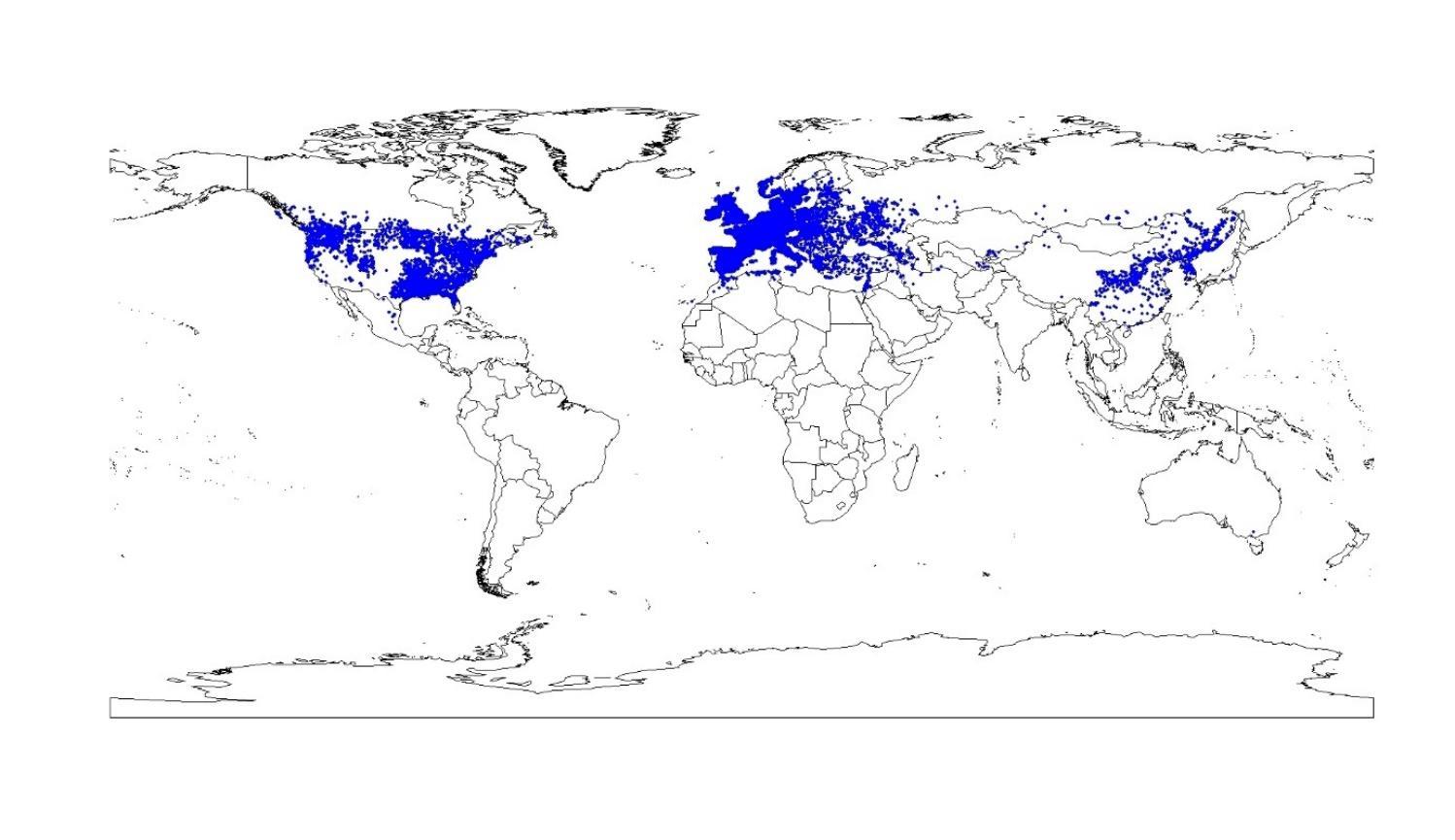
**

***Crataegus***

**
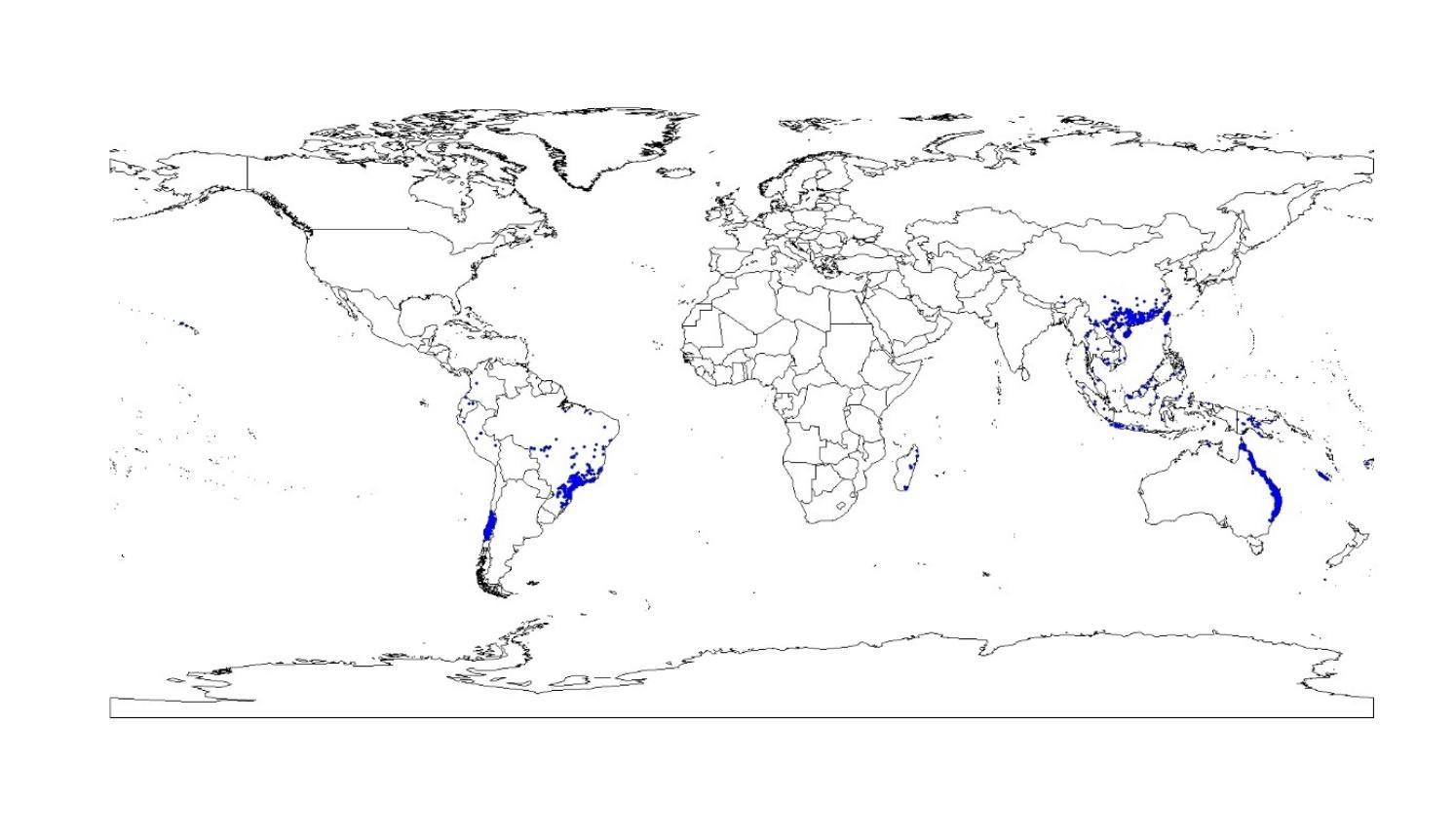
**

***Cryptocarya***

**
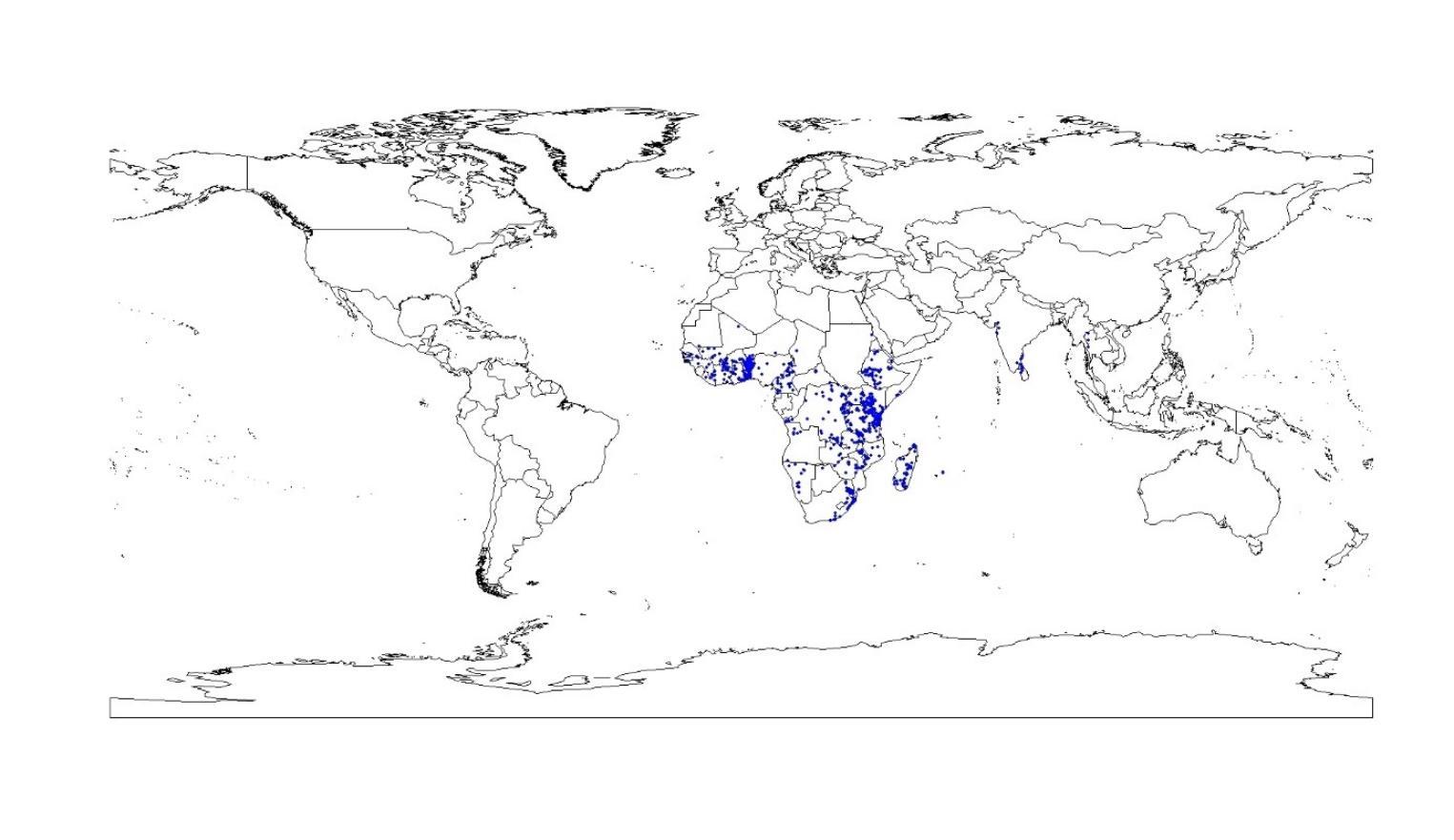
**

***Cyphostemma***

**
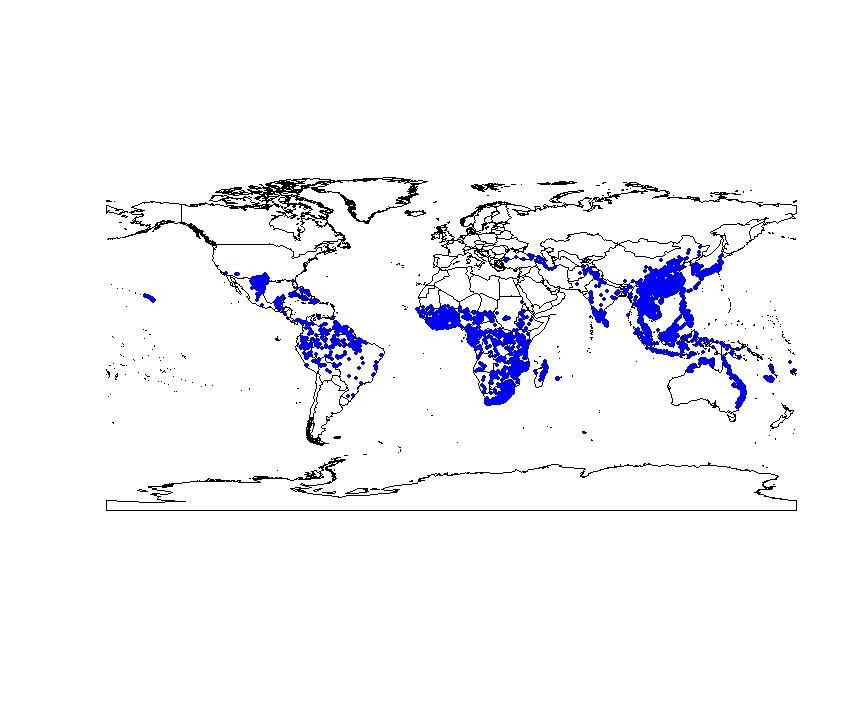
**

***Diospyros***

**
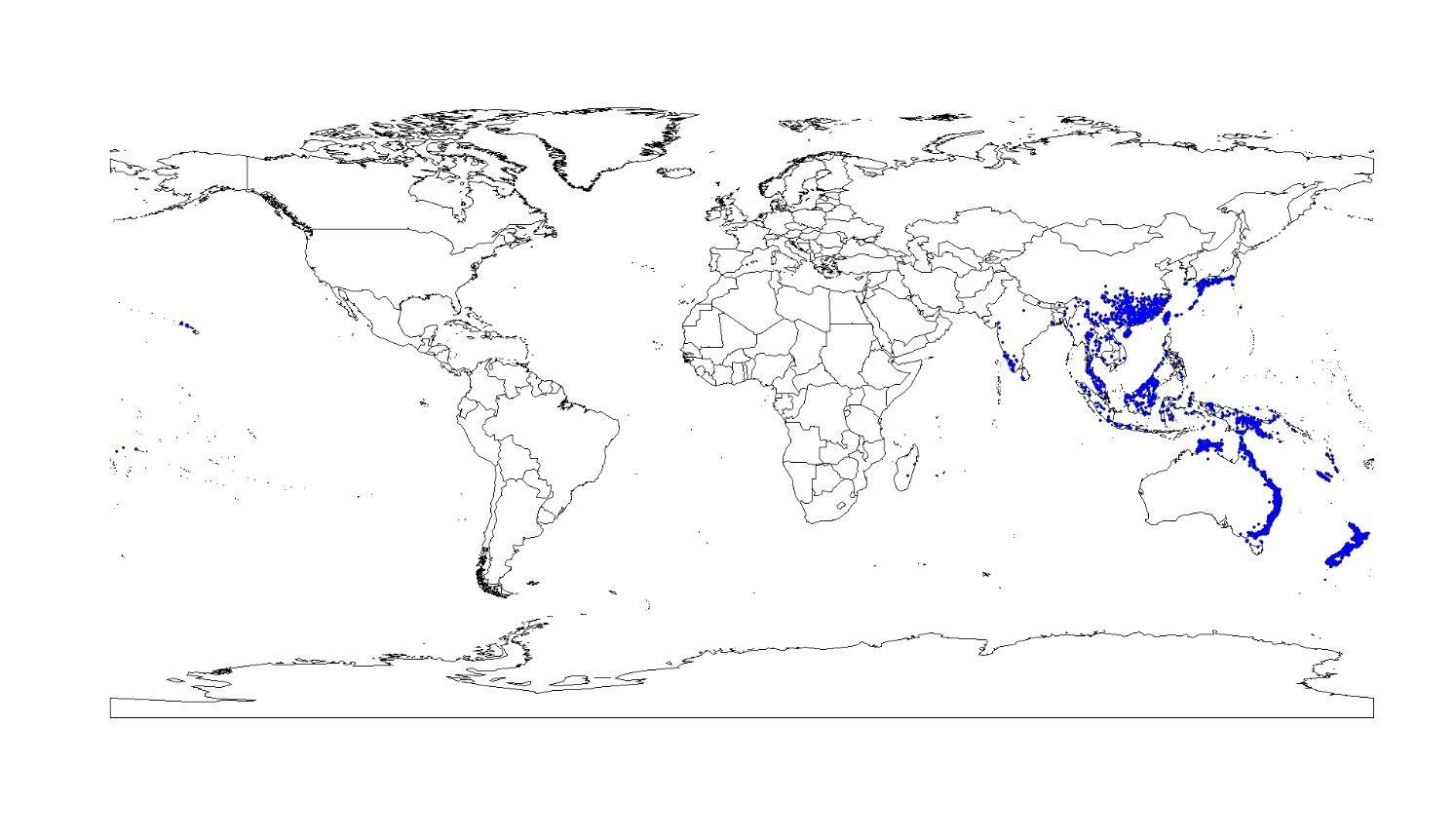
**

***Elaeocarpus***

**
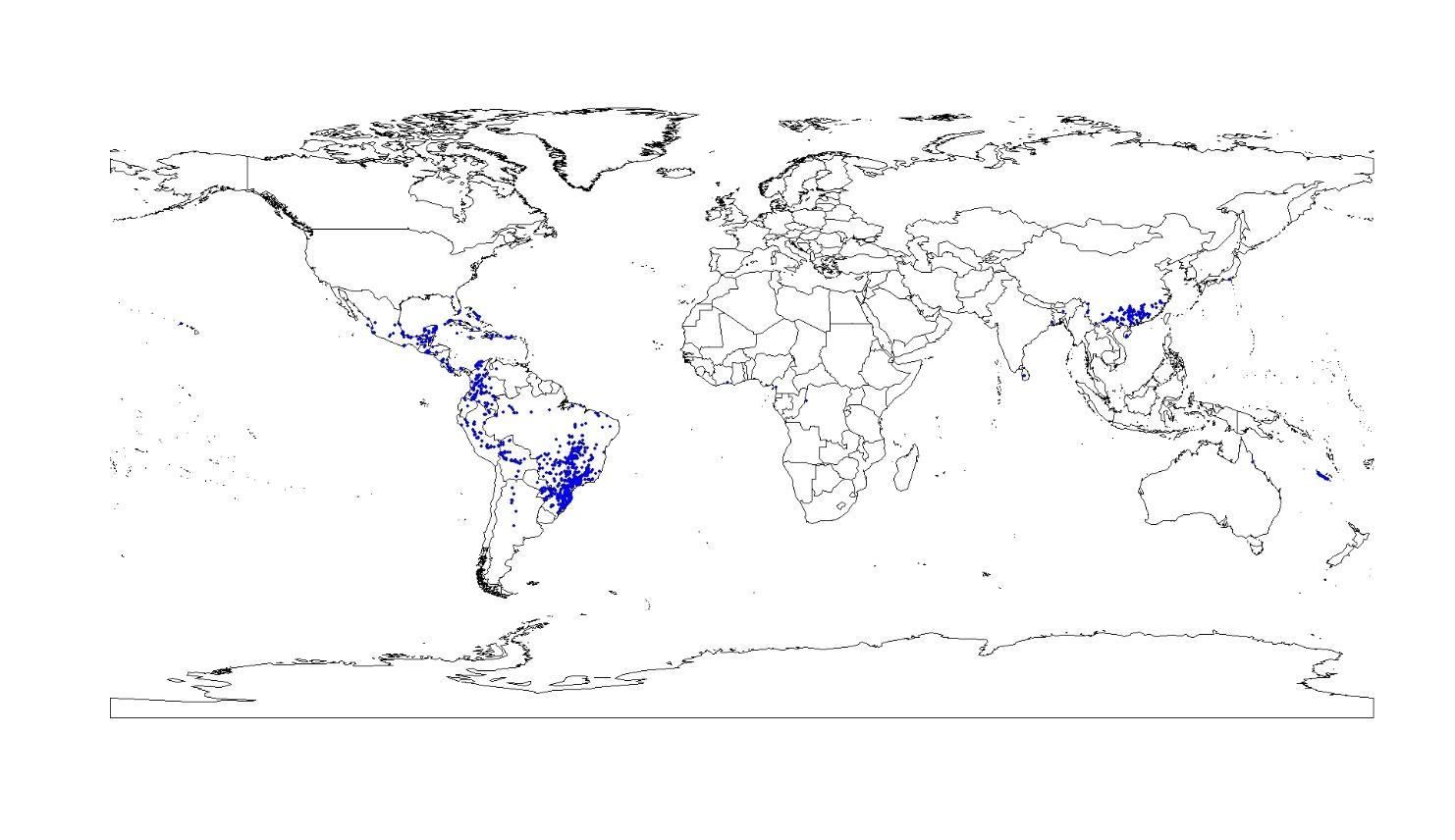
**

***Erythroxylum***

**
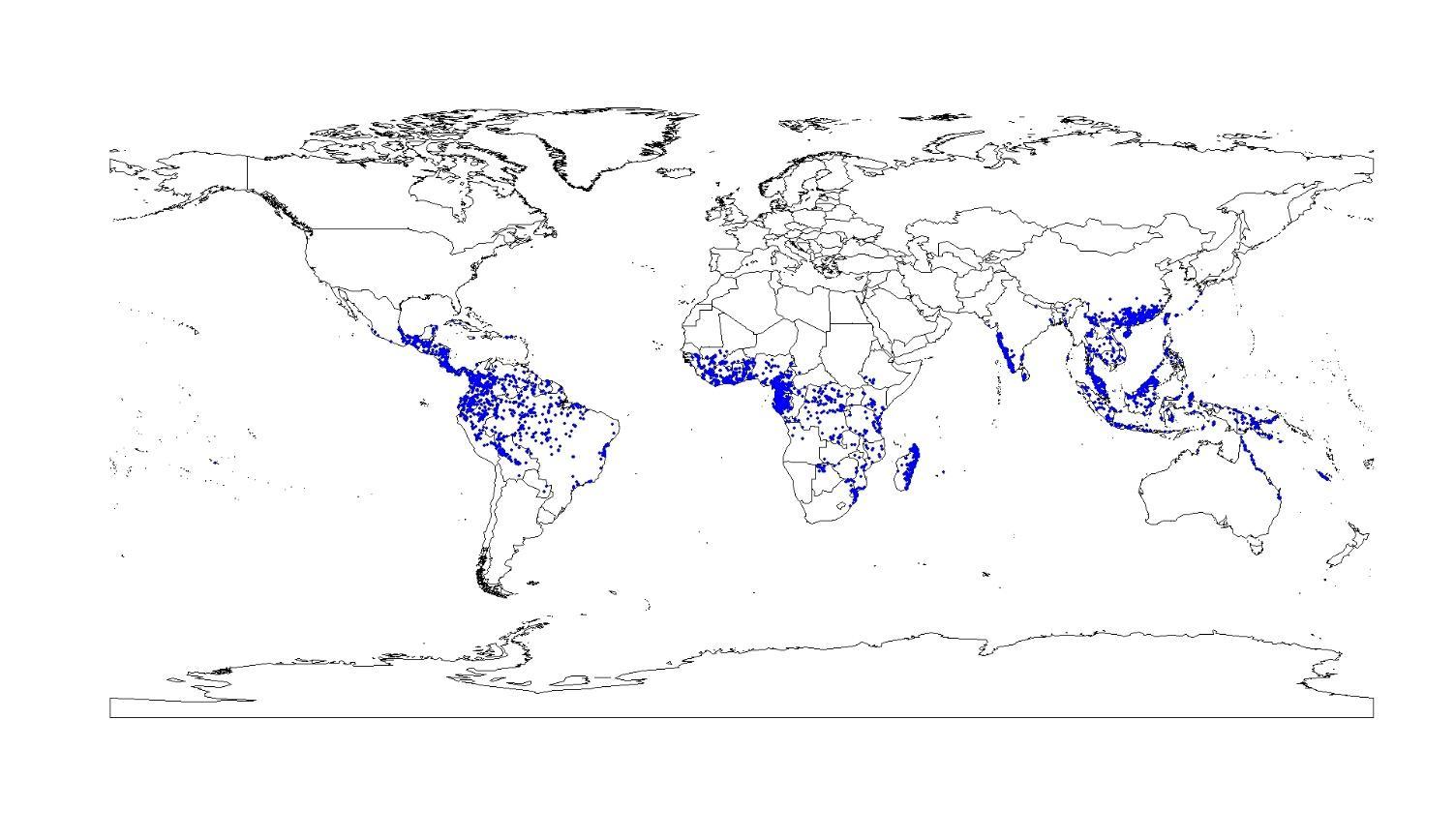
**

***Garcinia***

**
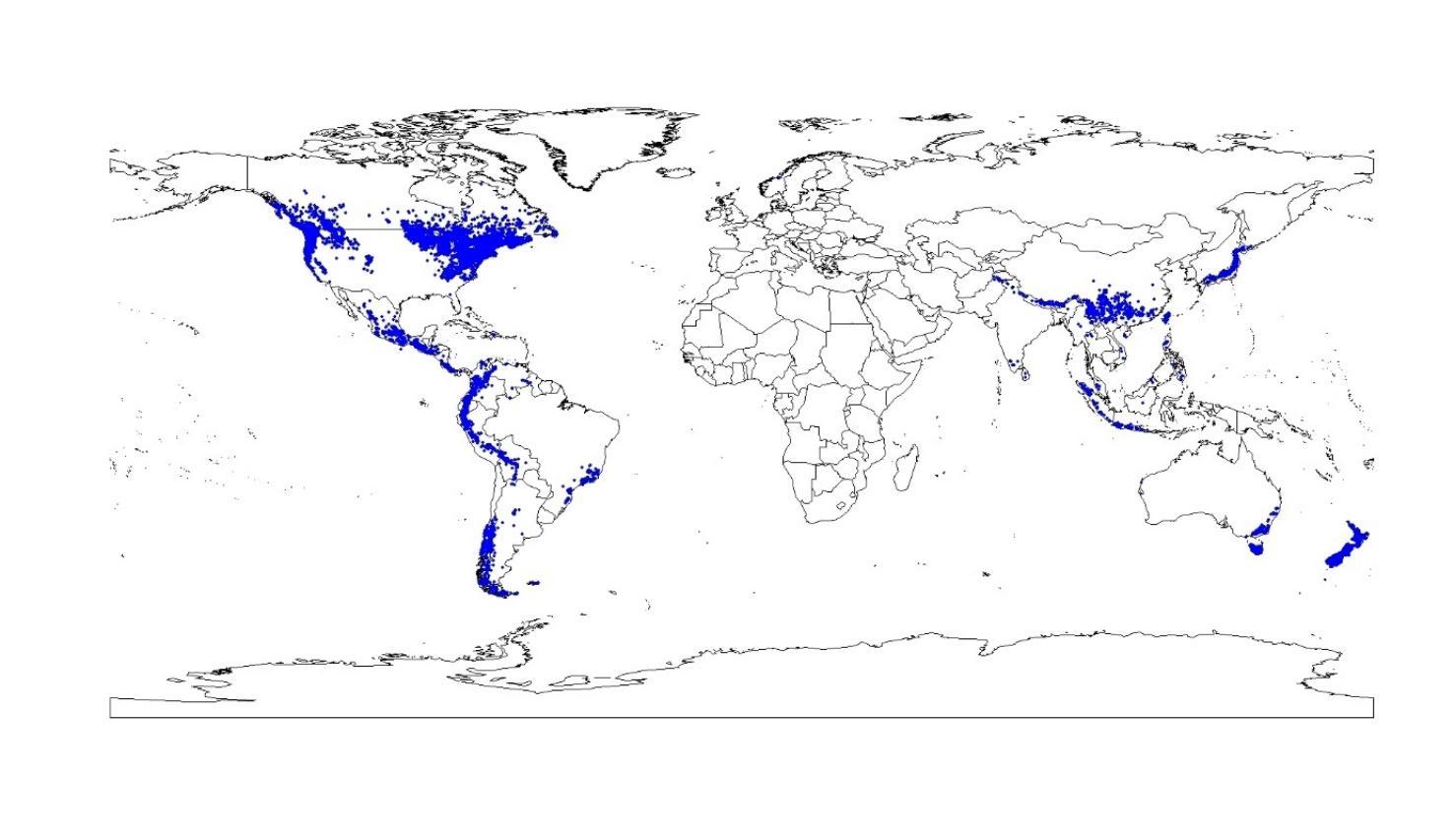
**

***Gaultheria***

**
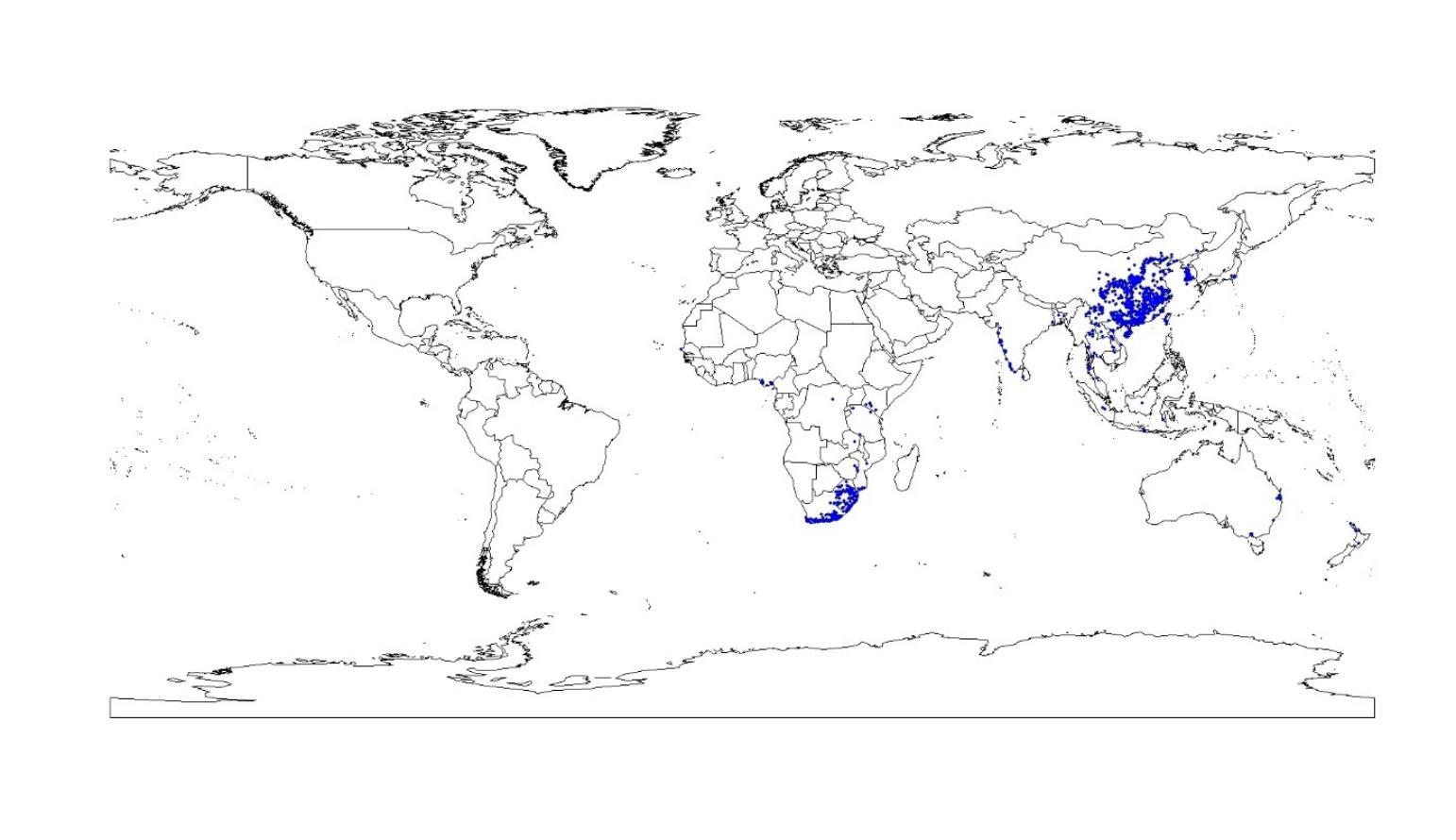
**

***Grewia***

**
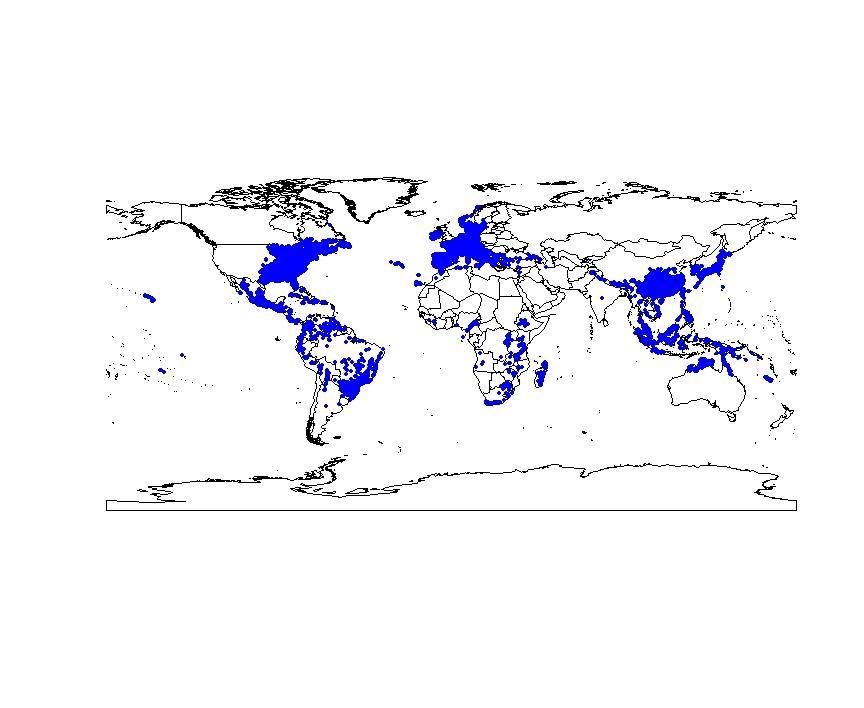
**

***Ilex***

***
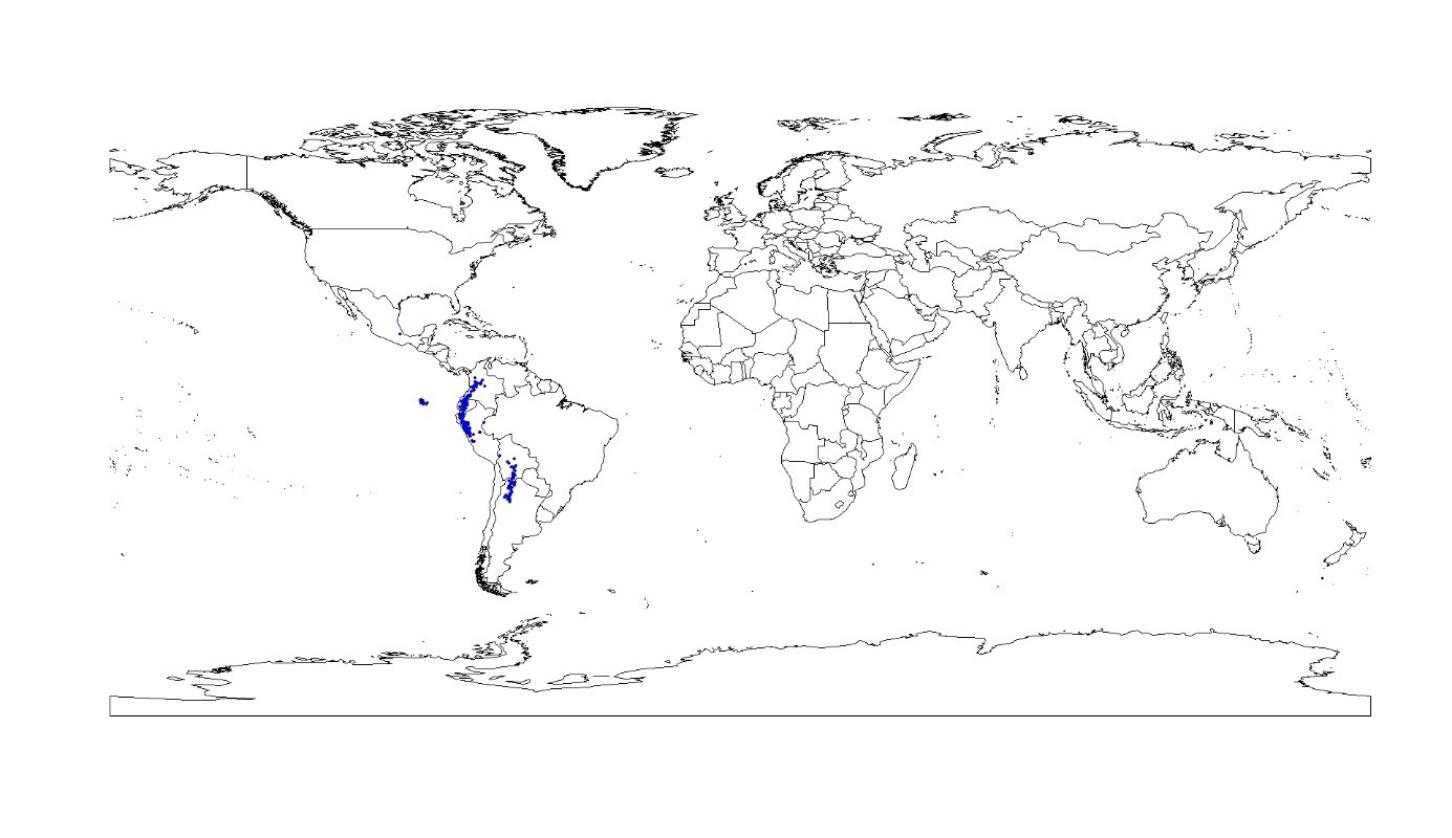
***

***Iochroma***

***
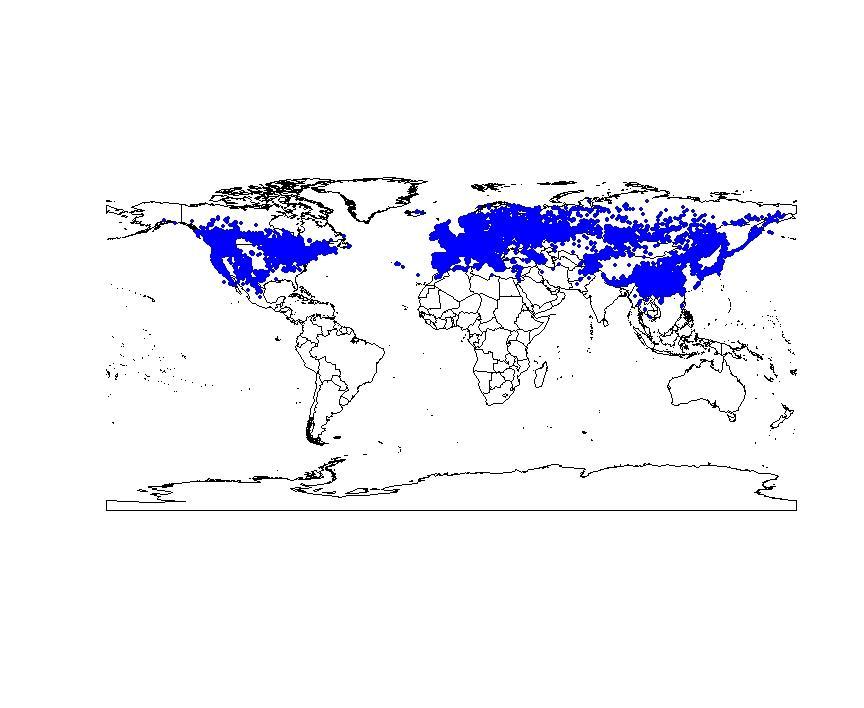
***

***Lonicera***

***
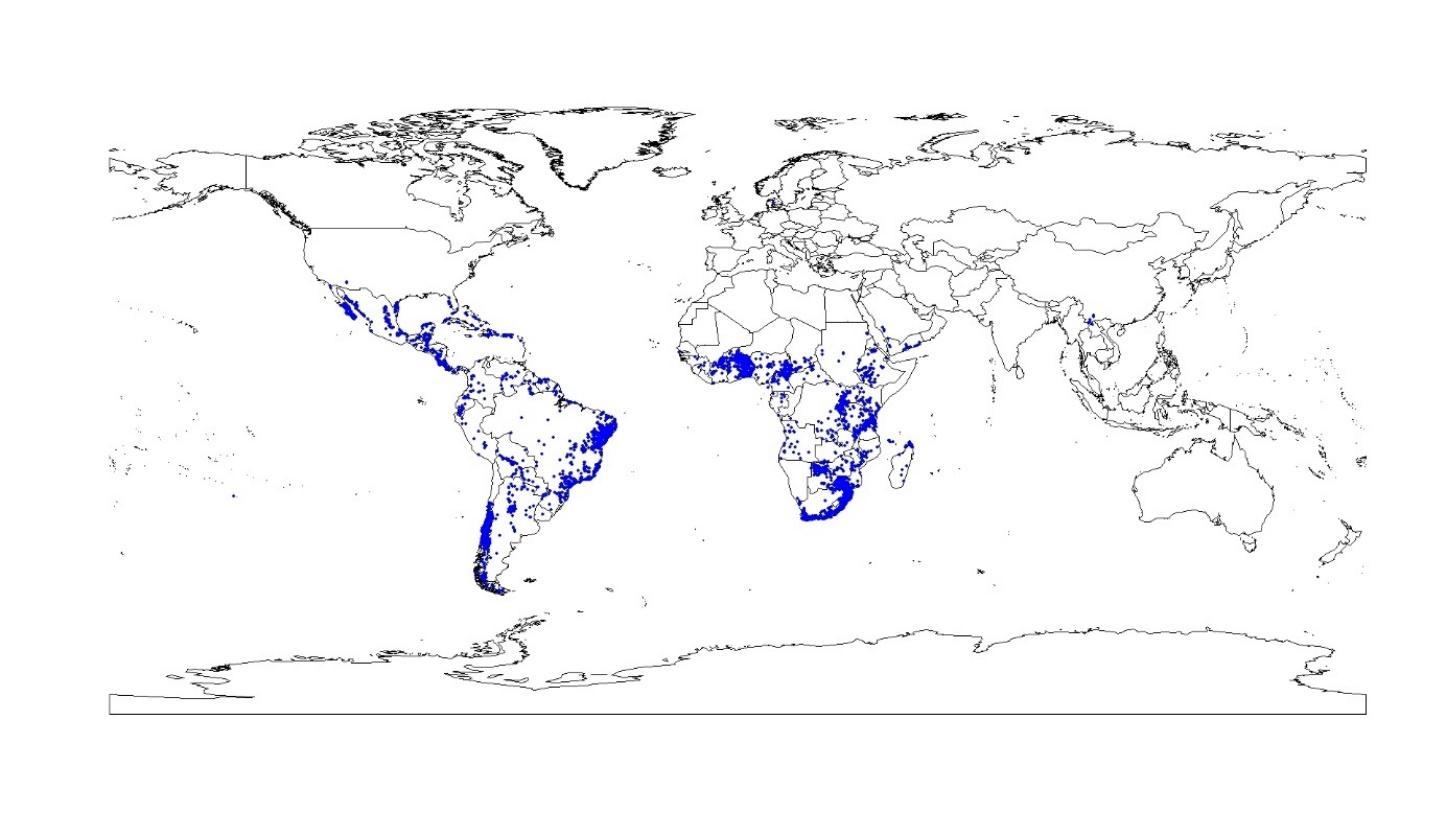
***

***Maytenus***

***
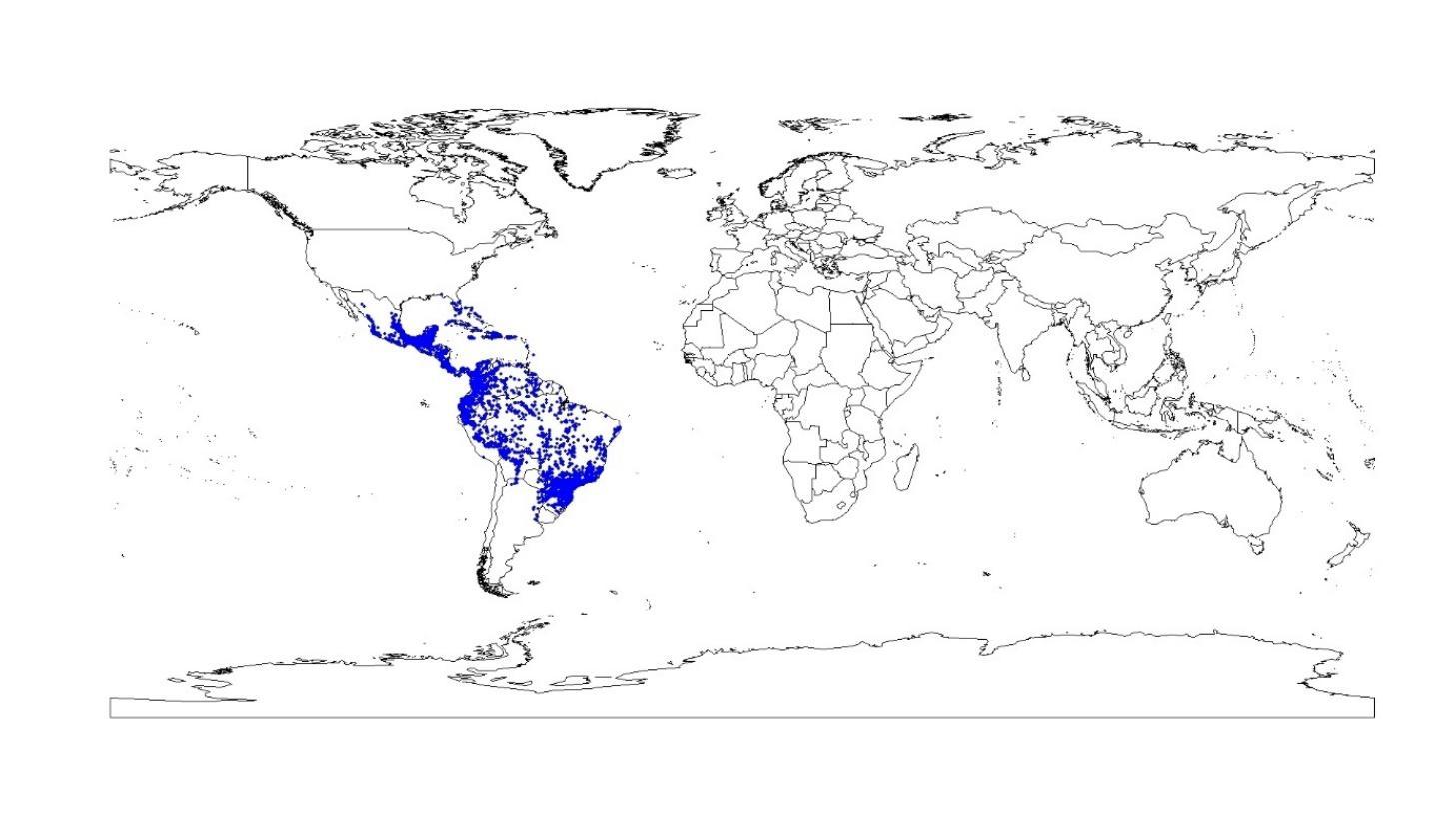
***

***Nectandra***

***
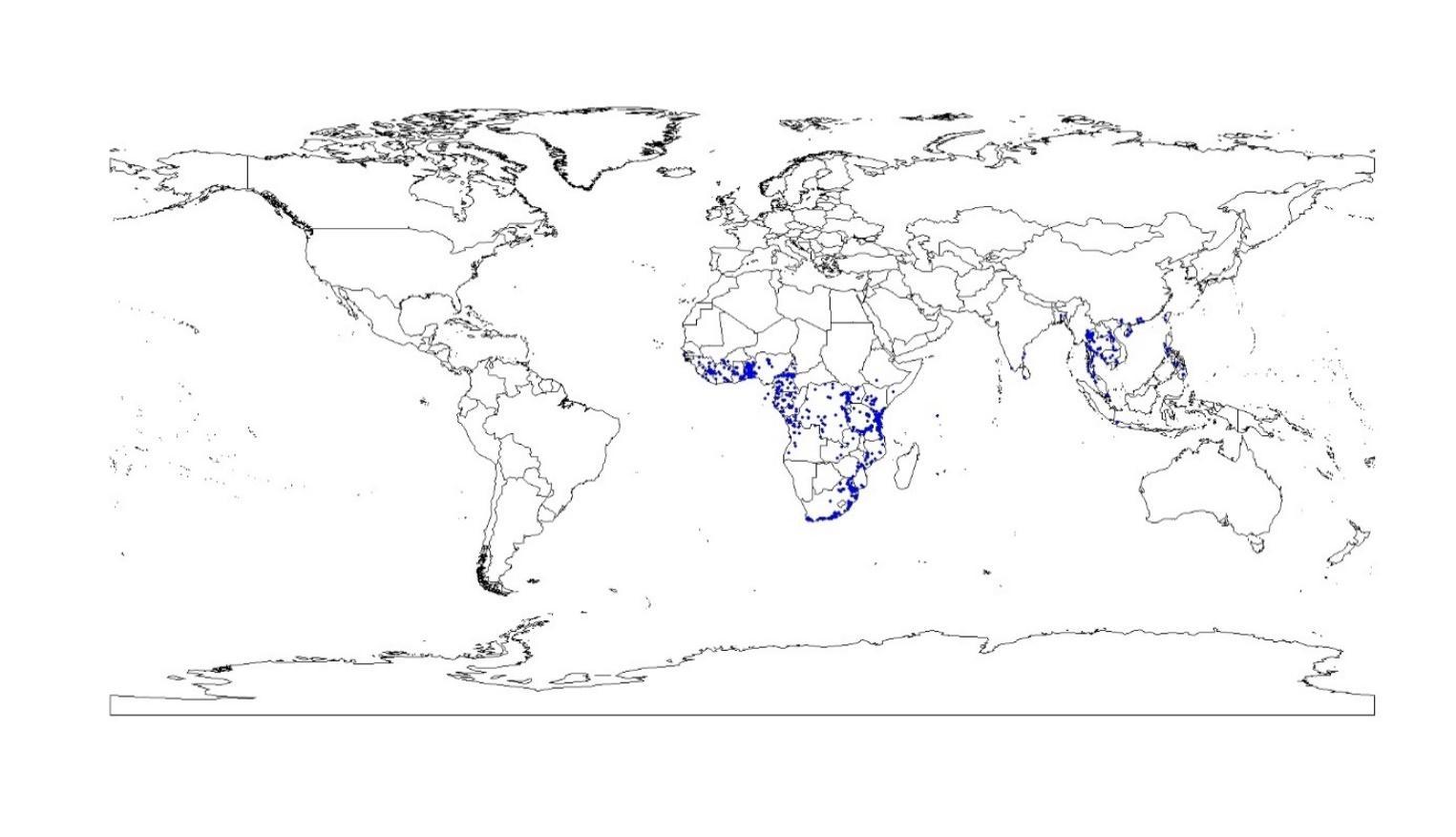
***

***Ochna***

***
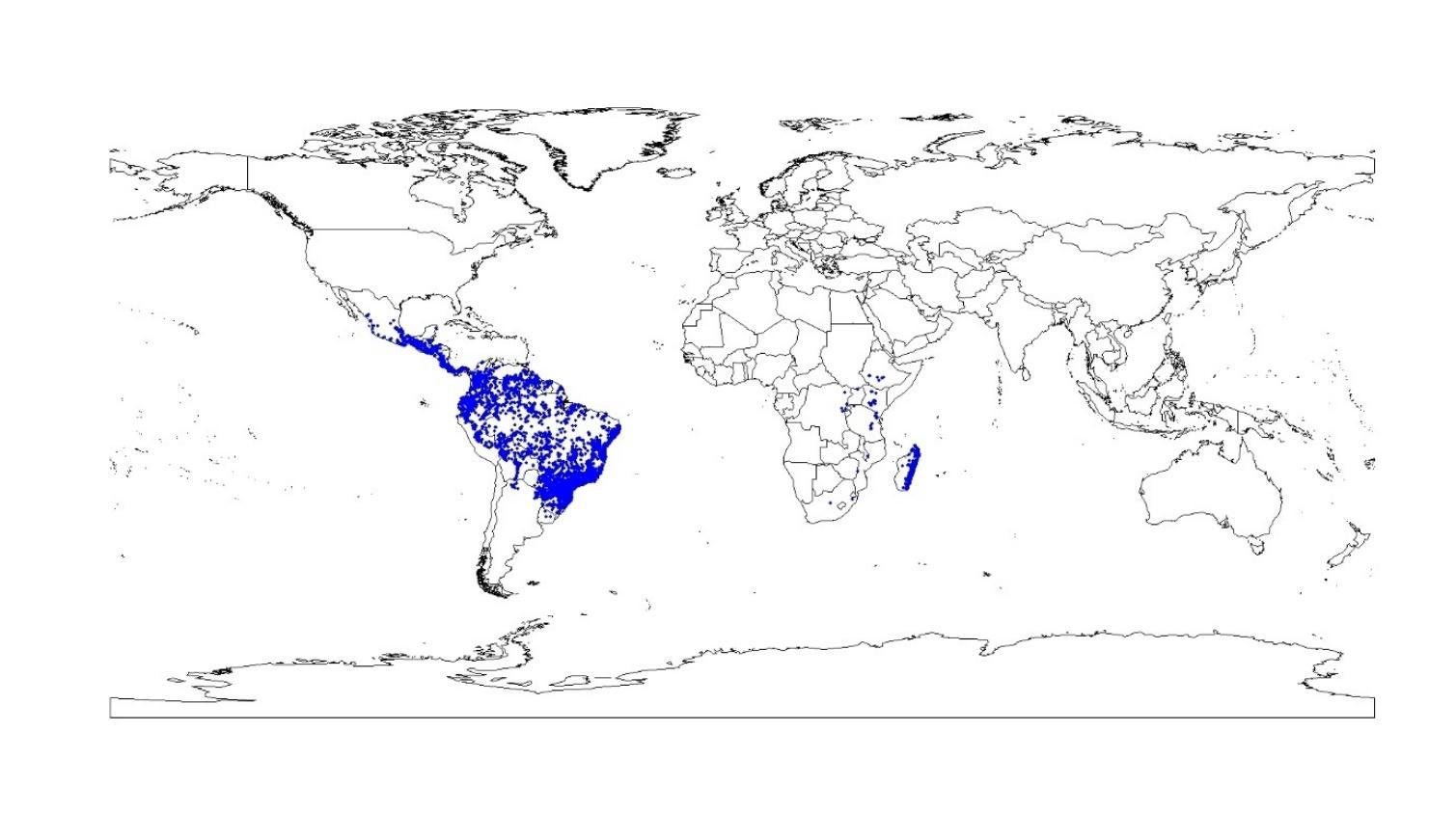
***

***Ocotea***

***
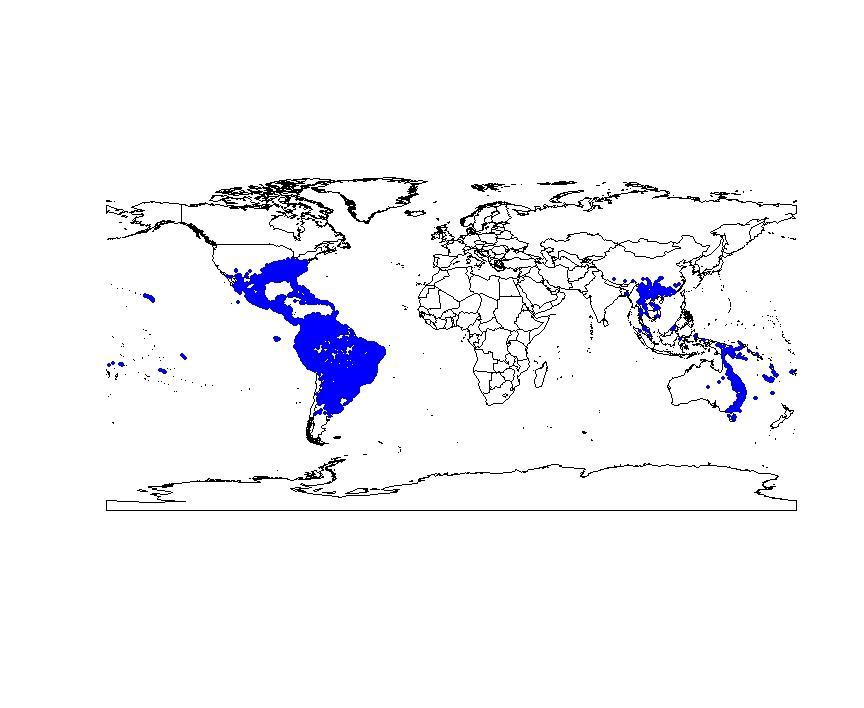
***

***Passiflora***

***
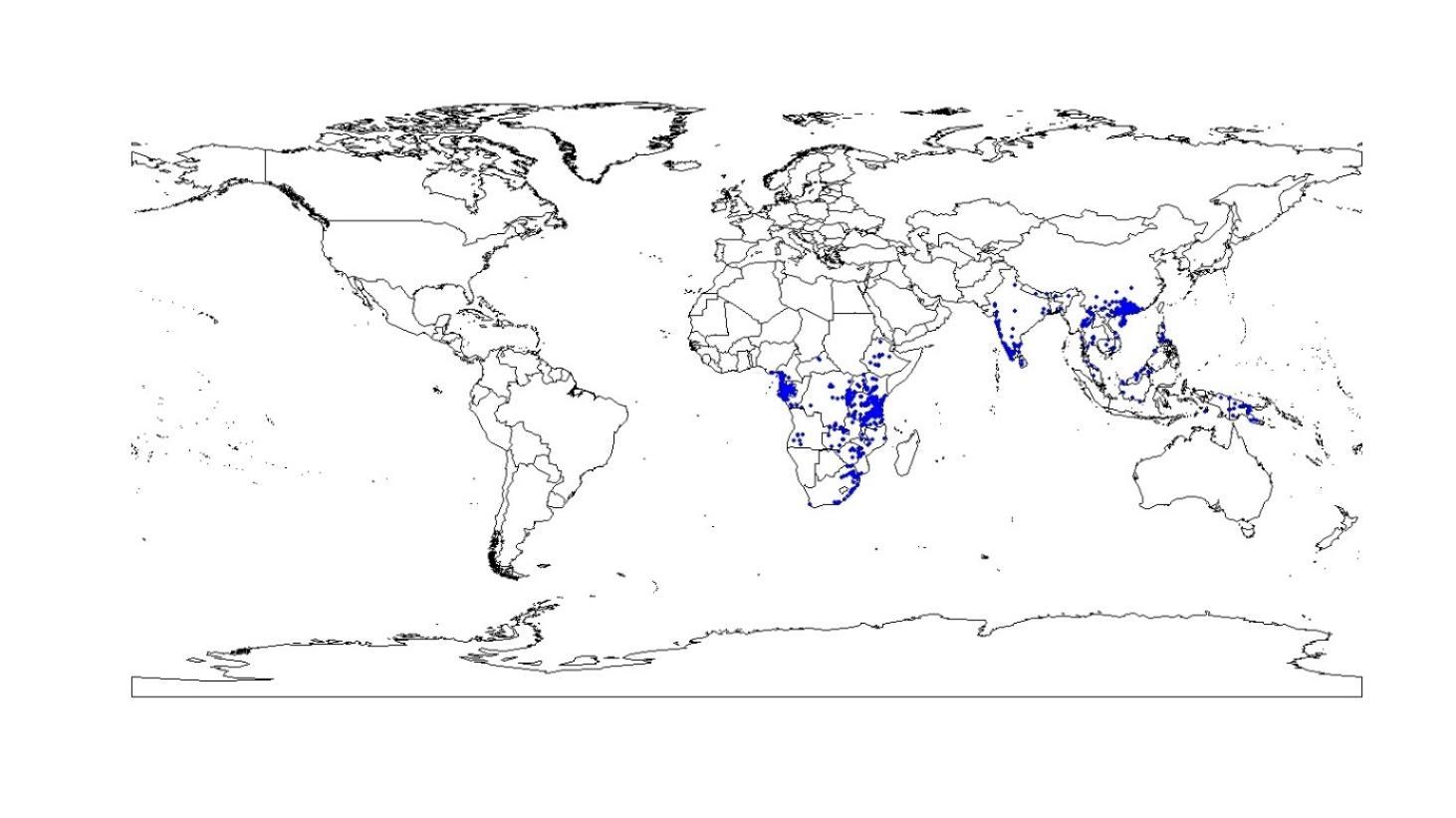
***

***Pavetta***

***
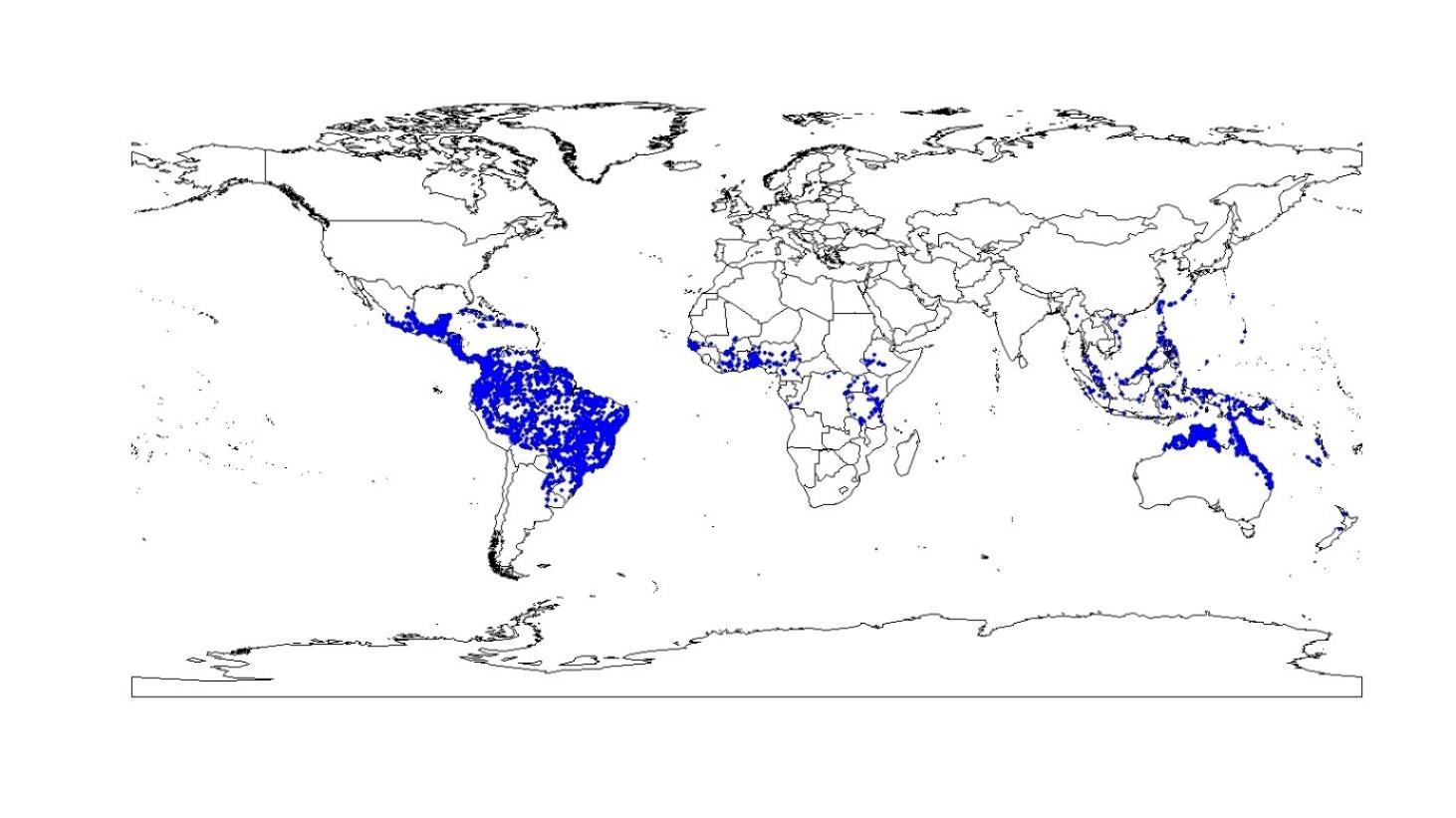
***

***Pouteria***

***
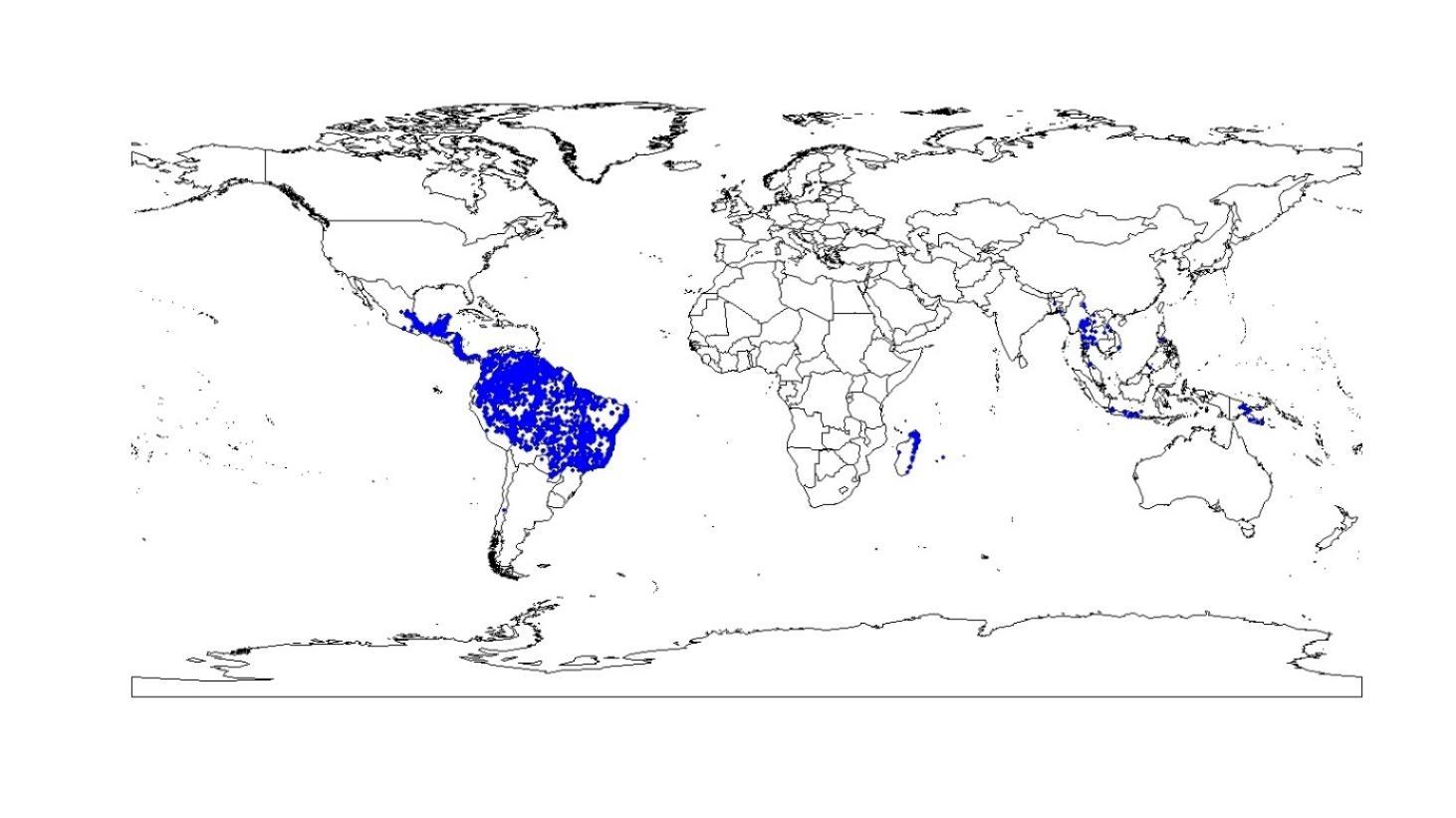
***

***Protium***

***
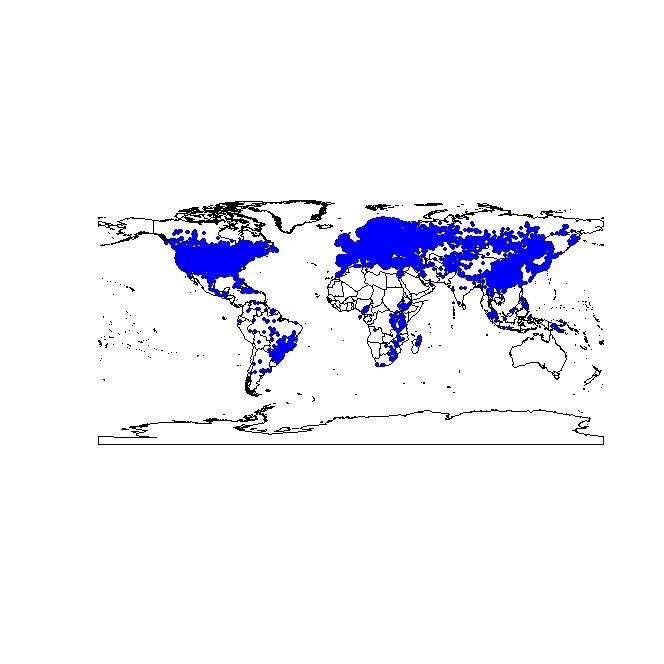
***

***Prunus***

***
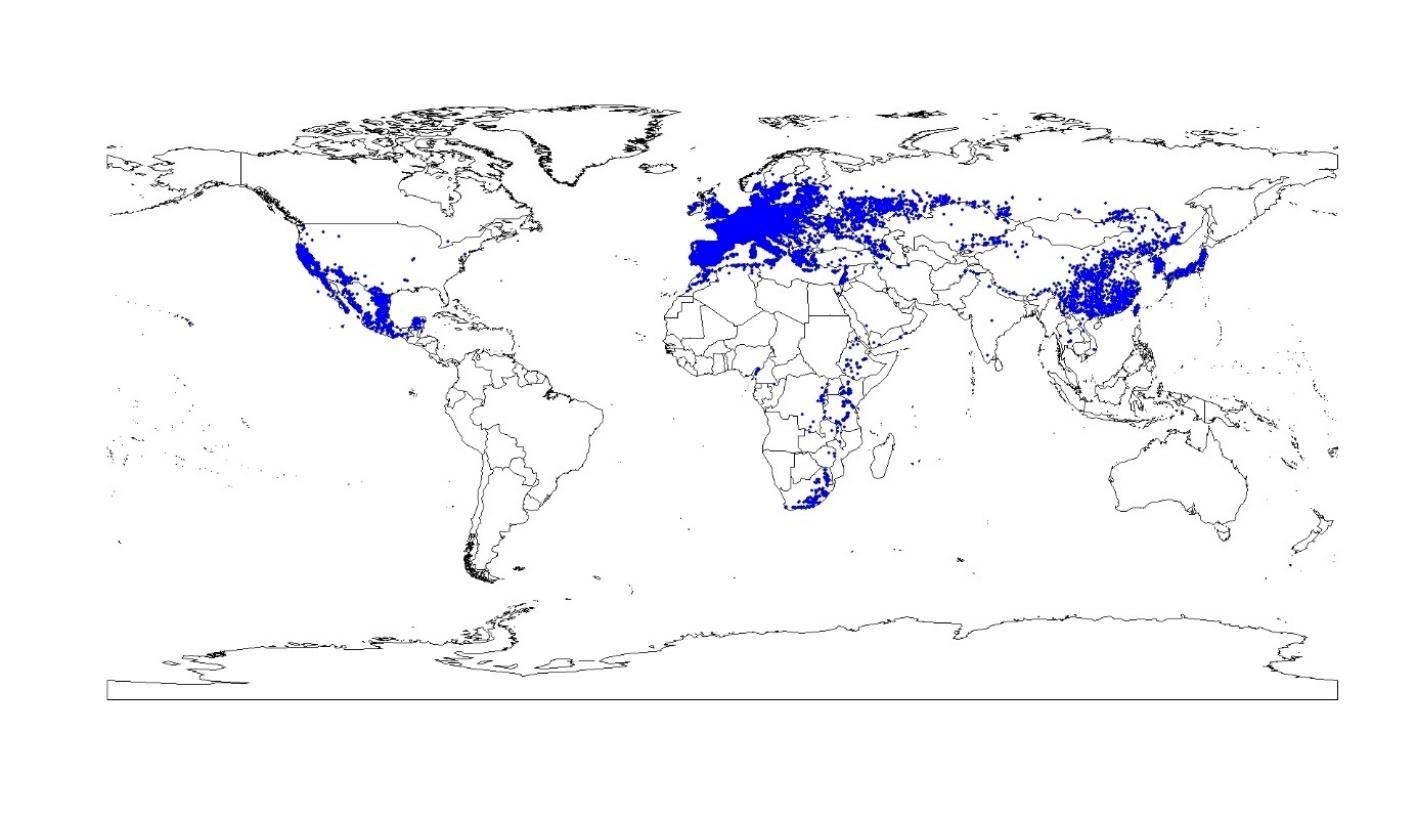
***

***Rhamnus***

***
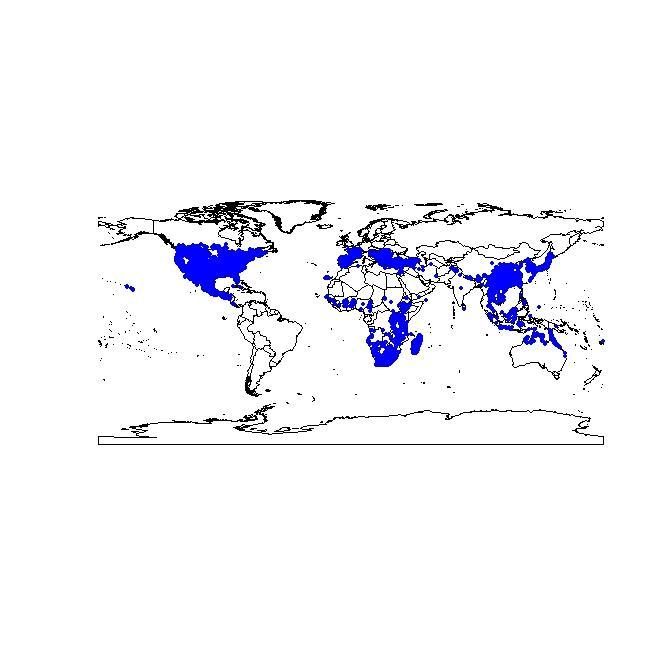
***

***Rhus***

***
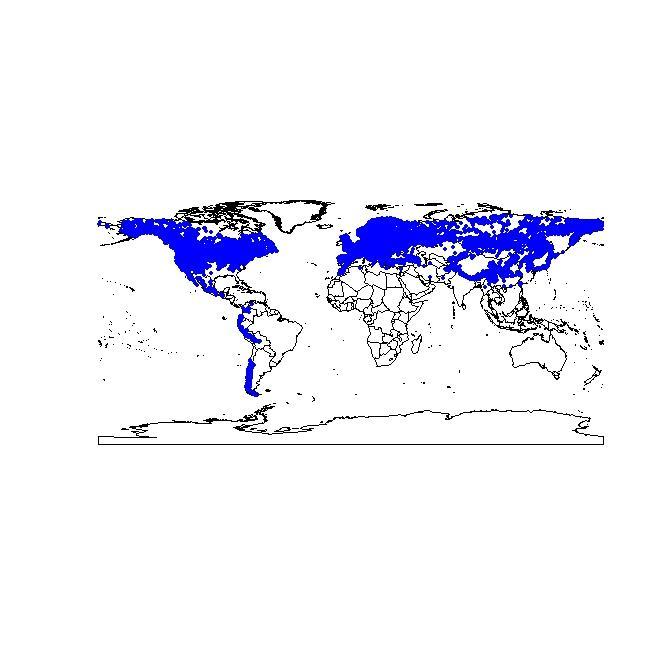
***

***Ribes***

***
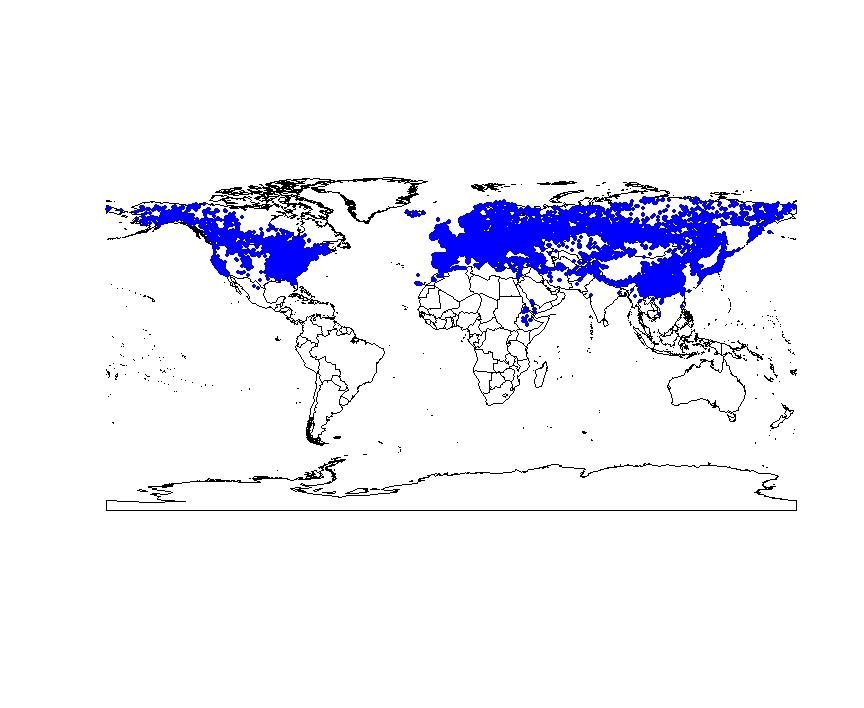
***

***Rosa***

***
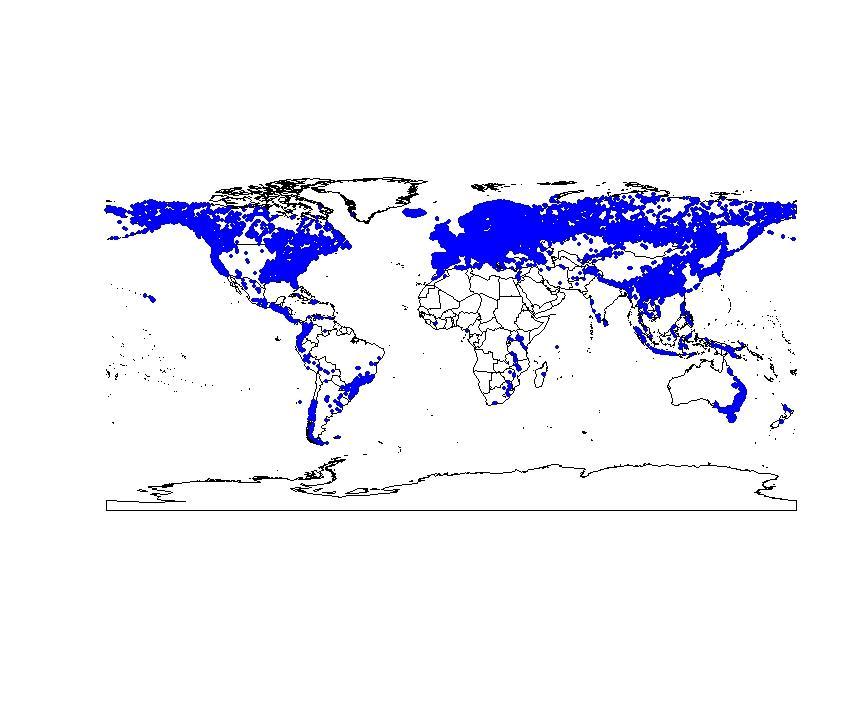
***

***Rubus***

***
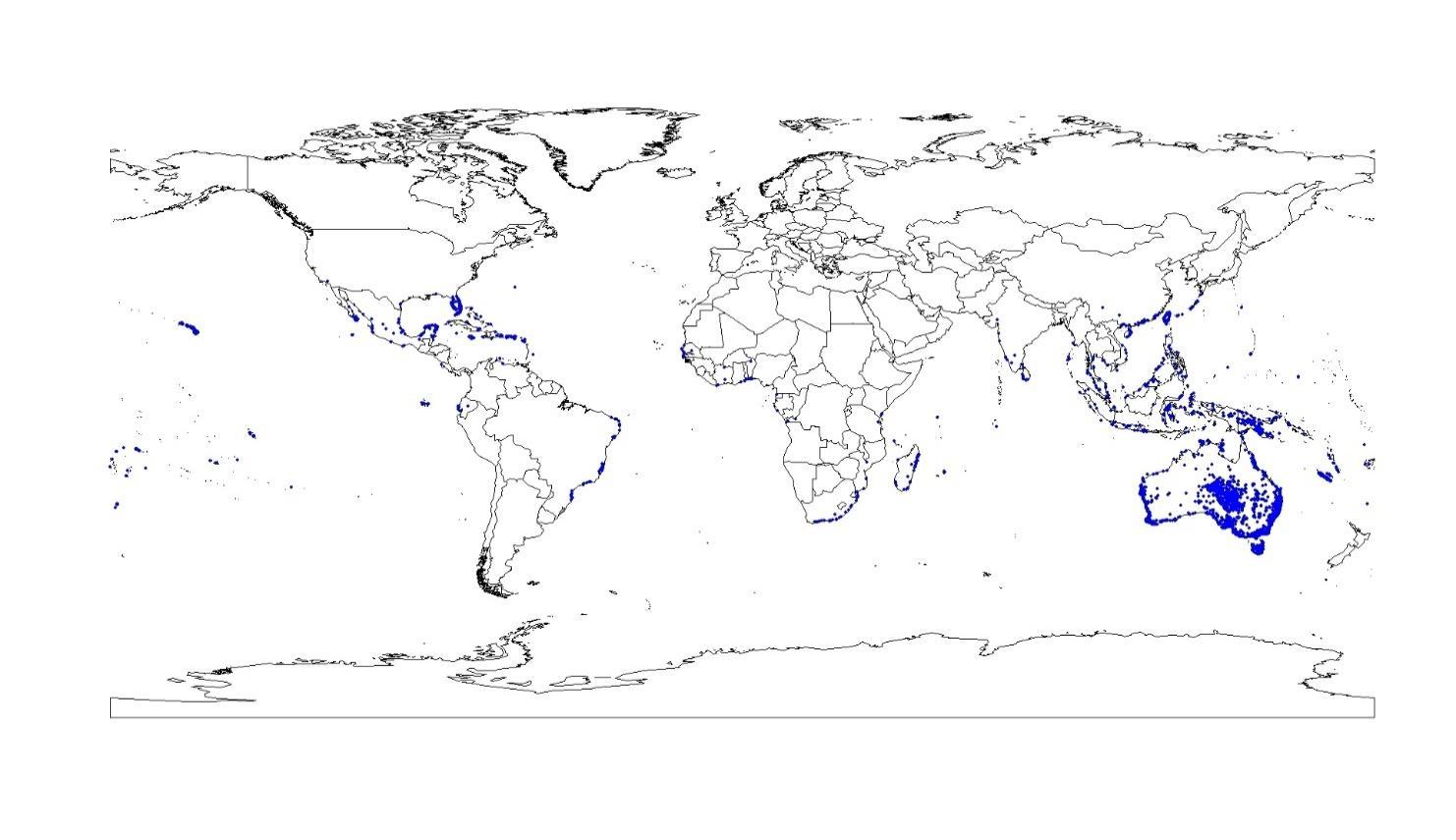
***

***Scaevola***

***
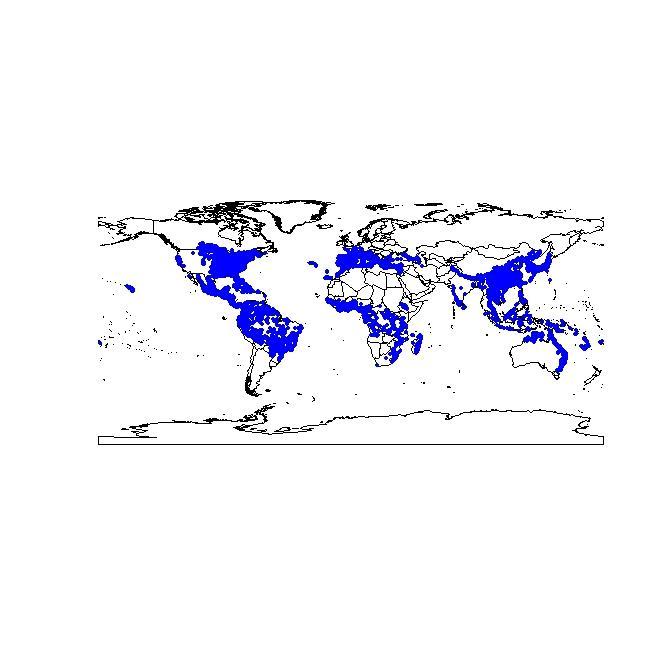
***

***Smilax***

***
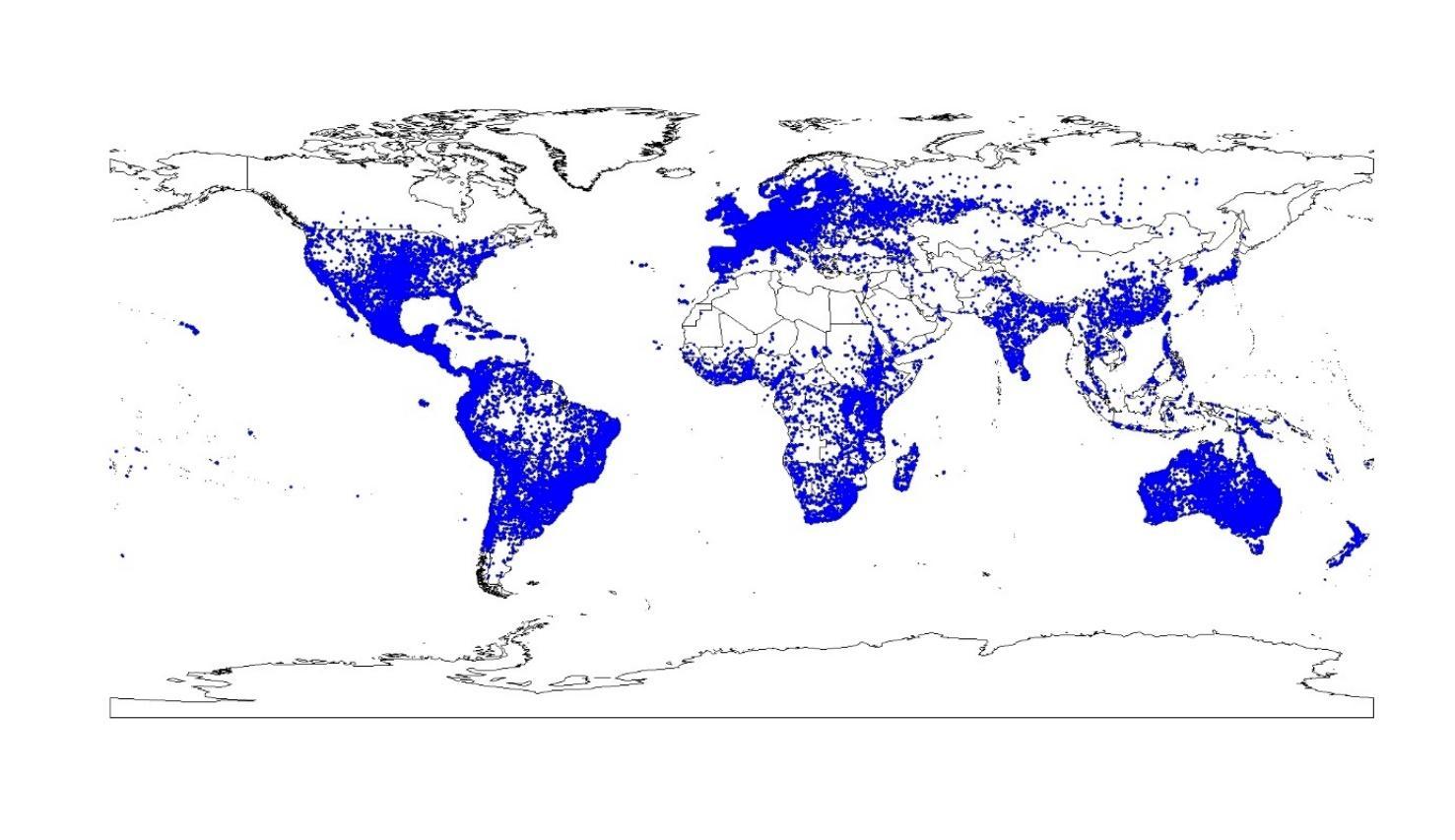
***

***Solanum***

***
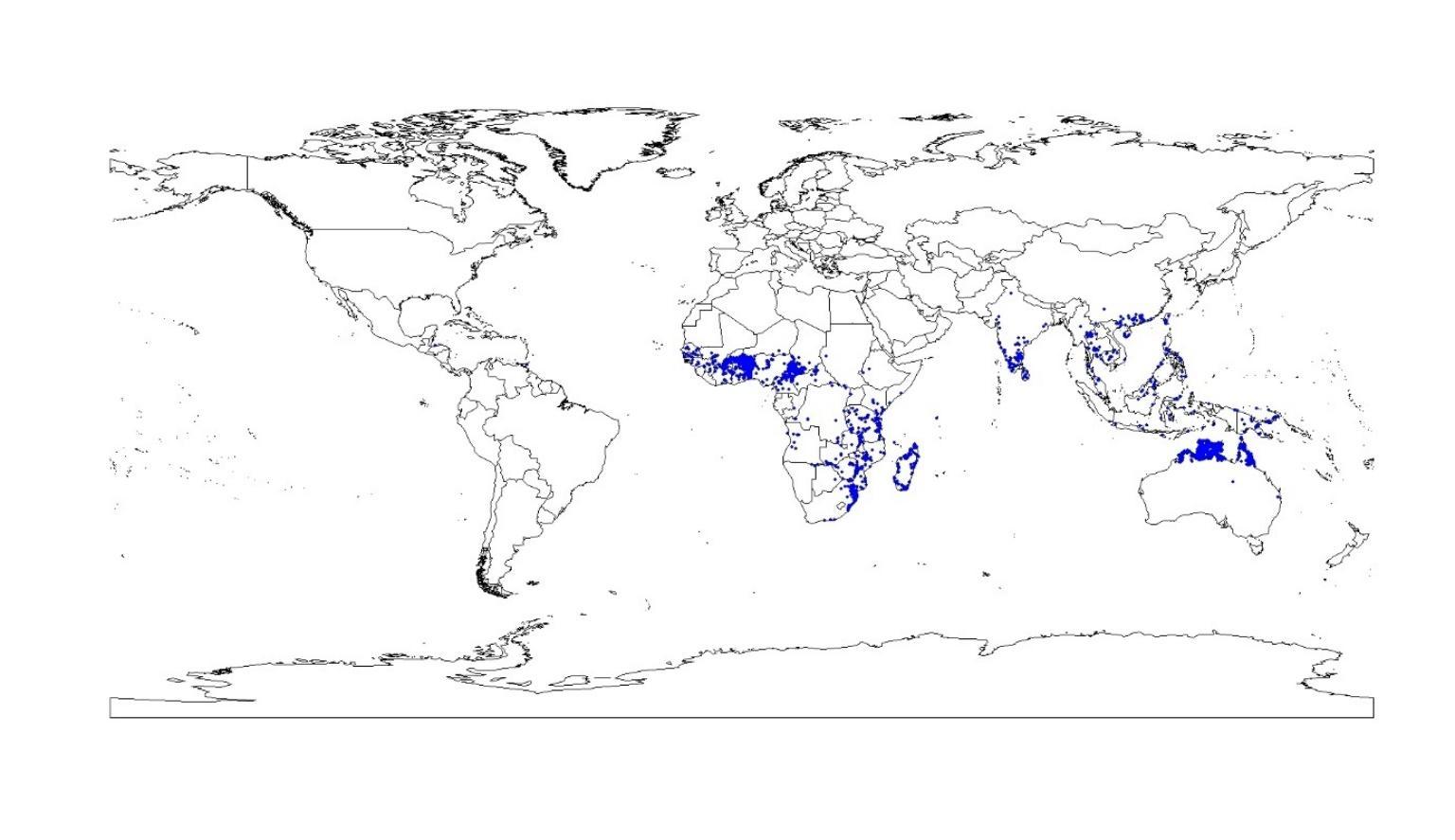
***

***Stychnos***

***
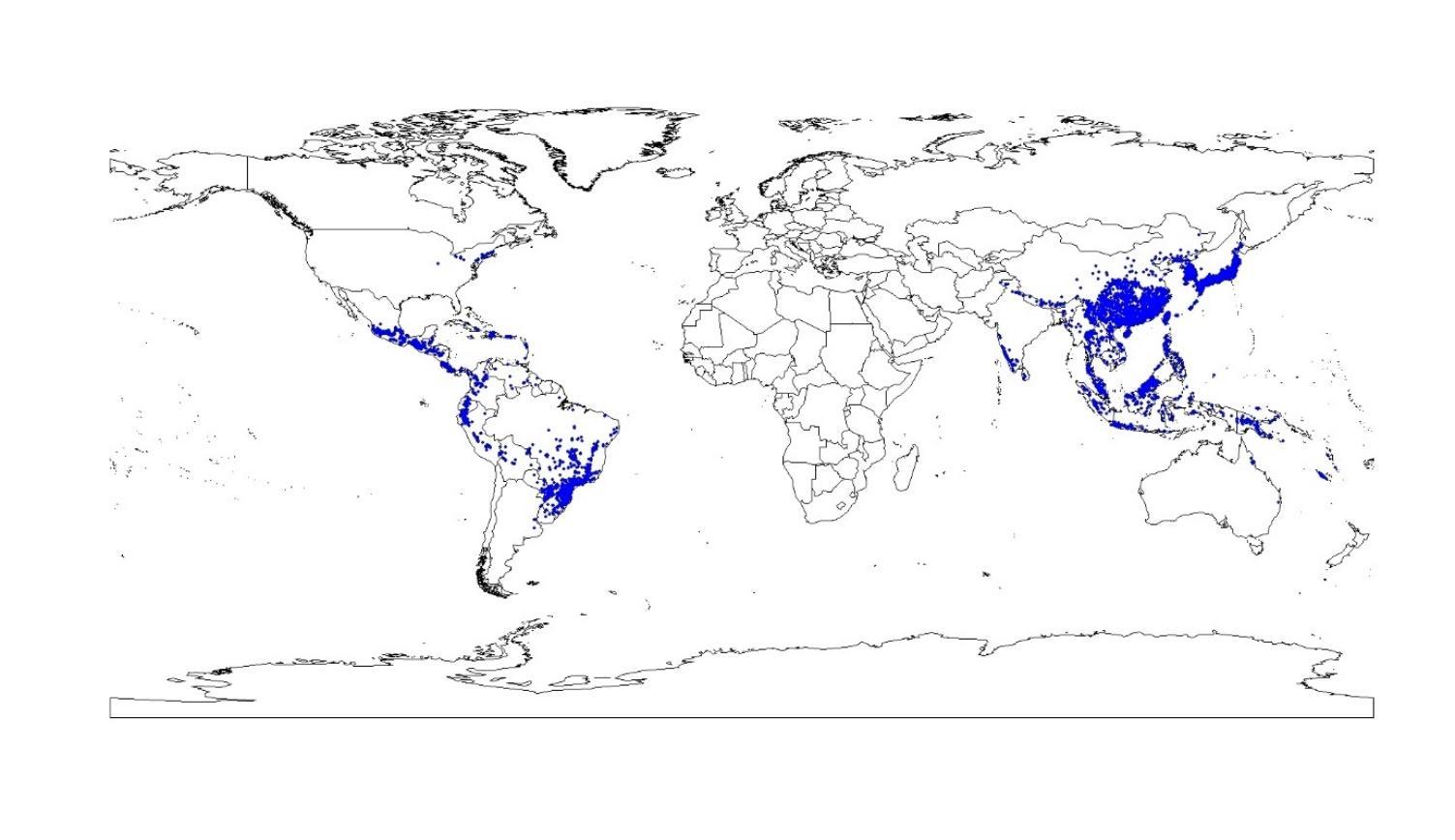
***

***Symplocos***

***
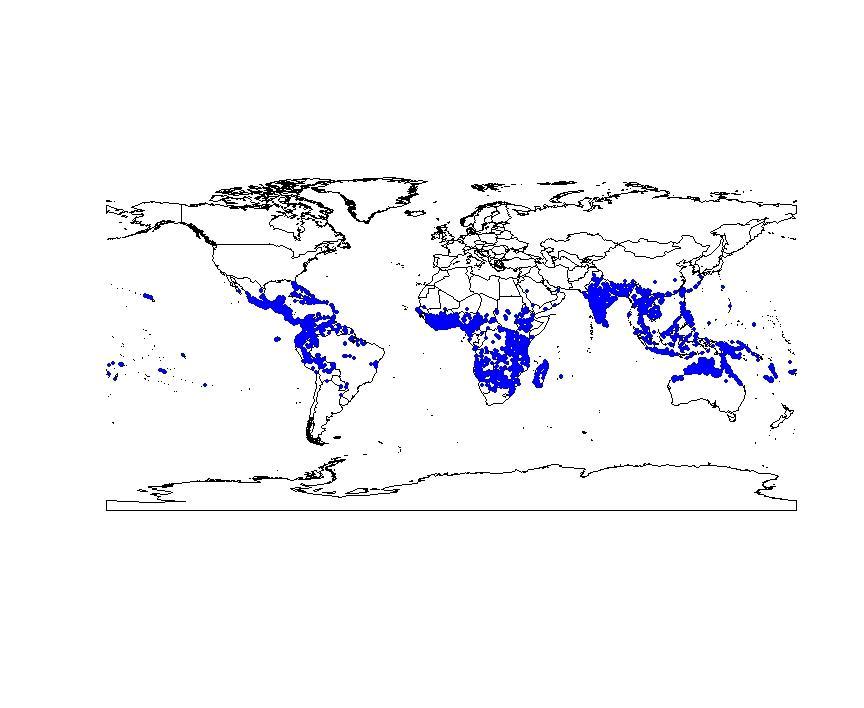
***

***Terminalia***

***
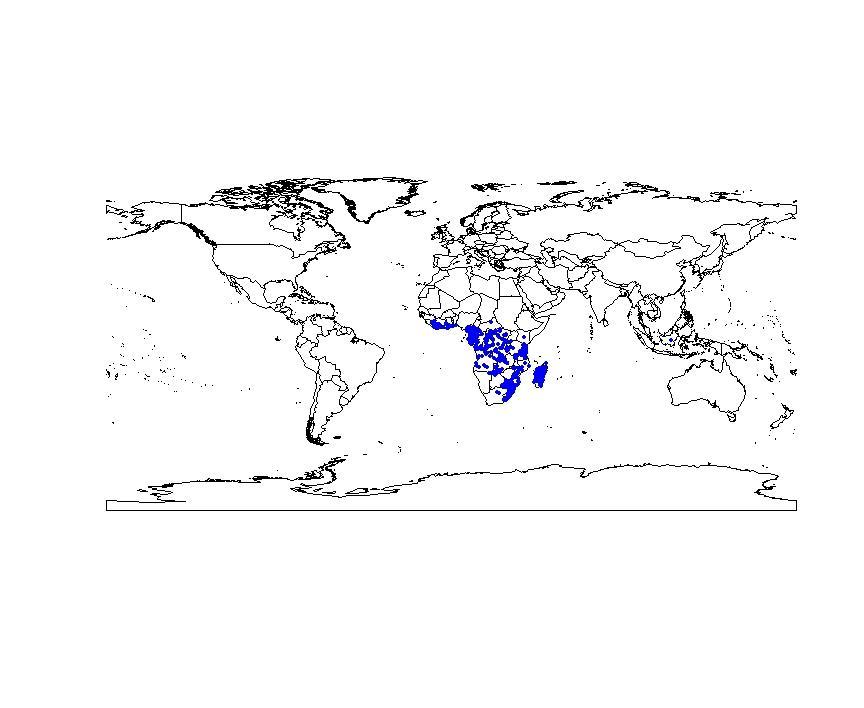
***

***Tricalysia***

***
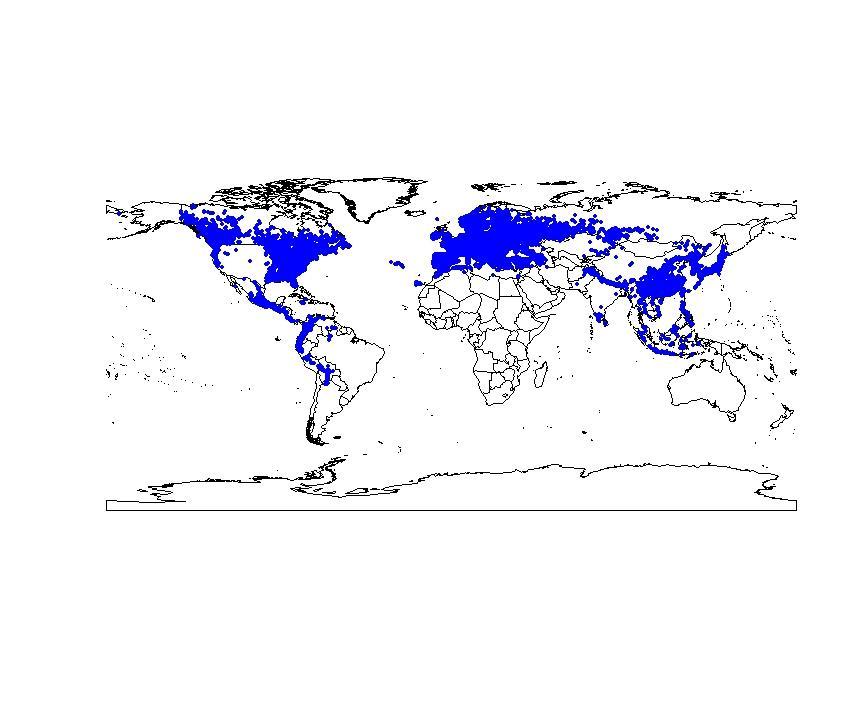
***

***Viburnum***

***
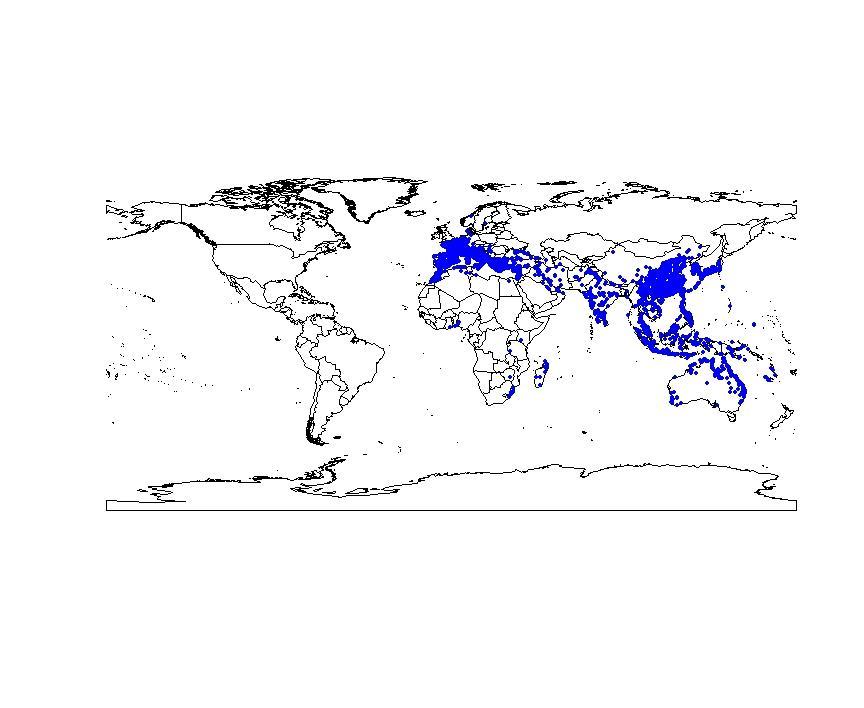
***

***Vitex***

**Figure S2. Pruned ranges of study clades**, with each dot representing an occurrence record for the species of the respective clade included in this study. While we paid great attention to pruning occurrence records through multiple approaches (using CoordinateCleaner, filtering against non-native occurrences with the GloNAF database, comparing clade-specific range maps with distributions on Plants of the World, manually screening individual clades and regions with unusually high species richness), we by no means claim that the range maps for the clades or study species are perfect.

***
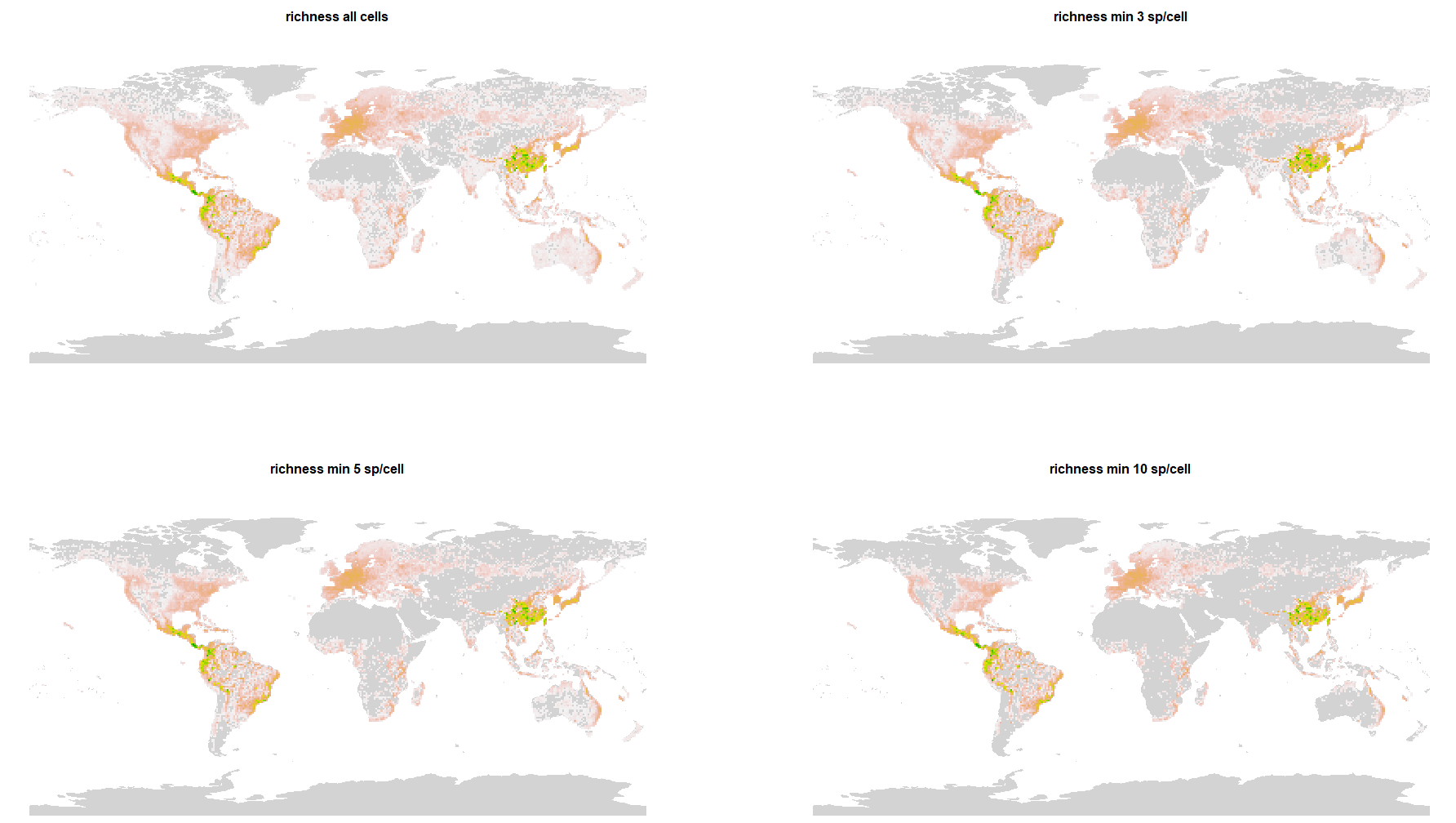
***

***
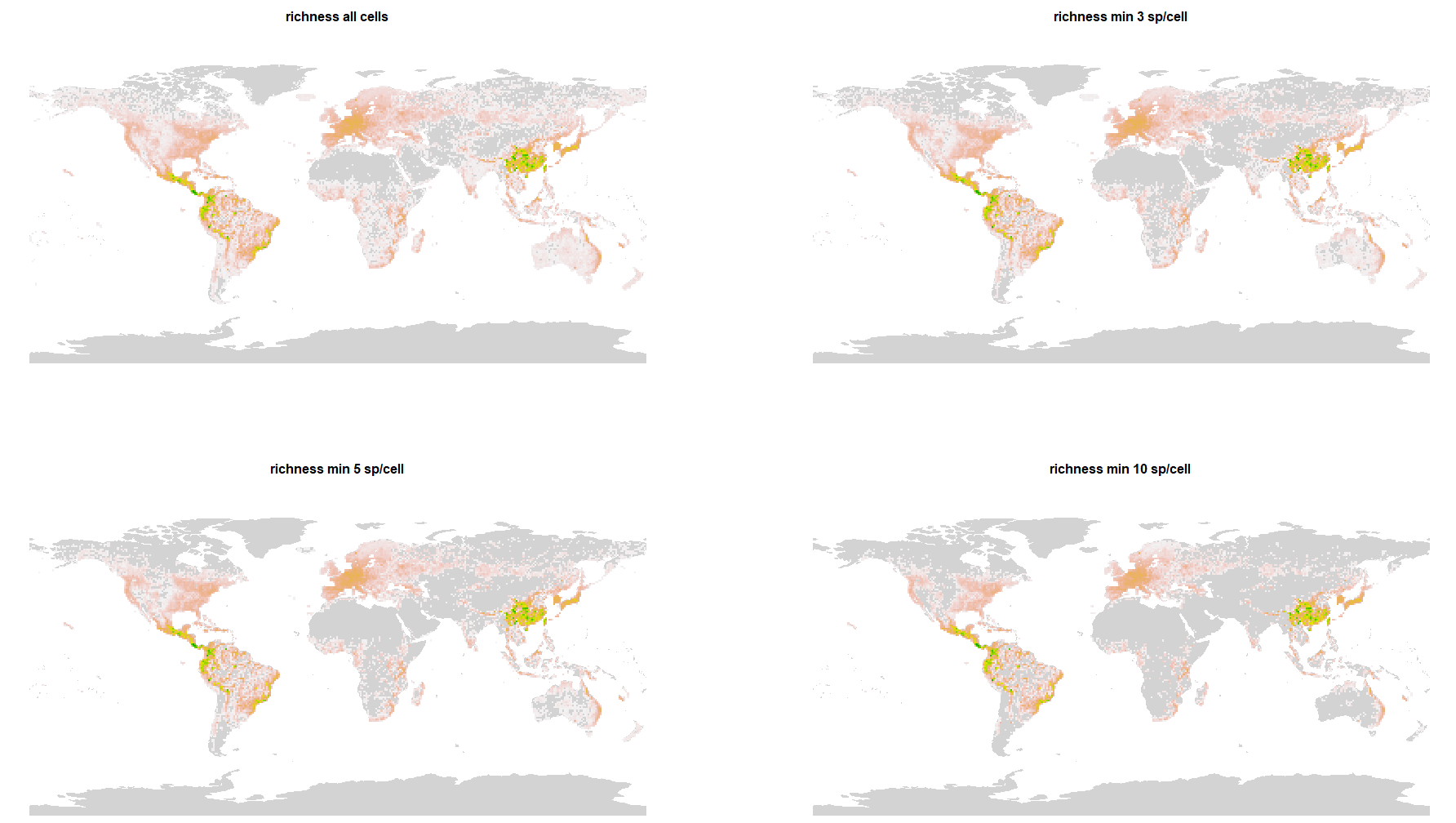
*Figure S3. Species richness when considering all grid cells**. Grid cells contained a minimum of 3 species/cell, a minimum of 5 species/cell, and a minimum of 10 species per cell. Note that coverage changes due to lower sampling in certain parts of the world. We based our analyses on the full data set (all grid cells) for calculating median occurrences per species, and a minimum of 3 species/cell or 10 species/cell for calculating the Shannon diversity index.


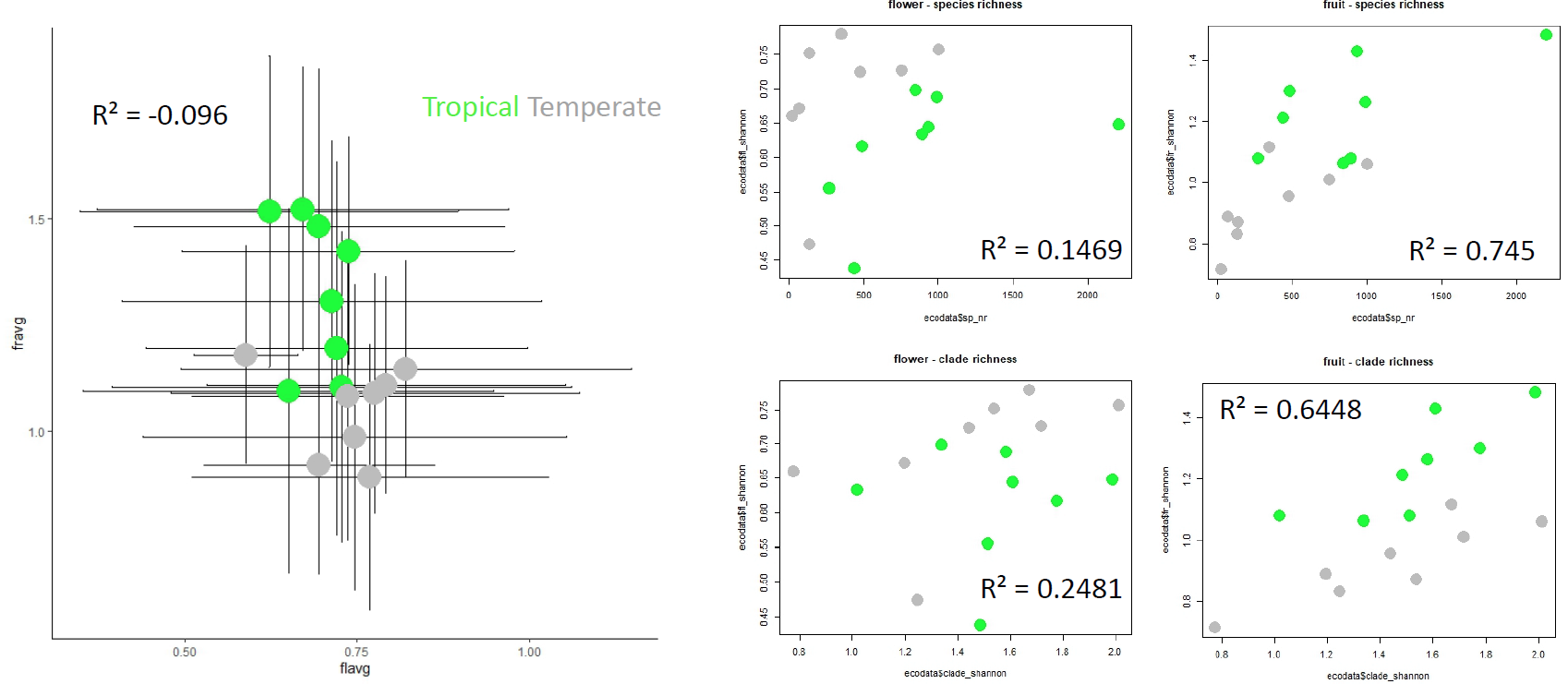


**Fig. S4. Shannon diversity values for flower (flavg) and fruit (fravg) color**. Each dot represents the average across grid cells per biome. No correlation among diversity values for fruit and flower color, low positive correlation between flower color diversity and species richness as well as clade richness, strong positive correlation between fruit color diversity and species richness as well as clade richness.


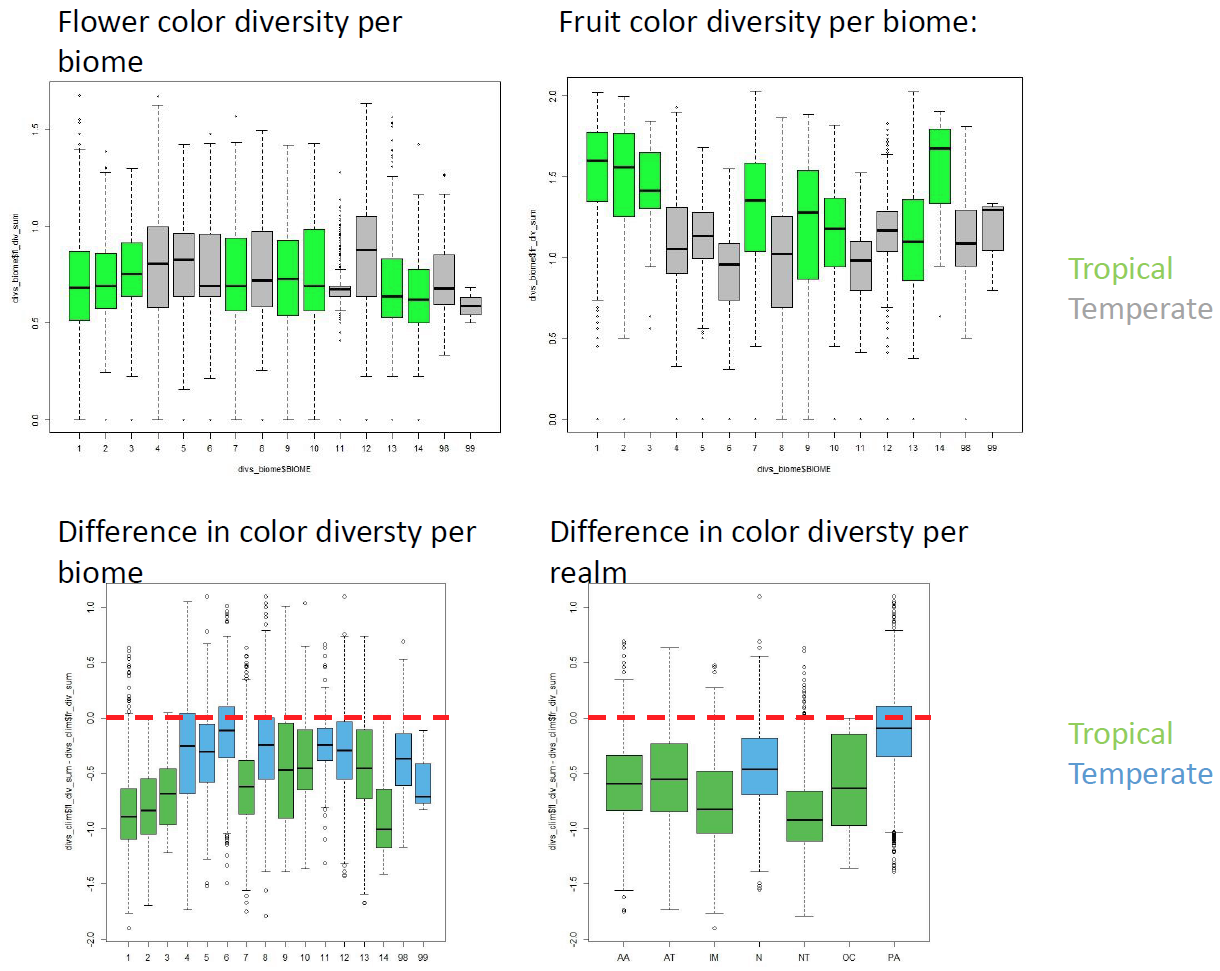


**Fig. S5. Color diversity (*y*-axis) for the different biomes (top panels) and difference in flower and fruit color diversity (bottom panels) for biomes and realms**. Red line in lower panel marks the zero line, which represents no difference between flower and fruit color diversity; values larger than 0 represent higher flower color diversity, values smaller than 0 represent higher fruit color diversity. “Tropical” biomes: 1, tropical and subtropical moist broadleaf forests; 2, tropical and subtropical dry broadleaf forests; 3, tropical and subtropical coniferous forests; 7, tropical and subtropical grasslands, savannas and shrublands; 9, flooded grasslands, savannas and shrublands; 10, montane grasslands, savannas and shrublands; 13, deserts and xeric shrublands; 14, mangroves; “temperate” biomes: 4, temperate broadleaf and mixed forests; 5, temperate coniferous forests; 6, boreal forests, taiga; 8. temperate grasslands, savannas and shrublands; 11, tundra; 12, Mediterranean forests, woodlands, and scrub or sclerophyll forests; 98, lakes; 99, Arctic (Rock and Ice/Greenland). “Tropical” realms: AA, Austral-Asia; AT, afro-tropics; IM, Indo-Malaysia; NT, neotropics; O, Oceania; “Temperate” realms: N, nearctic; PA, palearctic.


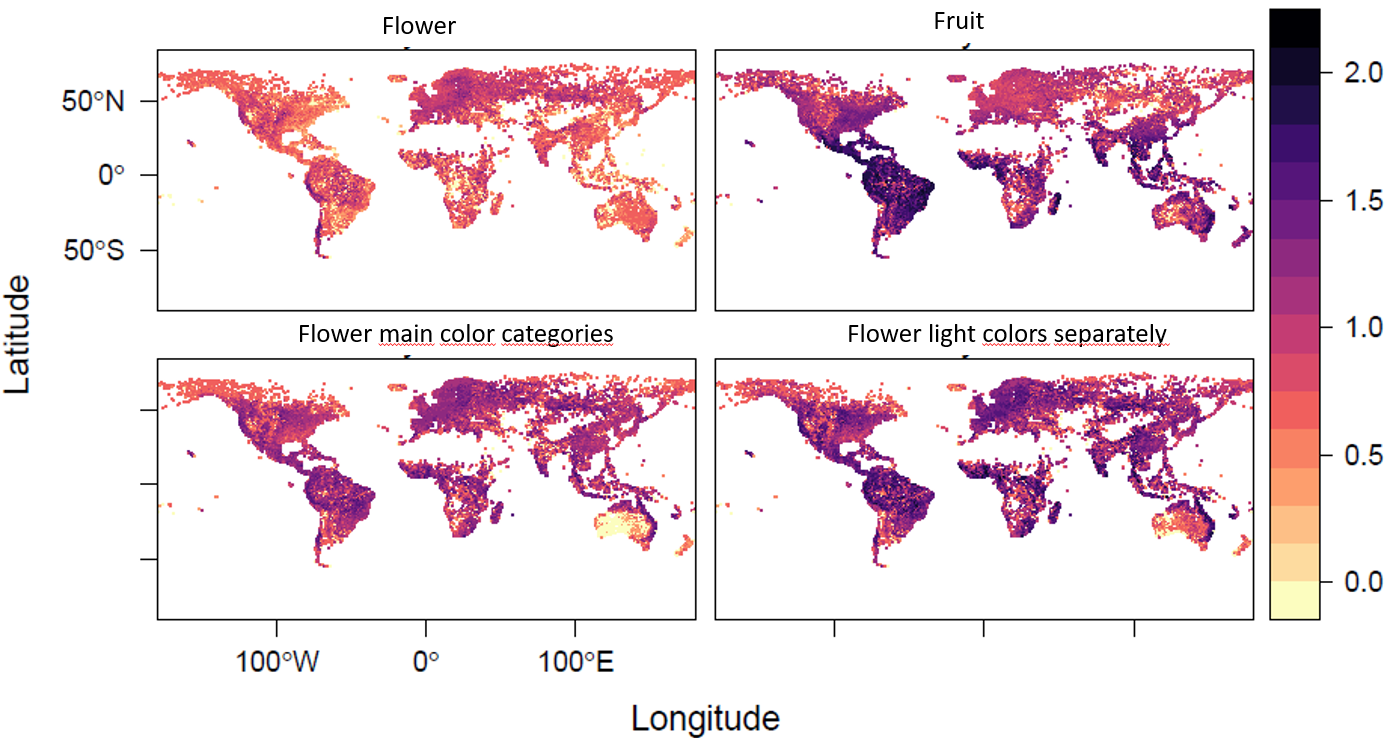


**Figure S6. Shannon diversity values for flower and fruit color** and different diversity patterns when categorizing flower colors differently (bottom plots). The top plots (Flower, Fruit) are the categorization schemes used throughout our study (Figure 1), while the bottom plots either lump all flower colors into the main color category regardless of color intensity (i.e., Y1, Y2, Y3, Y4, Y5 are all lumped as “yellow”) or keep light colors (Y1, Pi1, Pu1, R1, G1) separate from white, while also retaining the other main colors (11 categories); compare with Table S3. Note the general increase in color diversity for flowers with the different categorization schemes, but consistent differences between flower and fruit colors; i.e., flower color diversity is higher in temperate Eurasia.


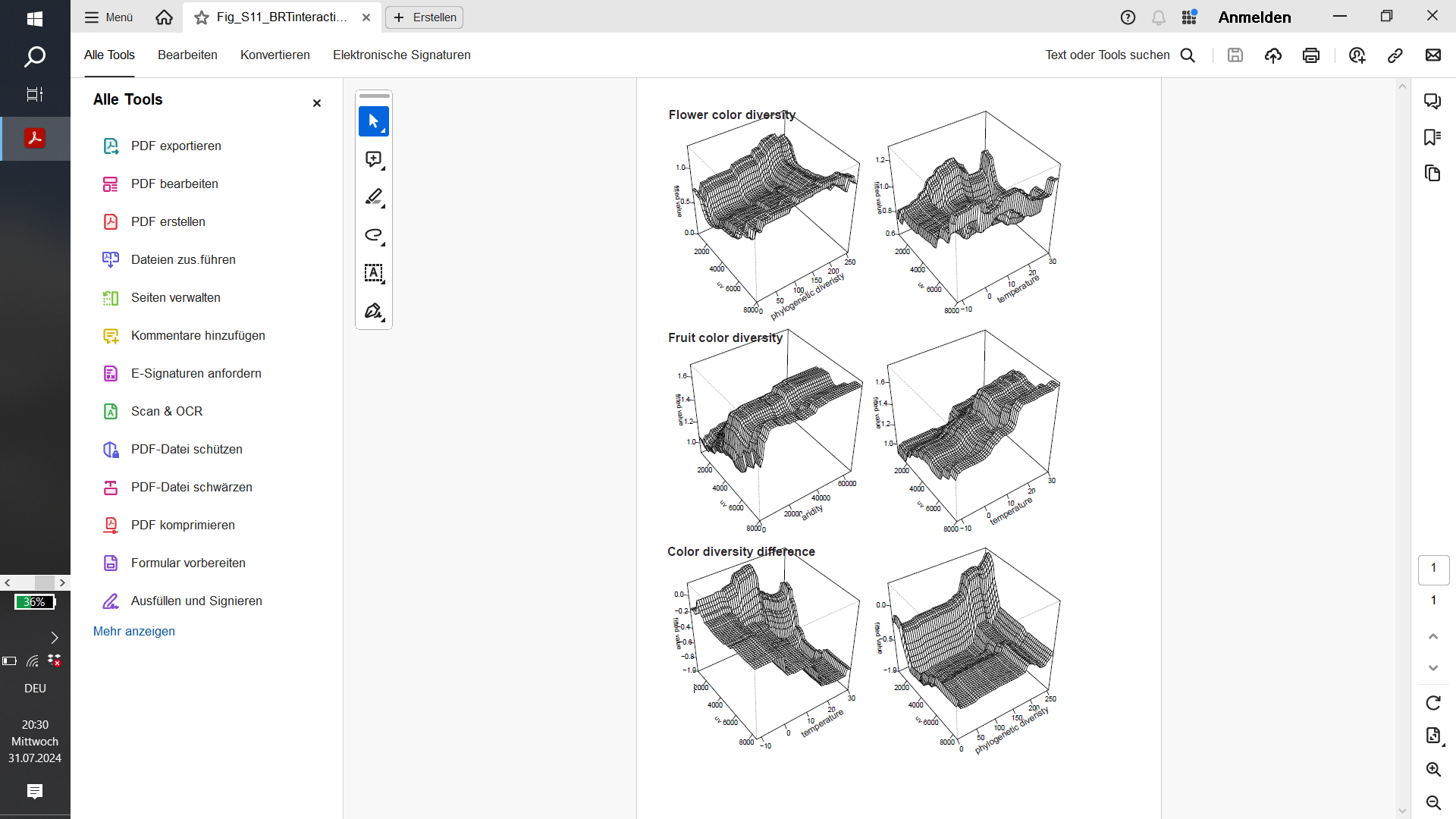


**Figure S7**. **The 2 strongest interactions between predictor variables of boosted regression tree models** (Table S9) based on a minimum of 5 species per grid cell and 75% training data. Flower color diversity increased with increasing phylogenetic diversity and at low and high UV-B and intermediate and very high temperatures. Fruit color diversity is highest at high UV-B, particularly in warm areas of increasing humidity. The difference between flower and fruit color diversity is strongest (large negative values) at high temperatures and high UV-B exposure and under low phylogenetic diversity.


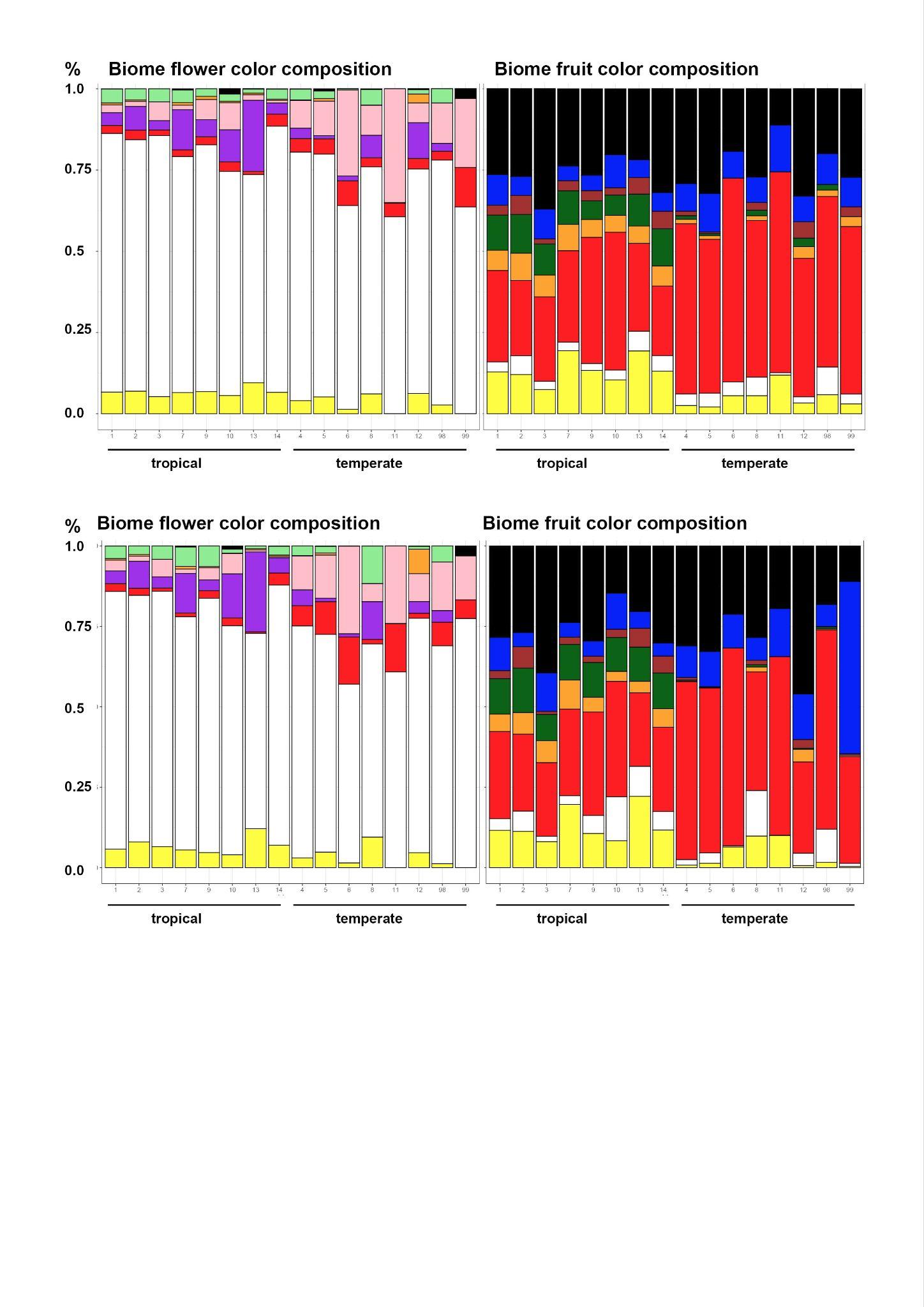


**Figure S8.** **Flower and fruit color composition differed across tropical and temperate biomes**, data subsampled to 1 occurrence per species per 1 x 1 km grid cell (compare with Fig. 2 of main text for sampling to 1 x 1 degree grid cell).


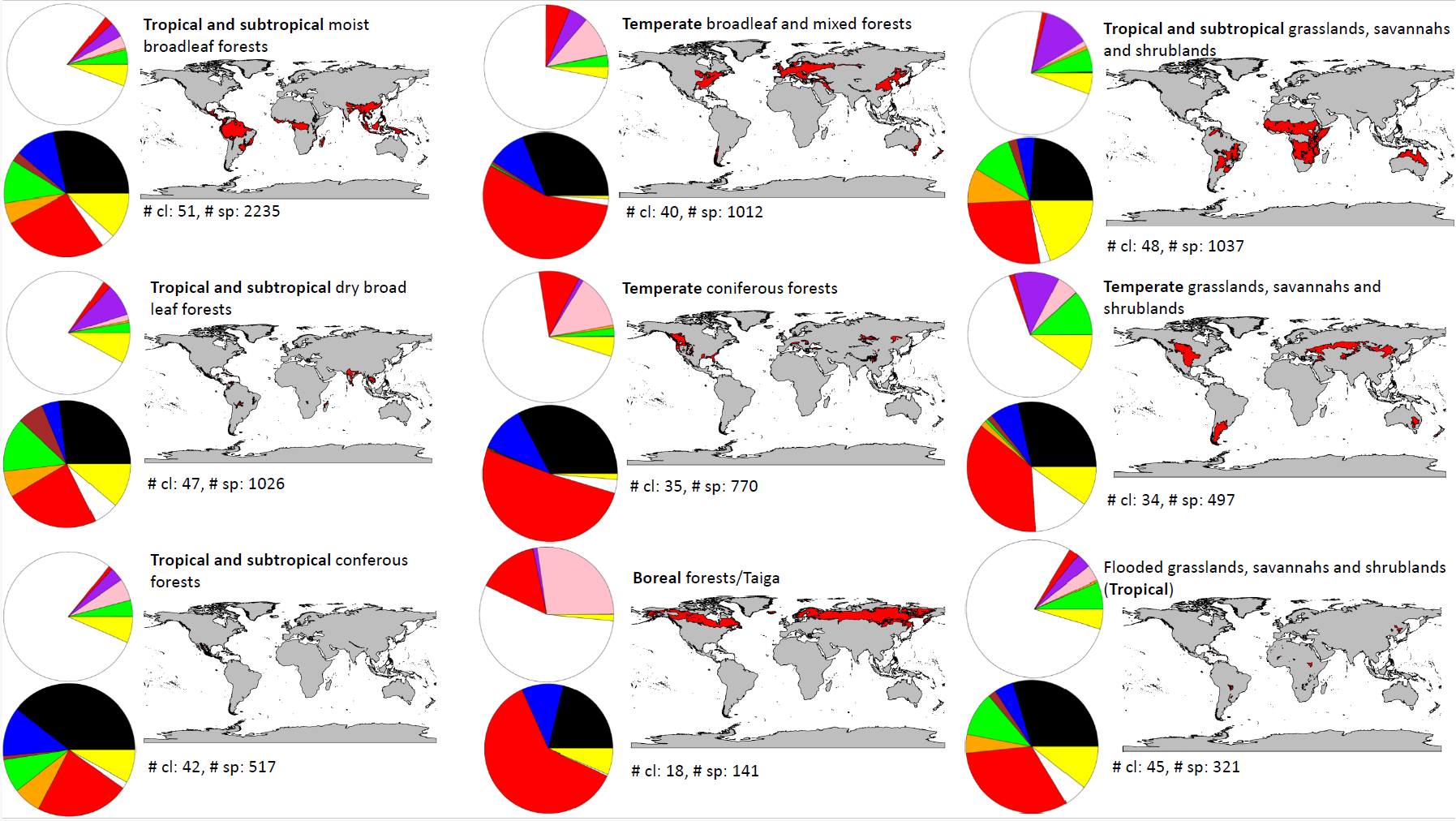


**Figure S9. Flower and fruit color composition mapped for the different biomes** (biomes 1–9 shown here, in order of appearance), with each biome’s upper chart representing flower colors, each lower chart representing fruit colors.


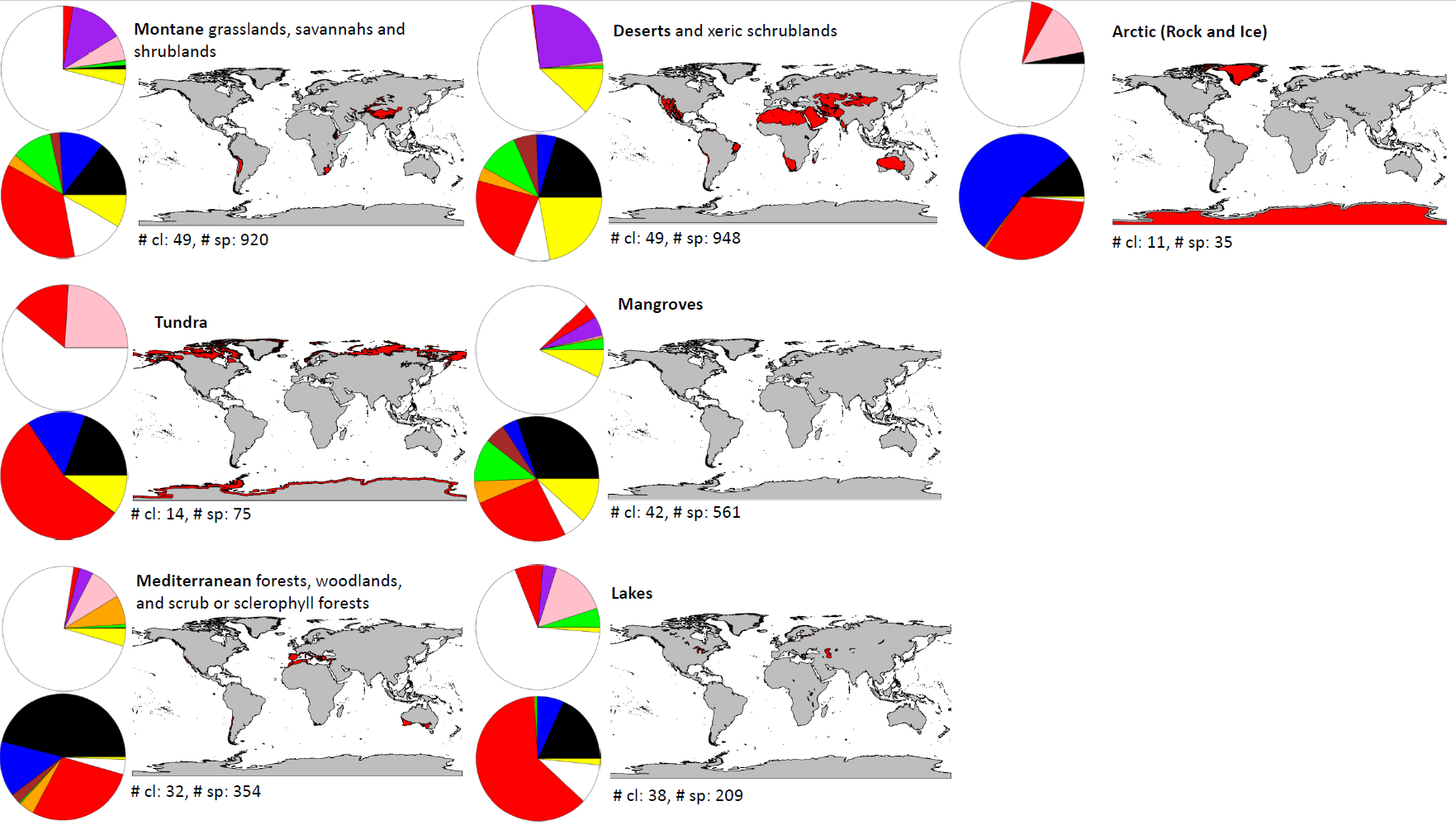


**Figure S10. Flower and fruit color composition mapped for the different biomes** (biomes 10–14, 89, 99 shown here, in order of appearance).


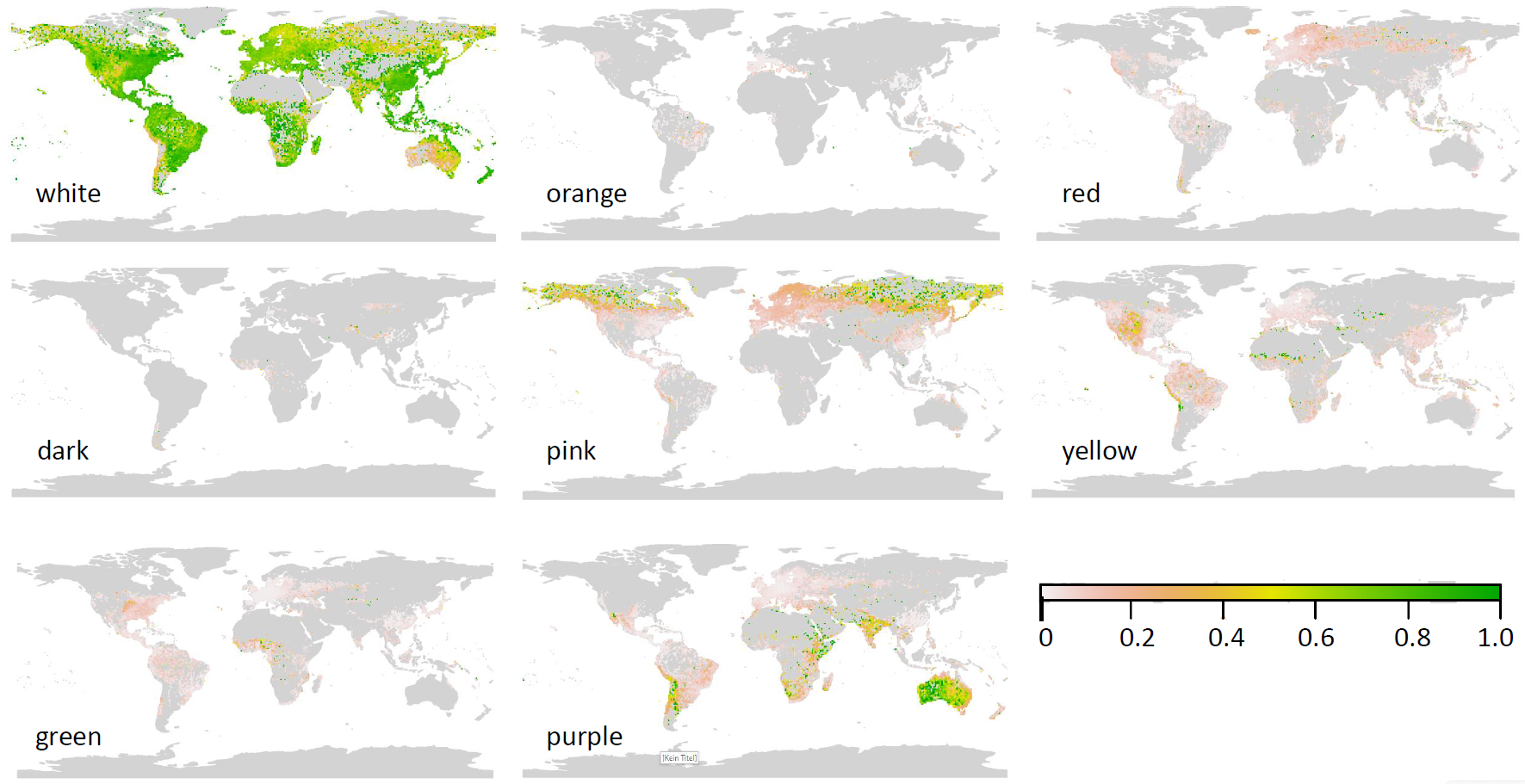


**Figure S11. Global distribution of individual flower colors of the 2815 species of fleshy-fruited clades included in our data set,** scaled by absolute species richness, so that a value of 1 (dark green) indicates that 100% of species in a grid cell are the respective color, and a value of 0 (white) indicates that all species in that grid cell are a different color. White is the most common color across the globe; anthocyanin-based red and pink are more common toward temperate biomes; purple flowers are more common in arid regions.


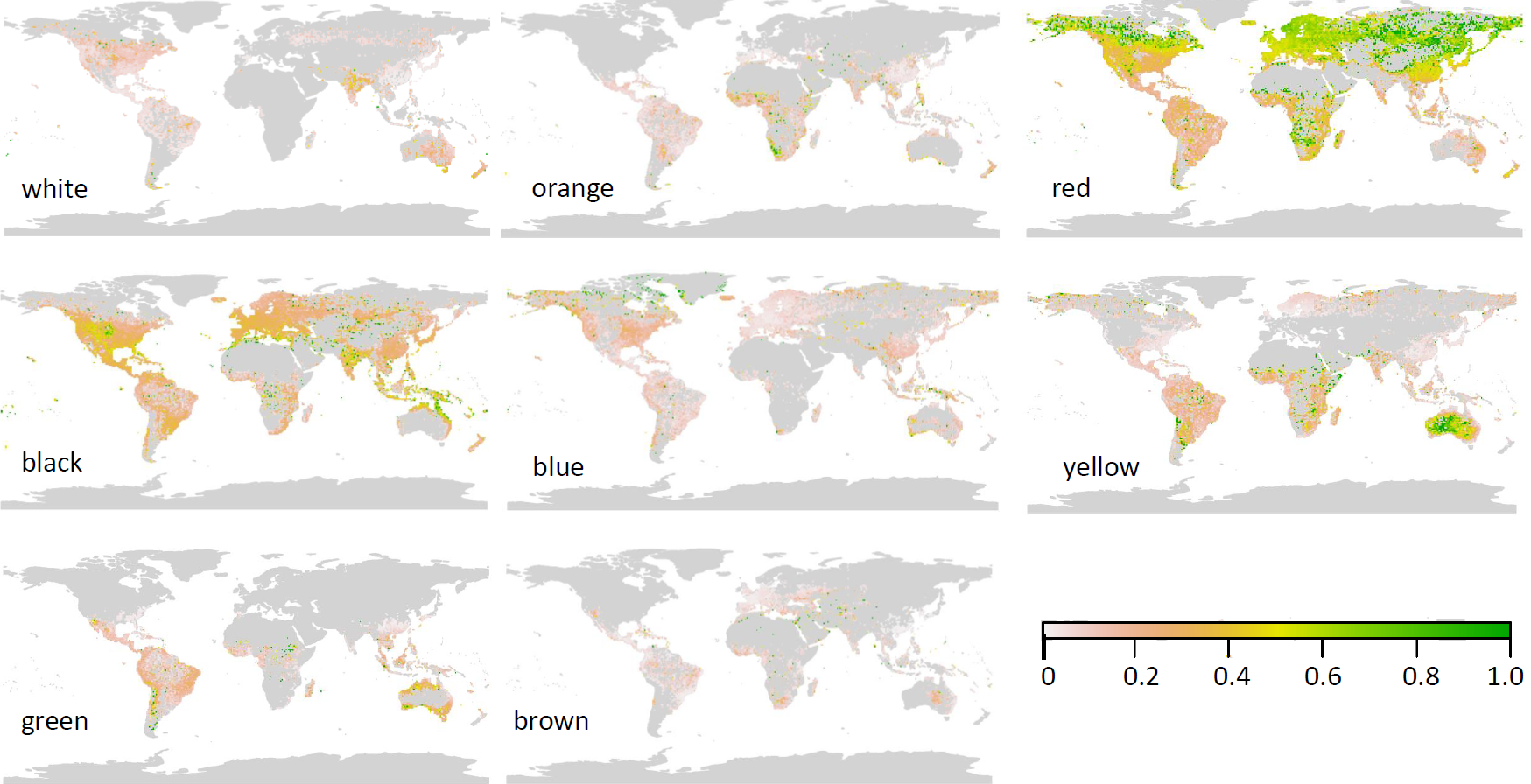


**Figure S12**. **Global distribution of individual fruit colors of the 2815 species of fleshy-fruited clades included in our data set**, scaled by absolute species richness, so that a value of 1 (dark green) indicates that 100% of species in a grid cell are the respective color, and a value of 0 (white) indicates that all species in that grid cell are a different color. Red and black fruits are the most dominant fruit colors in the temperate north, while yellow, green, and orange fruits are more abundant in the southern hemisphere, and yellow fruits are particularly common in arid regions.


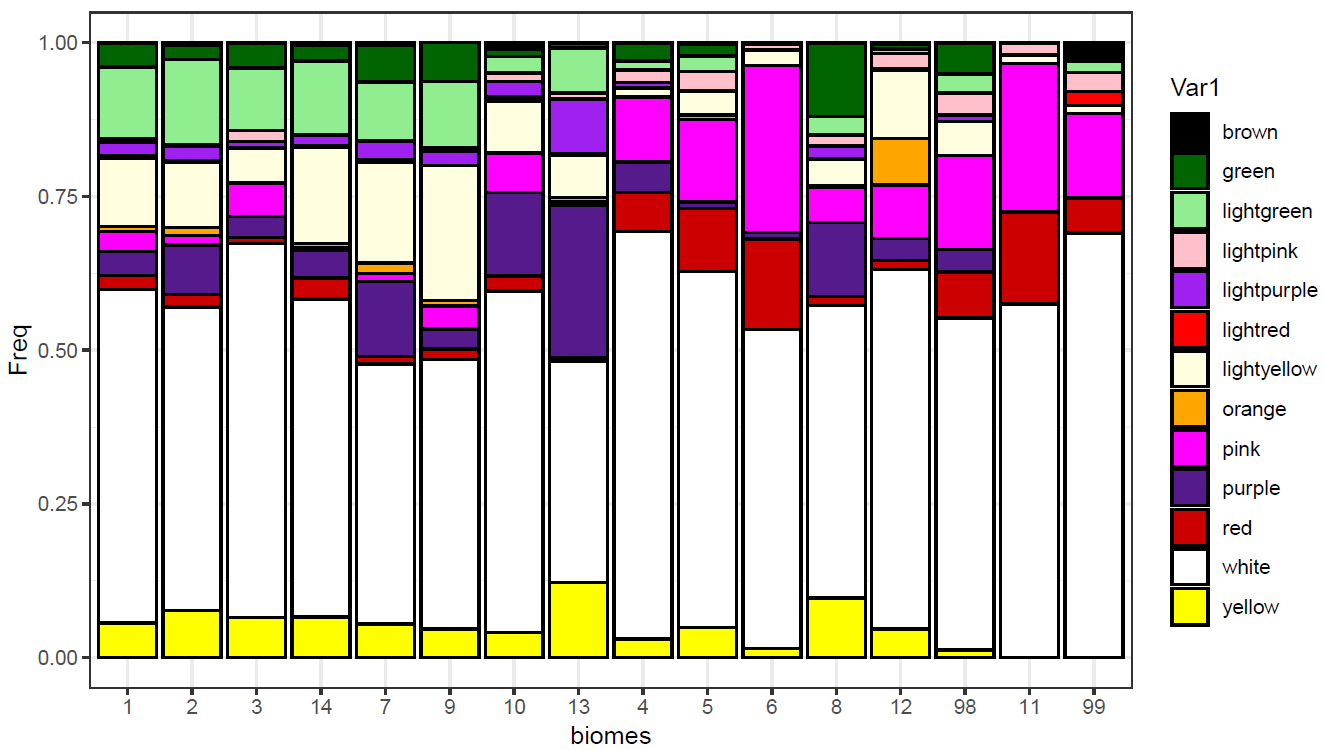


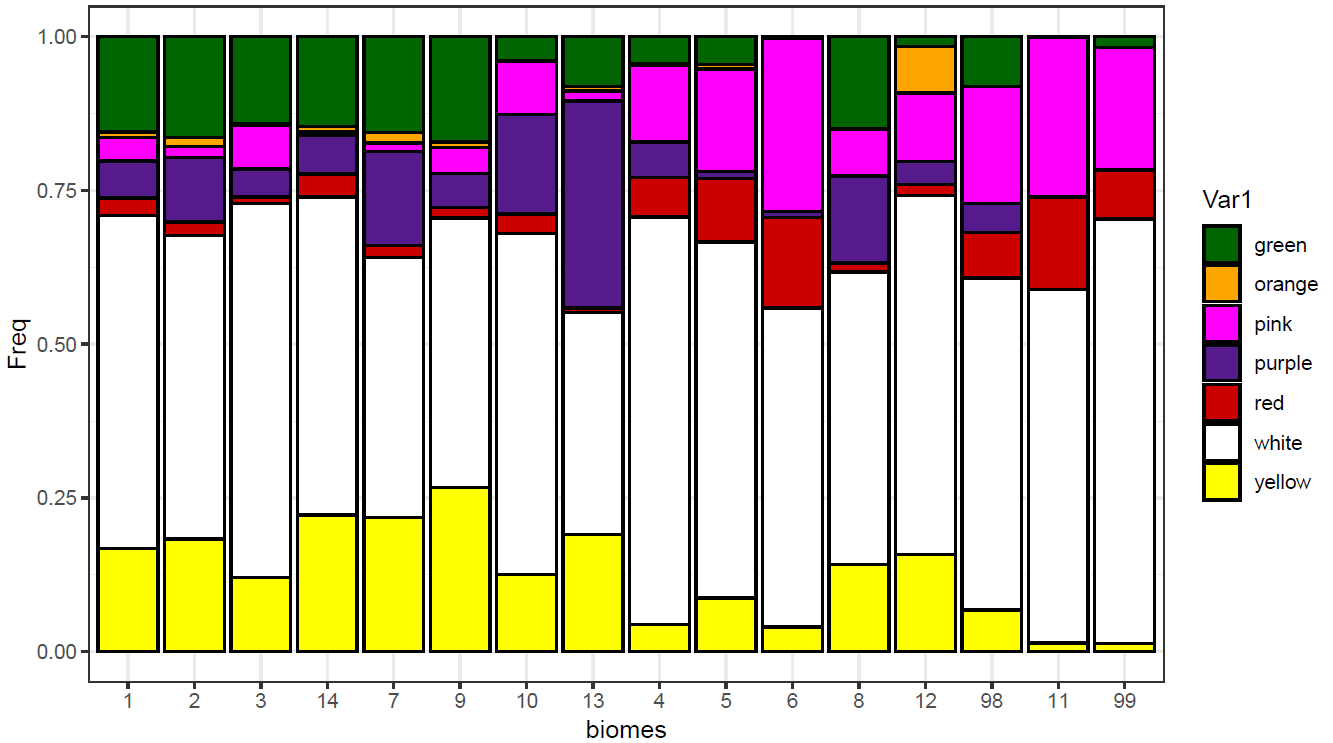


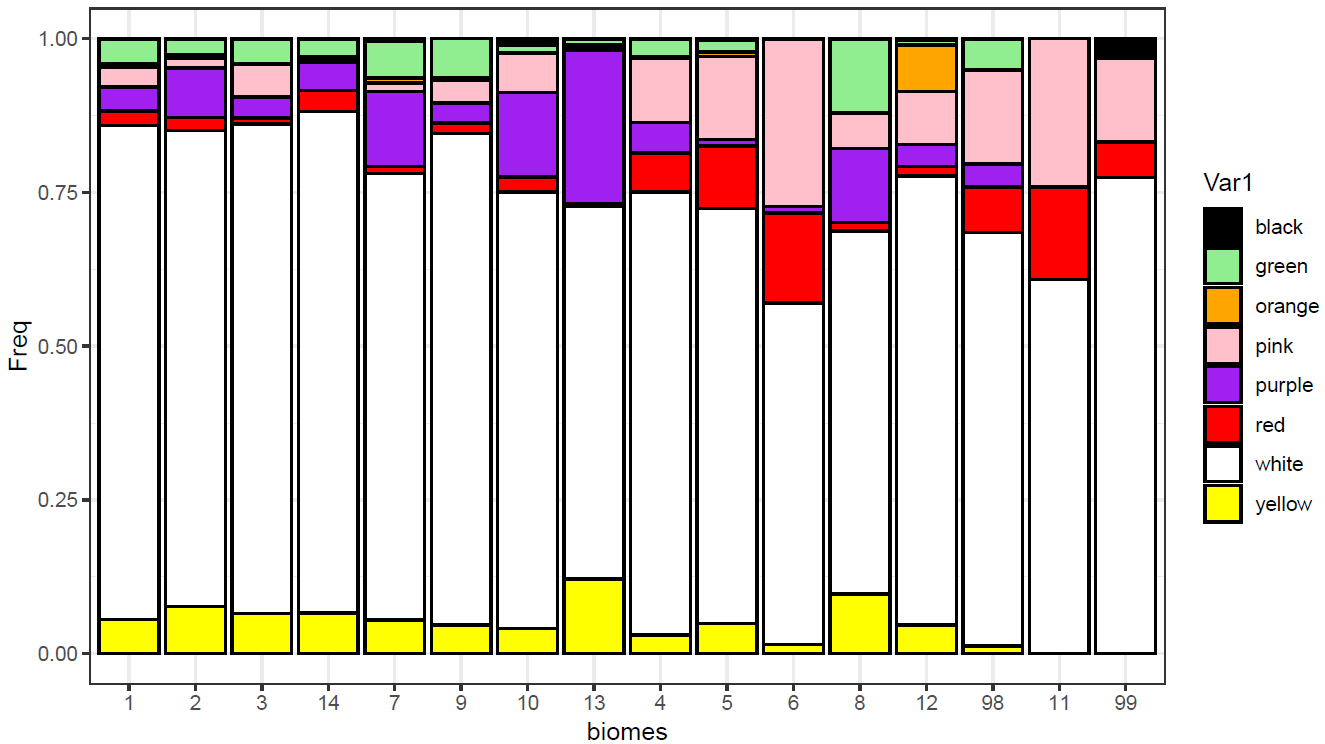


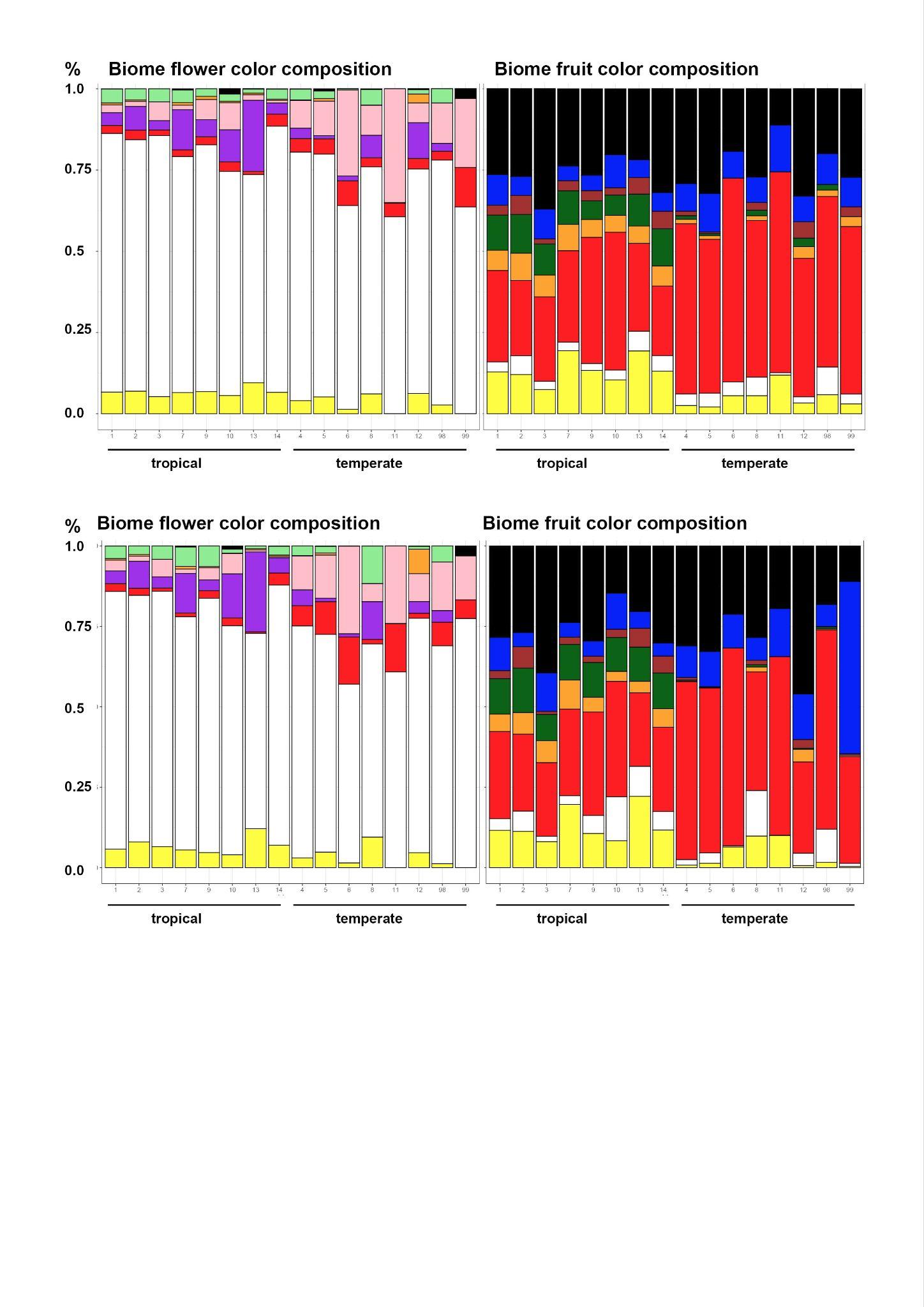


**Figure S13**. **Comparison of the different color categorizations for flowers**. The top graph shows light shades classified separately, the middle graph shows light and dark colors lumped into the main color category, and the bottom graph shows light colors merged with white, dark categories merged into “black” and categories 2, 3, and 4 summarized into the main colors. The proportion of white flowers is reduced in categorization schemes 1 and 2; however, the general pattern remains the same in that purple flowers are more abundant in arid, open biomes (e.g., savannahs, grasslands, deserts) and pink and red flowers are more abundant in temperate, cold biomes. Light yellow and light green are particularly common in biomes classified as “tropical” (1, 2, 3, 14, 7, 9, 10, 13).

**Table S1. Number of GBIF occurrences and species retained** after custom filtering techniques through CoordinateCleaner and matching occurrences against the GloNAF invasive species plant database (no. of occurrences before, no. of species before), and after additional pruning steps by comparing the occurrences against reported clade ranges on the Plant of the World online database, and removing non-native ranges of cultivated species. Note that these numbers represent the raw occurrence records; for analyses, we subsampled the dataset to one occurrence per species per kilometer, hence absolute of records per clade were a little lower.

| **Clade** | **No. occurrences before filtering** | **No. species before filtering** | | **No. occurrences after filtering** | | **No. species after filtering** | | **No. occurrences after subsampling (1 km)** | | **Notes** | |
| --- | --- | --- | --- | --- | --- | --- | --- | --- | --- | --- | --- |
| *Annona* | 18218 | 32 | | 15496 | | 32 | | 10093 | | pruned manually. cultivated species | |
| *Asparagaus* | 85979 | 33 | | 50582 | | 33 | | 41687 | | pruned manually. cultivated species | |
| *Bactris* | 5060 | 28 | | 4936 | | 28 | | 4349 | | pruned manually. cultivated species | |
| *Calopyhllum* | 3842 | 12 | | 3579 | | 12 | | 2846 | |  | |
| *Canthium* | 6393 | 14 | 2518 | | 14 | | 2173 | |  | |  |
| *Capparis* | 24459 | 20 | | 19244 | | 20 | | 9989 | | pruned manually. cultivated species; wider genus delimitation (including Colicodendron and Cynophalla) | |
| *Casearia* | 9757 | 9 | | 9434 | | 9 | | 7870 | |  | |
| *Celtis* | 15127 | 12 | | 10106 | | 12 | | 8305 | | pruned manually | |
| *Chrysophyllum* | 10223 | 19 | | 4467 | | 18 | | 3686 | |  | |
| *Cissus* | 47037 | 75 | | 43510 | | 75 | | 34809 | | pruned manually. cultivated species | |
| *Clidemia* | 14889 | 81 | | 12932 | | 80 | | 10380 | |  | |
| *Cordia* | 41267 | 75 | | 39745 | | 75 | | 21223 | | pruned manually. cultivated species | |
| *Cornus* | 909512 | 37 | | 393903 | | 37 | | 307072 | | pruned manually. cultivated species | |
| *Crataegus* | 782778 | 39 | | 85251 | | 37 | | 71582 | | pruned manually. cultivated species | |
| *Cryptocarya* | 10807 | 27 | | 6835 | | 27 | | 5189 | | checked manually | |
| *Cyphostemma* | 1464 | 30 | | 1332 | | 30 | | 1161 | |  | |
| *Diospyros* | 32798 | 118 | | 30386 | | 118 | | 20248 | | pruned manually. cultivated species | |
| *Elaeocarpus* | 39116 | 66 | | 38710 | | 66 | | 28226 | | checked manually | |
| *Endiandra* | 2211 | 10 | | 1394 | | 10 | | 1138 | |  | |
| *Erythroxylum* | 1539 | 6 | | 1425 | | 6 | | 1251 | |  | |
| *Garcinia* | 7522 | 58 | | 7184 | | 58 | | 6200 | | pruned manually. cultivated species | |
| *Gaultheria* | 74085 | 76 | | 63795 | | 76 | | 43294 | | pruned manually. cultivated species | |
| *Grewia* | 2186 | 3 | | 2060 | | 3 | | 1759 | |  | |
| *Ilex* | 402874 | 127 | | 191725 | | 125 | | 145916 | | pruned manually. cultivated species | |
| *Iochroma* | 1256 | 18 | | 617 | | 18 | | 528 | |  | |
| *Lonicera* | 462942 | 83 | | 432903 | | 82 | | 331746 | | pruned manually. cultivated species | |
| *Maytenus* | 8463 | 34 | | 7746 | | 34 | | 6516 | |  | |
| *Nectandra* | 17099 | 29 | | 11073 | | 29 | | 8529 | | checked manually | |
| *Ochna* | 5845 | 9 | | 1602 | | 9 | | 1259 | |  | |
| *Ocotea* | 14794 | 45 | | 13850 | | 45 | | 11175 | |  | |
| *Passiflora* | 66885 | 184 | | 49690 | | 181 | | 41814 | | pruned manually. cultivated species | |
| *Pavetta* | 1368 | 13 | | 1294 | | 13 | | 1138 | |  | |
| *Pouteria* | 17501 | 61 | | 15620 | | 61 | | 12467 | | pruned manually. cultivated species | |
| *Protium* | 23220 | 47 | | 13384 | | 47 | | 10620 | |  | |
| *Prunus* | 1720172 | 92 | | 722392 | | 90 | | 564449 | | pruned manually. cultivated species | |
| *Rhamnus* | 180823 | 19 | | 148913 | | 19 | | 114354 | | pruned manually. cultivated species | |
| *Rhus* | 166778 | 40 | | 149263 | | 40 | | 88154 | | pruned manually. cultivated species | |
| *Ribes* | 518346 | 83 | | 243020 | | 80 | | 204143 | | pruned manually. cultivated species | |
| *Rosa* | 409720 | 86 | | 388445 | | 85 | | 329040 | | pruned manually. cultivated species | |
| *Rubus* | 2302629 | 88 | | 837308 | | 85 | | 688856 | | pruned manually. cultivated species | |
| *Scaevola* | 14267 | 33 | | 14370 | | 33 | | 10336 | |  | |
| *Smilax* | 110023 | 89 | | 108099 | | 89 | | 81957 | | pruned manually | |
| *Solanum* | 539524 | 441 | | 365562 | | 434 | | 282341 | | pruned manually. cultivated species | |
| *Sorbus* | 401650 | 14 | | 398863 | | 14 | | 279989 | |  | |
| *Strychnos* | 4953 | 8 | | 4908 | | 7 | | 4144 | |  | |
| *Symplocos* | 30241 | 95 | | 28944 | | 94 | | 25122 | |  | |
| *Terminalia* | 16138 | 36 | | 15542 | | 36 | | 13051 | |  | |
| *Tricalysia* | 1017 | 10 | | 1006 | | 10 | | 894 | |  | |
| *Vaccinium* | 725456 | 57 | | 721658 | | 57 | | 530389 | | pruned manually. cultivated species | |
| *Viburnum* | 446412 | 123 | | 416903 | | 120 | | 312725 | | pruned manually. cultivated species | |
| *Vitex* | 17046 | 4 | | 16291 | | 4 | | 11587 | | pruned manually | |

**Table S2. Family and order information and DOI links to individual data sets downloaded from GBIF for each plant clade.**

| **Clade** | **Family** | **Order** | **DOI** | **2nd Round** |
| --- | --- | --- | --- | --- |
| *Annona* | Annonaceae | Magnoliales | [10.15468/dl.6n27nz](https://doi.org/10.15468/dl.6n27nz) | 10.15468/dl.3at8wr |
| *Asparagus* | Asparagaceae | Asparagales | 10.15468/dl.5z3dkj | [10.15468/dl.vmmewe](https://doi.org/10.15468/dl.vmmewe) |
| *Bactris* | Arecaceae | Arecales | 10.15468/dl.p7yyab | [10.15468/dl.v9xr7g](https://doi.org/10.15468/dl.v9xr7g) |
| *Calophyllum* | Calophyllaceae | Malpighiales | 10.15468/dl.bs525w | [10.15468/dl.umzv2d](https://doi.org/10.15468/dl.umzv2d) |
| *Canthium* | Rubiaceae | Gentianales | 10.15468/dl.uy4ewd | [10.15468/dl.63n479](https://doi.org/10.15468/dl.63n479) |
| *Capparis* | Capparaceae | Brassicales | 10.15468/dl.m9b9km | [10.15468/dl.shqste](https://doi.org/10.15468/dl.shqste) |
| *Casearia* | Salicaceae | Malpighiales | 10.15468/dl.9vhcb3 | [10.15468/dl.bnkmhh](https://doi.org/10.15468/dl.bnkmhh) |
| *Celtis* | Cannabaceae | Rosales | 10.15468/dl.e9pdqx | [10.15468/dl.j5rs9a](https://doi.org/10.15468/dl.j5rs9a) |
| *Chrysophyllum* | Sapotaceae | Ericales | 10.15468/dl.vy6zcs | doi.org/10.15468/dl.856zkg |
| *Cissus* | Vitaceae | Vitales | 10.15468/dl.5zamzm | 10.15468/dl.46vjdp |
| *Clidemia* | Melastomataceae | Myrtales | 10.15468/dl.np43k8 | [10.15468/dl.g4dbqm](https://doi.org/10.15468/dl.g4dbqm) |
| *Cordia* | Cordiaceae | Boraginales | 10.15468/dl.82vw5k | [10.15468/dl.vgvtdd](https://doi.org/10.15468/dl.vgvtdd) |
| *Crataegus* | Rosaceae | Rosales | 10.15468/dl.am6f2x | 10.15468/dl.jc7nv3 |
| *Cryptocarya* | Lauraceae | Laurales | 10.15468/dl.v9mtuf | 10.15468/dl.ps9cg7 |
| *Cyphostemma* | Vitaceae | Vitales | 10.15468/dl.74kgya | [10.15468/dl.qzgrk5](https://doi.org/10.15468/dl.qzgrk5) |
| *Diospyros* | Ebenaceae | Ericales | 10.15468/dl.mk277t | 10.15468/dl.68vtew |
| *Elaeocarpus* | Elaeocarpaceae | Oxidales | 10.15468/dl.cnnw9p | 10.15468/dl.fc5nqv |
| *Endiandra* | Lauraceae | Laurales | 10.15468/dl.j7sz5p | 10.15468/dl.6cwm2w |
| *Erythroxylum* | Erythroxylaceae | Malpighiales | 10.15468/dl.qpmbhc |  |
| *Garcinia* | Clusiaceae | Malpighiales | 10.15468/dl.w2t6tu | 10.15468/dl.s6mrxm |
| *Grewia* | Malvaceae | Malvales | 10.15468/dl.ajtdad | doi.org/10.15468/dl.arcvbe |
| *Ilex* | Aquifoliaceae | Aquifoliales | 10.15468/dl.un2339 | doi.org/10.15468/dl.qtkryu |
| *Lonicera* | Caprifoliaceae | Dipsacales | 10.15468/dl.yv75tm | 10.15468/dl.8rc3wg |
| *Maytenus* | Celastraceae | Celastrales | 10.15468/dl.7kudew | 10.15468/dl.5acpwv |
| *Nectandra* | Lauraceae | Laurales | 10.15468/dl.cmy59m | 10.15468/dl.k9fru2 |
| *Ochna* | Ochnaceae | Malpighiales | 10.15468/dl.9byy59 | 10.15468/dl.9xjbjn |
| *Ocotea* | Lauraceae | Laurales | 10.15468/dl.ja3sv3 | 10.15468/dl.zdbgzm |
| *Passiflora* | Passifloraceae | Malpighiales | 10.15468/dl.hccnav | 10.15468/dl.675vsp |
| *Pavetta* | Rubiaceae | Gentianales | 10.15468/dl.qr8j4d | 10.15468/dl.phdchj |
| *Pouteria* | Sapotaceae | Ericales | 10.15468/dl.85gsak | 10.15468/dl.kkfqph |
| *Protium* | Burseraceae | Sapindales | 10.15468/dl.cqmwm6 | 10.15468/dl.dtfeex |
| *Prunus* | Rosaceae | Rosales | 10.15468/dl.4ddak8 | 10.15468/dl.6esbj5 |
| *Rhamnus* | Rhamnaceae | Rosales | 10.15468/dl.sngcd8 | 10.15468/dl.kntef9 |
| *Rhus* | Anacardiaceae | Sapindales | 10.15468/dl.x2r62e | 10.15468/dl.jrkt7y |
| *Ribes* | Grossulariaceae | Saxifragales | 10.15468/dl.e8yam6 | 10.15468/dl.yrg4ea |
| *Rosa* | Rosaceae | Rosales | 10.15468/dl.sysj8r | 10.15468/dl.q4qnzx |
| *Rubus* | Rosaceae | Rosales | 10.15468/dl.g3phpa | 10.15468/dl.99u2vy |
| *Smilax* | Smilacaceae | Liliales | 10.15468/dl.mhm5r5 | 10.15468/dl.pvqfxx |
| *Solanum* | Solanaceae | Solanales | 10.15468/dl.u3unmy | [10.15468/dl.mwut9k](https://doi.org/10.15468/dl.mwut9k) |
| *Sorbus* | Rosaceae | Rosales | 10.15468/dl.yhqed8 | 10.15468/dl.3r65jz |
| *Strychnos* | Loganiaceae | Gentianales | 10.15468/dl.pvh2bw | 10.15468/dl.bje3cq |
| *Symplocos* | Symplocaceae | Ericales | 10.15468/dl.f6dq2g | 10.15468/dl.pyxe3b |
| *Terminalia* | Combretaceae | Myrtales | 10.15468/dl.c2vpkd | 10.15468/dl.6cxpdw |
| *Tricalysia* | Rubiaceae | Gentianales | 10.15468/dl.4txbx5 | 10.15468/dl.4fa527 |
| *Vaccinium* | Ericaceae | Ericales | 10.15468/dl.vamd28 | 10.15468/dl.duqy34 |
| *Viburnum* | Viburnaceae | Dipsacales | 10.15468/dl.7t6nqj | 10.15468/dl.96t3rj |
| *Vitex* | Vitaceae | Vitales | 10.15468/dl.zr6858 | 10.15468/dl.knyt8s |
| *Iochroma* | Solanaceae | Solanales | 10.15468/dl.hpv6gg |  |
| *Gaultheria* | Ericaceae | Ericales | 10.15468/dl.af2m85 |  |
| *Cornus* | Cornaceae | Cornales | 10.15468/dl.zdh594 |  |
| *Scaevola* | Goodeniaceae | Asterales | 10.15468/dl.ey3zub |  |

**Table S3. Number of species per color category** and percentage of species per color category. The upper section of the table breaks down species numbers for flowers in more refined (11) categories where light green (G1), light pink (P1), light purple (Pu1), light red (R1), and light yellow (Y1) are indicated separately; the second “main” section of the table lumps colors into their respective main category (green, orange, pink, purple, red, white, yellow, blue); the final section of the table shows the categorization as used in this study, summarizing colors into eight categories and merging light colors with white, following common practice in ecogeographic analyses of color using categories, i.e., compare Delmas et al. 2020).

| **Fine color** | **Number of species** | | **Percentage of species** | |
| --- | --- | --- | --- | --- |
| **category** | **Flowers** | **Fruits** | **Flowers** | **Fruits** |
| blue | 5 |  | 0.20 |  |
| brown | 11 |  | 0.44 |  |
| green | 106 |  | 4.22 |  |
| light green | 238 |  | 9.47 |  |
| light pink | 72 |  | 2.86 |  |
| light purple | 49 |  | 1.95 |  |
| light yellow | 296 |  | 11.77 |  |
| light red | 14 |  | 0.56 |  |
| orange | 28 |  | 1.11 |  |
| pink | 122 |  | 4.85 |  |
| purple | 234 |  | 9.31 |  |
| red | 92 |  | 3.66 |  |
| white | 1384 |  | 55.05 |  |
| yellow | 175 |  | 6.96 |  |
|  |  |  |  |  |
| **Main color** |  |  |  |  |
| **category** | **Flowers** |  | **Flowers** |  |
| green | 345 |  | 12.33 |  |
| orange | 29 |  | 1.04 |  |
| pink | 196 |  | 7.00 |  |
| purple | 284 |  | 10.15 |  |
| red | 112 |  | 4.00 |  |
| white | 1384 |  | 49.45 |  |
| yellow | 471 |  | 16.83 |  |
| blue | 5 |  | 0.18 |  |
|  |  |  |  |  |
| **Final color** |  |  |  |  |
| **category** | **Flowers** | **Fruits** | **Flowers** | **Fruits** |
| black | 9 | 605 | 0.36 | 24.07 |
| blue |  | 338 |  | 13.44 |
| brown |  | 76 |  | 3.02 |
| green | 108 | 379 | 4.30 | 15.08 |
| orange | 20 | 179 | 0.80 | 7.12 |
| pink | 122 |  | 4.85 |  |
| purple | 237 |  | 9.43 |  |
| red | 93 | 868 | 3.70 | 34.53 |
| white | 2053 | 83 | 81.66 | 3.30 |
| yellow | 172 | 286 | 6.84 | 11.38 |

**Table S4. Parameter optimization in BRT models on flower and fruit color diversity and difference in diversity.** We varied the learning rate (lr) between 0.01 to 0.001, the tree complexity (tc) from 1 to 5 (in full integer intervals) and the bag fraction (bf) between 0.5 and 0.75; differences in deviance were overall small, and the correlation between training and testing data in the ten-fold cross-validation varied between approximately 50% and 70%. Deviance and correlation were similar when using all grid cells with a minimum of three species, and grid cells containing a minimum of ten species.


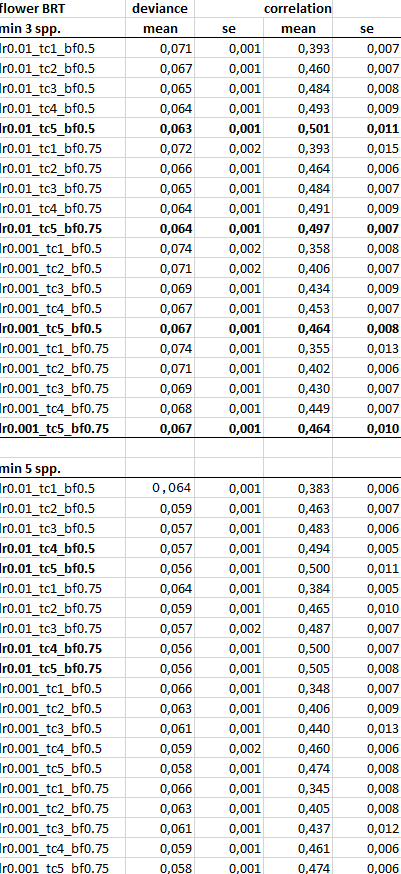


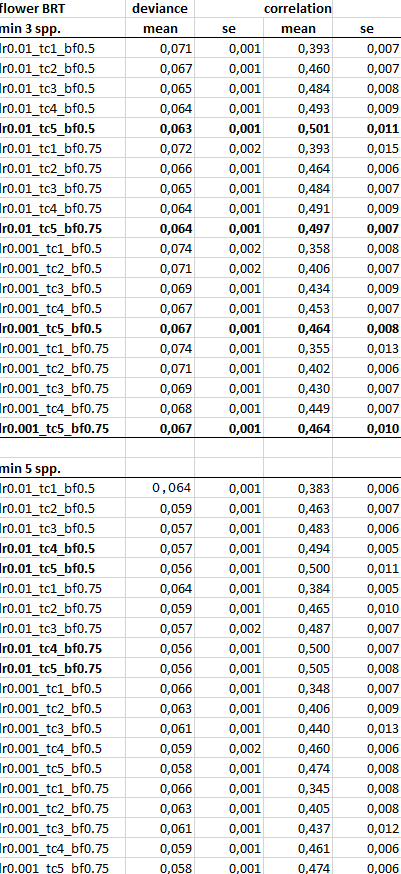


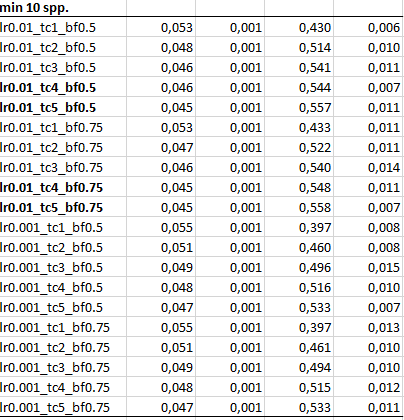


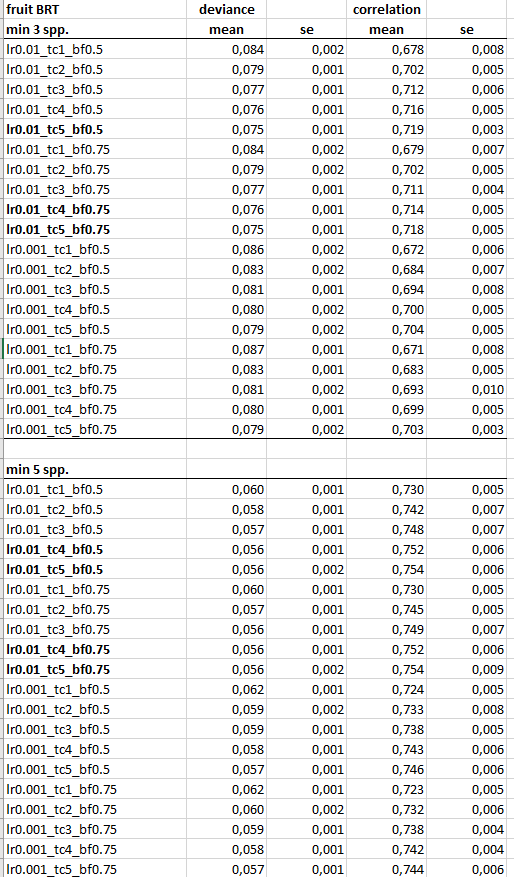


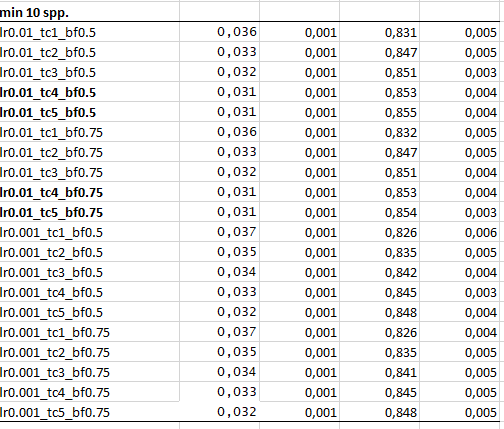


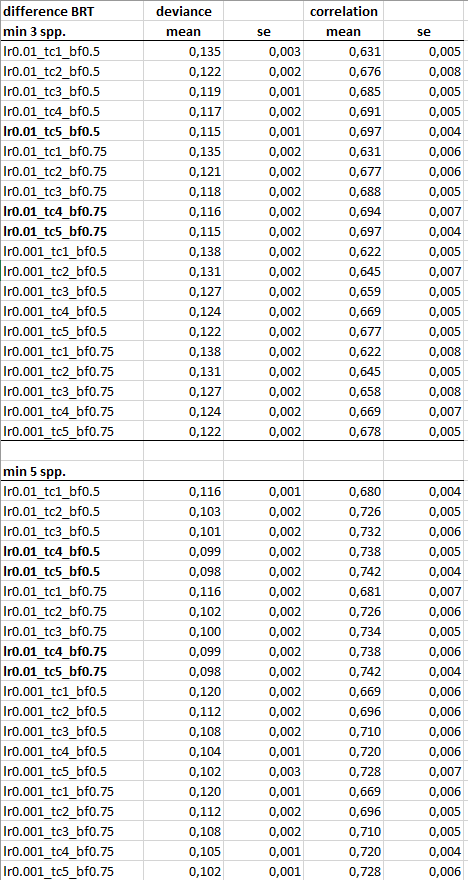


**
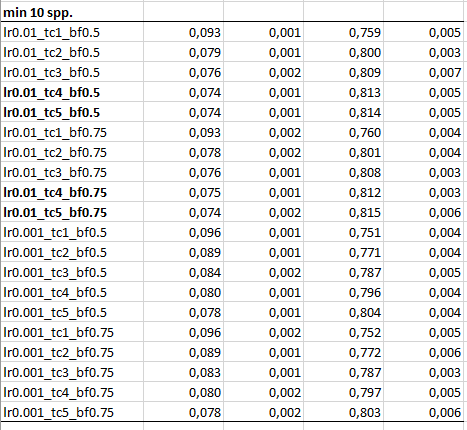
**

**Table S5. Model selection for multinomial regression models on flower and fruit color categories**, mean annual temperature (bio1), aridity index (AI) and UV-B irradiance. Models are based on mean climatic variables per species; clade was used as a categorical grouping variable.


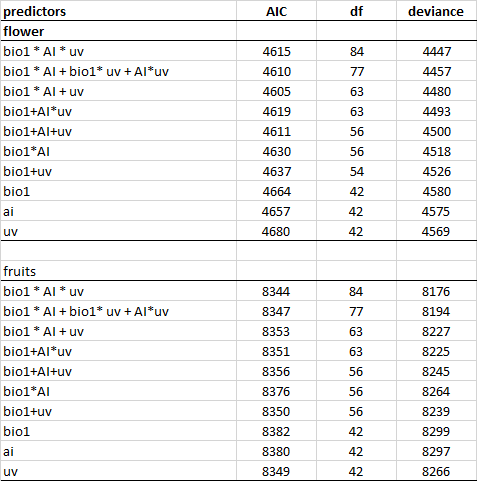


**Table S6. Pairwise comparison (*z*-values) of flower color diversity (lower diagonal) and fruit color diversity (upper diagonal)** **among biomes**. Comparisons that were identified as significantly different through Dunn’s tests are highlighted in gray; note the strong differentiation in fruit color diversity among tropical and temperate biomes.


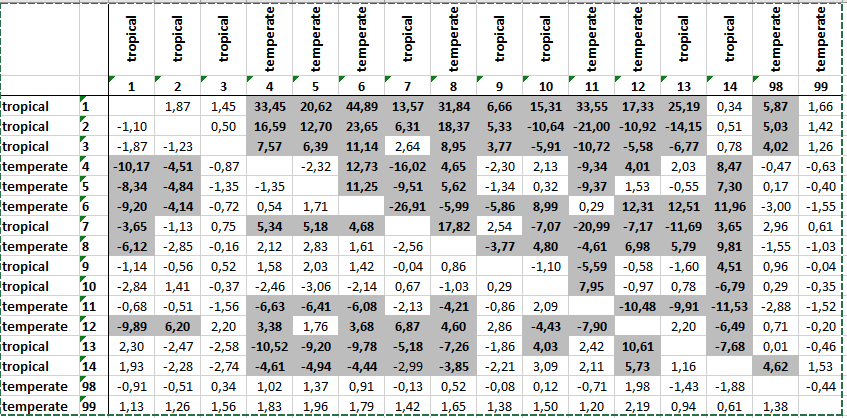


**Table S7. Relative influence of the environmental variables mean annual temperature, aridity, UV-B, and phylogenetic diversity on flower and fruit color diversity**, and difference between flower and fruit color diversity, across the BRT models with best parameter settings (lowest predictive deviance) for a minimum of three and ten species, and training data sets consisting of 50% or 75% of data. Abbreviations: lr, learning rate; tc, tree complexity; bf, bag fraction. Phylogenetic diversity was included as the average mean phylogenetic distance per grid cell.


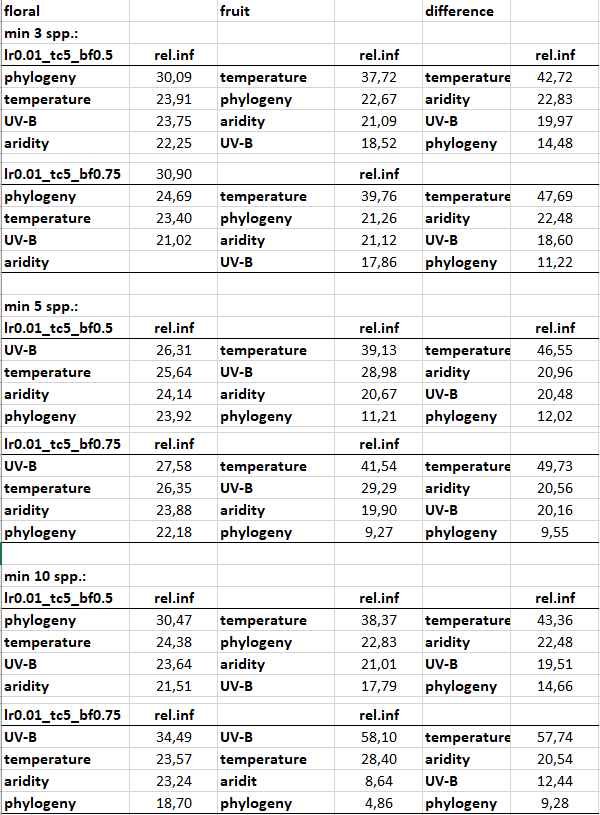


**Table S8. Interactions between variables included in BRT models** for flower and fruit color diversity and difference in color diversity (Fig. S9).


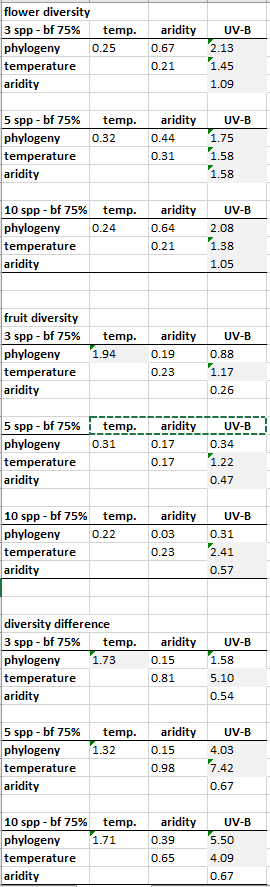


**Table S9. Comparison of *χ*² residuals shows different patterns in the frequency of different flower and fruit colors among biomes**. Negative values indicate that a color type is less common than expected by chance; positive values indicate that a color type is more common than expected by chance. Significant values are in bold. Biomes are grouped into general “types” to summarize general patterns (tropical forest, temperate forest, tropical grassland, temperate grassland, montane, xeric, lakes, Arctic). B indicates the biome by number: 1, tropical and subtropical moist broadleaf forests; 2, tropical and subtropical dry broadleaf forests; 3, tropical and subtropical coniferous forests; 4, temperate broadleaf and mixed forests; 5, temperate coniferous forests; 6, boreal forests, taiga; 7, tropical and subtropical grasslands, savannas and shrublands; 8, temperate grasslands, savannas and shrublands; 9, flooded grasslands, savannas and shrublands; 10, montane grasslands, savannas and shrublands; 11, tundra; 12, Mediterranean forests, woodlands, and scrub or sclerophyll forests; 13, deserts and xeric shrublands; 14, mangroves; 98, lakes; 99, Arctic (rock and ice/Greenland).

| **Flower** |  |  |  |  | |  | |  | |  | |  | |  | |
| --- | --- | --- | --- | --- | --- | --- | --- | --- | --- | --- | --- | --- | --- | --- | --- |
| **type** | **B** | **Black** | **Green** | **Orange** | | **Pink** | | **Purple** | | **Red** | | **White** | | **Yellow** | |
| **tropical forest** | **1** | **-6.08** | **18.43** | **3.93** | **-58.05** | | **-18.16** | | **-16.26** | | **34.74** | | **17.01** | |  |
| **tropical forest** | **2** | **-3.62** | 1.17 | -0.53 | **-18.85** | | **7.60** | | **-2.28** | | **5.38** | | **5.76** | |  |
| **tropical forest** | **3** | -2.48 | 2.72 | **-3.98** | **-3.60** | | **-6.12** | | **-5.55** | | **7.68** | | -0.40 | |  |
| **temperate forest** | **4** | **-4.28** | 1.31 | **-10.11** | **11.45** | | **-25.15** | | **10.71** | | **11.68** | | **-16.29** | |  |
| **temperate forest** | **5** | **12.50** | **-5.87** | **4.60** | **14.82** | | **-23.17** | | **8.52** | | 0.30 | | -1.21 | |  |
| **temperate forest** | **6** | **-4.49** | **-20.51** | **-9.25** | **93.12** | | **-21.71** | | **29.51** | | **-34.85** | | **-22.90** | |  |
| **tropical grassland** | **7** | **4.87** | **5.07** | **5.37** | **-29.96** | | **41.32** | | **-9.88** | | **-5.98** | | **6.46** | |  |
| **temperate grassland** | **8** | 0.08 | **8.93** | **-5.88** | **6.93** | | **7.20** | | **-3.35** | | **-10.71** | | **3.10** | |  |
| **tropical grassland** | **9** | -1.49 | -1.39 | 1.90 | -1.43 | | -0.01 | | -1.56 | | 0.95 | | 1.88 | |  |
| **montane** | **10** | **20.12** | **-3.58** | -1.29 | 2.58 | | **13.98** | | -1.67 | | **-8.94** | | 0.58 | |  |
| **temperate grassland** | **11** | -3.13 | **-11.60** | **-4.65** | **67.83** | | **-14.67** | | 2.84 | | **-20.64** | | **-15.26** | |  |
| **xeric** | **12** | 1.60 | **-9.43** | **27.18** | **-4.32** | | **22.76** | | -0.82 | | **-11.40** | | 3.36 | |  |
| **xeric** | **13** | -1.63 | **-10.38** | -0.51 | **-20.47** | | **69.59** | | **-12.42** | | **-22.81** | | **17.18** | |  |
| **tropical** | **14** | -0.52 | -0.25 | -0.54 | **-8.87** | | **-2.86** | | 0.57 | | **5.86** | | 1.8**3** | |  |
| **lakes** | **98** | -0.99 | 1.41 | -1.47 | 3.93 | | -2.59 | | -0.81 | | 0.36 | | -2.44 | |  |
| **arctic** | **99** | 3.30 | -1.04 | -0.42 | 3.04 | | -1.36 | | 2.76 | | -1.44 | | -1.37 | |  |
|  |  |  |  |  |  | |  | |  | |  | |  | |  |
| **fruits** |  |  |  |  |  | |  | |  | |  | |  | |  |
| **type** | **B** | **black** | **blue** | **brown** | **green** | | **orange** | | **red** | | **white** | | **yellow** | |  |
| **tropical forest** | **1** | -1.71 | **9.55** | **13.58** | **65.48** | | **36.42** | | **-75.38** | | **-7.95** | | **42.51** | |  |
| **tropical forest** | **2** | 0.73 | **-8.35** | **19.39** | **22.23** | | **19.44** | | **-29.45** | | **10.01** | | **9.42** | |  |
| **tropical forest** | **3** | **13.70** | 1.15 | -3.08 | **9.51** | | **8.24** | | **-16.92** | | -3.21 | | -2.96 | |  |
| **temperate forest** | **4** | **15.25** | -0.71 | **-17.21** | **-53.23** | | **-35.26** | | **65.86** | | -0.25 | | **-59.39** | |  |
| **temperate forest** | **5** | **15.24** | **14.54** | **-14.94** | **-26.85** | | **-17.76** | | **17.64** | | **4.37** | | **-29.08** | |  |
| **temperate forest** | **6** | **-21.31** | -1.36 | **-19.58** | **-31.63** | | **-25.84** | | **58.23** | | **4.84** | | **-15.00** | |  |
| **tropical grassland** | **7** | **-8.45** | **-18.82** | **6.16** | **25.37** | | **28.00** | | **-32.19** | | **-6.75** | | **48.29** | |  |
| **temperate grassland** | **8** | 1.16 | -2.54 | -0.30 | **-16.89** | | **-12.37** | | **15.83** | | **11.27** | | **-11.46** | |  |
| **tropical grassland** | **9** | -0.01 | **-4.16** | 1.54 | -0.09 | | 2.46 | | -0.87 | | -2.51 | | **4.82** | |  |
| **montane** | **10** | **-9.95** | **4.04** | -0.46 | 1.29 | | **4.44** | | 3.09 | | -2.16 | | **3.72** | |  |
| **temperate grassland** | **11** | **-22.44** | **13.45** | **-9.97** | **-15.89** | | **-12.98** | | **28.15** | | **-9.78** | | **6.75** | |  |
| **xeric** | **12** | **12.85** | -2.11 | **15.82** | **-12.17** | | **-1.50** | | **4.34** | | **-8.09** | | **-17.53** | |  |
| **xeric** | **13** | **-10.17** | **-10.44** | **16.84** | **16.26** | | **6.57** | | **-25.22** | | **12.53** | | **34.54** | |  |
| **tropical** | **14** | **4.19** | -3.50 | **6.89** | **8.48** | | **4.03** | | **-13.41** | | 2.26 | | **5.17** | |  |
| **lakes** | **98** | -3.05 | 0.69 | -3.15 | -3.54 | | -2.07 | | **5.05** | | **5.42** | | -2.16 | |  |
| **arctic** | **99** | 0.08 | 0.11 | 0.25 | -1.42 | | -0.27 | | 1.32 | | -0.17 | | -1.18 | |  |

**Table S10. Parameters for multinomial regression model with lowest AIC** (Table S3), in flowers including an interaction between mean annual temperature (bio1) and aridity index (AI) and additive effects UV, in fruits including interactions between all three variables. Significant *P*-values are in bold. The last column shows the results of the randomization test (occurrences for each species drawn at random 100 times from the total dataset of more than 4 million occurrences), with the percentage of models identifying the respective model parameters as significant.

| **flowers** |  |  |  |  |  |
| --- | --- | --- | --- | --- | --- |
| **dark** | **Estimate** | **SE** | ***z*** | ***P*** | **Randomization <0.05** |
| (Intercept) | -6.0725 | 0.5788 | -10.491 | <2e-16 | 100 |
| **bio1m** | **-1.0539** | **0.4256** | **-2.476** | **0.0133** | **56** |
| aim | -0.6406 | 0.5659 | -1.132 | 0.2576 | 0 |
| uv | 0.3959 | 0.2986 | 1.326 | 0.1849 | 5 |
| bio1m:aim | -0.4281 | 0.3683 | -1.163 | 0.2450 | 7 |
|  |  |  |  |  |  |
| **green** |  |  |  |  |  |
| (Intercept) | -3.3350 | 0.3007 | -11.091 | <2e-16 | 100 |
| bio1m | -0.0335 | 0.1893 | -0.177 | 0.8595 | 89 |
| aim | -0.1027 | 0.1542 | -0.666 | 0.5054 | 2 |
| uv | 0.2841 | 0.1747 | 1.626 | 0.1039 | 23 |
| bio1m:aim | 0.2573 | 0.1514 | 1.699 | 0.0893 | 11 |
|  |  |  |  |  |  |
| **orange** |  |  |  |  |  |
| (Intercept) | -5.1270 | 0.3923 | -13.069 | <2e-16 | 100 |
| bio1m | 0.2470 | 0.4022 | 0.614 | 0.5391 | 0 |
| aim | -0.1369 | 0.3395 | -0.403 | 0.6868 | 5 |
| uv | -0.0533 | 0.3661 | -0.146 | 0.8842 | 0 |
| bio1m:aim | 0.5672 | 0.2972 | 1.909 | 0.0563 | 17 |
|  |  |  |  |  |  |
| **pink** |  |  |  |  |  |
| (Intercept) | -3.81238 | 0.29587 | -12.885 | <2e-16 | 100 |
| **bio1m** | **-0.90617** | **0.14378** | **-6.302** | **2.93e-10** | **100** |
| aim | 0.04738 | 0.13350 | 0.355 | 0.723 | 2 |
| **uv** | **0.54547** | **0.09934** | **5.491** | **4.00e-08** | **100** |
| bio1m:aim | 0.06432 | 0.10863 | 0.592 | 0.554 | 1 |
|  |  |  |  |  |  |
| **purple** |  |  |  |  |  |
| (Intercept) | -4.59394 | 0.35944 | -12.781 | <2e-16 | 100 |
| **bio1m** | **-0.45899** | **0.14606** | **-3.143** | **0.00167** | **82** |
| **aim** | **-0.53113** | **0.10979** | **-4.838** | **1.31e-06** | **3** |
| uv | 0.07072 | 0.11614 | 0.609 | 0.54258 | 0 |
| **bio1m:aim** | **-0.33532** | **0.12397** | **-2.705** | **0.00683** | **84** |
|  |  |  |  |  |  |
| **red** |  |  |  |  |  |
| (Intercept) | -3.72970 | 0.30697 | -12.150 | <2e-16 | 100 |
| bio1m | -0.14711 | 0.16140 | -0.911 | 0.3621 | 0 |
| aim | 0.01285 | 0.12603 | 0.102 | 0.9188 | 2 |
| **uv** | **0.29327** | **0.12634** | **2.321** | **0.0203** | **54** |
| bio1m:aim | -0.05598 | 0.10198 | -0.549 | 0.5830 | 2 |
|  |  |  |  |  |  |
| **yellow** |  |  |  |  |  |
| (Intercept) | -2.96261 | 0.28427 | -10.422 | <2e-16 | 100 |
| bio1m | -0.07213 | 0.14943 | -0.483 | 0.62934 | 4 |
| **aim** | **-0.31486** | **0.11959** | **-2.633** | **0.00847** | **61** |
| uv | 0.23604 | 0.12410 | 1.902 | 0.05717 | 56 |
| **bio1m:aim** | **0.25173** | **0.11385** | **2.211** | **0.02703** | **27** |

**fruits**

| **black** | **Estimate** | **std error** | **z-value** | **p-value** | **randomization <0.05** |
| --- | --- | --- | --- | --- | --- |
| (Intercept) | 1.10738 | 0.33515 | 3.304 | 0.000953 | 100 |
| bio1m | -0.08990 | 0.15876 | -0.566 | 0.571217 | 6 |
| aim | -0.04077 | 0.11666 | -0.349 | 0.726742 | 2 |
| **uv** | **-0.56912** | **0.15346** | **-3.709** | **0.000208** | **100** |
| bio1m:aim | -0.08364 | 0.12371 | -0.676 | 0.498985 | 3 |
| bio1m:uv | 0.20187 | 0.14306 | 1.411 | 0.158230 | 8 |
| aim:uv | -0.11459 | 0.15892 | -0.721 | 0.470874 | 2 |
|  |  |  |  |  |  |
| **blue** |  |  |  |  |  |
| (Intercept) | 0.37117 | 0.30914 | 1.201 | 0.2299 | 82 |
| bio1m | -0.28212 | 0.16410 | -1.719 | 0.0856 | 1 |
| aim | 0.02319 | 0.12289 | 0.189 | 0.8503 | 1 |
| **uv** | **-0.52575** | **0.16349** | **-3.216** | **0.0013** | **70** |
| bio1m:aim | 0.02706 | 0.12424 | 0.218 | 0.8276 | 1 |
| bio1m:uv | -0.02848 | 0.15189 | -0.187 | 0.8513 | 1 |
| aim:uv | -0.01820 | 0.15899 | -0.115 | 0.9088 | 1 |
|  |  |  |  |  |  |
| **brown** |  |  |  |  |  |
| (Intercept) | -1.2767 | 0.3543 | -3.603 | 0.000314 | 0 |
| bio1m | 0.6213 | 0.3516 | 1.767 | 0.077266 | 0 |
| aim | -0.3093 | 0.2433 | -1.271 | 0.203577 | 4 |
| **uv** | **-0.8575** | **0.3446** | **-2.488** | **0.012847** | **50** |
| **bio1m:aim** | **0.7354** | **0.3033** | **2.425** | **0.015313** | **21** |
| bio1m:uv | 0.1109 | 0.2369 | 0.468 | 0.639633 | 2 |
| aim:uv | -0.4283 | 0.3603 | -1.188 | 0.234642 | 2 |
|  |  |  |  |  |  |
| **orange** |  |  |  |  |  |
| (Intercept) | -0.28998 | 0.29797 | -0.973 | 0.3305 | 0 |
| bio1m | 0.09276 | 0.21313 | 0.435 | 0.6634 | 1 |
| aim | -0.08470 | 0.15975 | -0.530 | 0.5960 | 0 |
| uv | -0.16155 | 0.20912 | -0.772 | 0.4398 | 1 |
| bio1m:aim | 0.14953 | 0.16448 | 0.909 | 0.3633 | 1 |
| bio1m:uv | 0.14295 | 0.17915 | 0.798 | 0.4249 | 2 |
| **aim:uv** | **-0.41011** | **0.20563** | **-1.994** | **0.0461** | **17** |
|  |  |  |  |  |  |
| **red** |  |  |  |  |  |
| (Intercept) | 1.45702 | 0.30955 | 4.707 | 2.51e-06 | 100 |
| bio1m | -0.28712 | 0.15337 | -1.872 | 0.0612 | 74 |
| aim | 0.10915 | 0.11113 | 0.982 | 0.3260 | 13 |
| **uv** | **-0.37083** | **0.14684** | **-2.525** | **0.0116** | **100** |
| bio1m:aim | -0.06836 | 0.11232 | -0.609 | 0.5428 | 1 |
| **bio1m:uv** | **0.35077** | **0.13813** | **2.540** | **0.0111** | **65** |
| **aim:uv** | **-0.30086** | **0.14799** | **-2.033** | **0.0421** | **22** |
|  |  |  |  |  |  |
| **white** |  |  |  |  |  |
| (Intercept) | -1.03346 | 0.35721 | -2.893 | 0.00381 | 0 |
| bio1m | -0.45100 | 0.23799 | -1.895 | 0.05808 | 11 |
| aim | 0.08183 | 0.16205 | 0.505 | 0.61359 | 8 |
| uv | -0.40600 | 0.26303 | -1.544 | 0.12270 | 81 |
| bio1m:aim | -0.09394 | 0.18337 | -0.512 | 0.60843 | 1 |
| bio1m:uv | 0.14888 | 0.19483 | 0.764 | 0.44478 | 2 |
| aim:uv | -0.02922 | 0.23107 | -0.126 | 0.89936 | 0 |
|  |  |  |  |  |  |
| **yellow** |  |  |  |  |  |
| (Intercept) | -0.15097 | 0.27466 | -0.550 | 0.583 | 3 |
| bio1m | -0.04348 | 0.17558 | -0.248 | 0.804 | 5 |
| aim | -0.02676 | 0.13390 | -0.200 | 0.842 | 2 |
| uv | -0.18759 | 0.17635 | -1.064 | 0.287 | 51 |
| bio1m:aim | 0.06601 | 0.13432 | 0.491 | 0.623 | 1 |
| bio1m:uv | 0.24335 | 0.15815 | 1.539 | 0.124 | 10 |
| aim:uv | -0.23689 | 0.17582 | -1.347 | 0.178 | 7 |

**Supplementary methods**

**Flower–fruit color data set and phylogeny**

We based our study on the global data set on fruit colors from Sinnott-Armstrong et al. (2018). From this data set, we selected only clades that are animal-pollinated and animal-dispersed and for which at least 10% of the total species in the clade had been covered (from estimates of Christenhusz et al., 2017). We further added *Gaultheria*, *Cornus*, and *Scaevola*, yielding 51 clades across 40 plant families and 23 orders. For these clades, we extracted all taxa included in the phylogeny by Jin and Qian (2019), yielding a candidate list of 3250 taxa.

Fruit color data was readily available from Sinnott-Armstrong et al. (2018) for 1339 taxa, scored into eight categories (black, blue, red, white, green, yellow, orange, brown). For all remaining taxa, we scored fruit color into these categories using species descriptions, published data sets (Lu et al., 2019; Lindelof et al., 2020; Hilgenhof et al., 2023) and iNaturalist images. For flower colors, we searched for photos of each taxon on GBIF or iNaturalist and scored colors into 45 categories using a color reference chart (Figure S1). We only used images with appropriate light conditions. Table S3 summarizes the number of species we found for each of the 45 color categories. We are aware that scoring color, a quantitative trait, into categories is arbitrary regardless of the level of detail and care used in the categorization. Our approach of scoring color into 45 categories using a reference chart was an attempt of making the scoring as transparent as possible. To make the flower color scoring comparable to the fruit color scoring (eight categories), however, we had to summarize the flower colors into eight categories too. Table S3 represents the proportion of species in each of these eight categories. In brief, we summarized all very light shades (denoted with “1” in the color reference chart in Figure S1) into white, together with species scored as “white”, following common practice of studies using color categorization (Delmas et al. 2020), while all primary colors scored as “2”, “3” and “4” were summarized as primary colors (blue, green, orange, pink, purple, red, yellow), and colors scored with “5” were summarized as “dark” flowers. In addition, we present two different categorizations for flower colors, one where we scored flower colors into 11 categories, keeping light colors (denoted with “1”) separate where present in higher numbers (green, pink, purple, yellow). And second, we lumped all colors into their main category irrespective of color shades, denoted as “main” in Table S3. While we believe that our eight-category scheme, with light colors summarized as “white”, is most meaningful from a functional perspective. To make our study immediately comparable to other works using colors categorically (Delmas et al. 2020), we also emphasize that ideally, follow-up studies should attempt to measure flower colors quantitatively (spectral data) to make objective analyses tangible in the future.

**Collation and cleaning of occurrence data**

To obtain occurrence records, we submitted the list of 2890 taxa to the GBIF portal (May 2023) through the rgbif interface (Chamberlain et al., 2023), downloaded the data and retained all georeferenced records. Next, we used custom filtering techniques implemented in CoordinateCleaner (Zizka et al., 2019) to remove occurrences at country centroids, in the sea, around GBIF headquarters, duplicates, zero coordinates, country mismatches and coordinates from capital cities. We also screened for synonyms using TaxonStand (Zhang & Qiang 2023) and retained all records that matched the taxa of Jin and Qian (2019). To filter out potential invasive ranges, we next submitted the taxa list to the GloNAF database (vanKleunen et al., 2019). We identified 314 taxa as invasive in a part of their range and removed the occurrences in the respective invasive ranges (9,338,392 records removed). After these filtering steps, our list included 2853 taxa with georeferenced localities.

In the next cleaning step, we plotted range maps for each of the 51 clades and compared clade ranges to range information available on the Plants of the World Online database (<https://powo.science.kew.org/>, accessed August 2023). We manually removed occurrences outside the documented ranges of the respective clades and checked ranges of species commonly used in cultivation, then removed occurrences in cultivated ranges. For large clades with occurrences in the United States, Europe, and Australia, we also assessed species for these continents to verify that these lists did not include non-native species on either continent. The reason behind this screening step is that herbaria on these continents often house species from other regions of the world, which had not been removed by the initial cleaning steps in CoordinateCleaner. We further repeated filtering against country centroids (1 km buffer; 70 records removed), capital cities (10 km buffer; 579 records removed), and herbaria (1 km buffer; 71,067 records removed) with CoordinateCleaner, and removed coordinates lacking decimal degrees (14,097 records removed) or representing duplicates (2,421,865), reducing the dataset to 6,169,815 records across 2815 taxa (details in Table S1). We subsampled this data set to only include one occurrence per species per 30 arc-sec grid cell for further processing of the data set (4,757,769 records across 2815 taxa).

As a final assessment of the accuracy of our records, we mapped patterns of species richness onto a world map (rworldmap, South, 2011). To this end, we first subsampled occurrences to one occurrence per species per 1 x 1 degree grid cell (ca. 110 kilometers at the equator) to reduce bias of uneven collection efforts (dismo, Hijmans et al., 2023; raster, Hijmans, 2023), yielding 12,508 (of 62,640) grid cells with a minimum of one occurrence record, with 214,319 records retained across all grid cells. Next, we calculated species richness per grid cell using the function RichnessGrid (speciesgeocodeR, Topel et al., 2016; Zizka and Antonelli, 2016). We manually checked species lists in all 14 grid cells with more than 180 records to assure that these did not pertain to institution records but represent true centers of biodiversity. For analyses of diversity patterns, we removed grid cells containing less than three species, leaving 210,935 occurrences across 10,097 grid cells and 2811 taxa.

**Extraction of WWF biomes and climatic variables**

To explore whether global patterns in flower and fruit color are associated with different environments, we downloaded the global shapefile for WWF biomes (Olson et al., 2001) and extracted the biome category for each occurrence point (over, sf, Pebesma et al., 2023). To test whether the distribution of flower and fruit colors is explained by temperature, aridity, or UV-B radiation, we next downloaded data on mean annual temperature (°C) from the WorldClim database at 30 arc-sec resolution (ca. 1 km at the equator; Fick and Hijmans, 2017). For aridity, we retrieved data on the global aridity index (AI) from Zomer et al. (2022), which is calculated as mean annual precipitation divided by mean annual reference evapo-transpiration (Trabucco and Zomer, 2018). The AI is a measure of potential vegetation growth and hence a good estimate for closed versus open canopy biomes, thus capturing potential drought and light stress relevant for our study. A higher AI indicates more mesic biomes (Trabucco and Zomer, 2018). For UV-B, we downloaded data on mean annual global UV-B radiation (15 arc-min resolution) from Beckmann et al. (2014). We extracted all climatic variables for each species’ occurrences in the 1 x 1 km grid (4,757,769 records across 2815 taxa).

**Calculation of grid-level climatic variables and genetic diversity**

Next, for each of the 10,097 1 x 1 degree grid cells containing a minimum of three species, we calculated mean climatic variables based on the climatic values extracted for each species’ occurrences in the 1 x 1 km grid. We removed 20 grid cells with missing data, leaving 10,077 grid cells. To reflect differences in phylogenetic diversity among communities, we calculated the mean phylogenetic distance (MPD, based on the pairwise cophenetic branch lengths distances of all taxa in the grid cell) for each of the 10077 grid cells using the mpd function in picante (Kembel et al., 2010).

**Global patterns of Shannon diversity index for flower and fruit colors**

For the 10,077 grid cells containing a minimum of three species, we calculated Shannon diversity indices for flower and fruit colors. We further calculated the difference between the two diversity indices by subtracting fruit from flower color diversity, meaning that a resulting value of 0 indicates no difference in Shannon diversity values for flower and fruit color, a positive value indicating higher flower color diversity, and a negative value indicating higher fruit color diversity. We visualized the distribution of flower and fruit color diversity and the difference in color diversity by plotting the diversity indices onto global maps.

Next, to test whether biomes differ in color diversity, we ran Kruskal–Wallis ANOVAs (we chose these nonparametric tests due to uneven variance among biomes). We used post hoc Dunn tests with a Bonferroni correction to account for multiple comparisons. We further assessed correlations between flower and fruit color diversity, species richness, and clade richness per grid cell.

Finally, to test whether the abiotic environment and/or phylogenetic diversity explain flower and fruit color diversity, we employed boosted regression trees (BRT), a powerful, machine-learning based ensemble method (Elith et al., 2008). The BRT are built through regression trees, where a large number of relatively simple regression models are fitted by recursively splitting the data space into the most homogeneous partitions. These trees are then combined in an additive, stagewise manner (existing trees are left unchanged as new ones are fitted) to improve the predictive power by minimizing the loss function (i.e., minimizing loss in predictive accuracy due to suboptimal models, estimated through changes in model deviance; Elith et al., 2008). The particular strength of machine learning methods such as BRT lies in their higher predictive accuracy compared to traditional (single best-fit) regression models, and in being particularly suited for (often skewed) ecological data since they are nonparametric, can handle outliers and correlated predictors (i.e., spatial autocorrelation), fit complex non-linear relationships between predictors, and automatically handle interaction effects (Cai et al., 2023). We here employed the gbm.step function (R-package dismo) to fit BRT models through 10-fold cross-validation (data is split into 10 subsets), using flower color, fruit color, or color difference as response and the abiotic environmental variables mean annual temperature, aridity index, UV-B irradiance and phylogenetic diversity per grid cell as predictors. We ran models for grid cells with a minimum of 3 (10,097 grid cells), 5 (8514) and 10 taxa (5979 data points) to assess potential bias in diversity estimates related to low species numbers per grid cells (compare Figure S2; Sabatini et al., 2022). Four parameters are of particular importance in fitting BRT: (i) the learning rate, which determines the contribution of a single tree to the growing model (with lower learning rates generally giving better model fit); (ii) the tree complexity, which determines the types of interactions allowed; (iii) the number of trees required for an optimal model (determined by i and ii), and (iv) the bag fraction, which controls the proportion of data used for training, and hence allowing for stochasticity in the model. Following general recommendations of Elith et al. (2008), we fit an initial model of tree complexity 5, a learning rate of 0.01, a bag fraction of 0.5, and a Gaussian family distribution. Next, we systematically varied the parameters by gradually reducing the tree complexity to 1 (to avoid overfitting of models), reducing the learning rate to 0.001, and increasing the bag fraction to 0.75 (Tables S6, S7). We used the cross-validation statistics to evaluate model performance and to choose model parameters with minimal predictive deviance. Since models with a learning rate of 0.01 gave a lower predictive deviance than when using a learning rate of 0.001, and still fit more than 5000 trees, hence conferring robustness, we proceeded with these models. Generally, models with a learning rate of 0.01, tree complexity of 5, and bag fraction of 75% had the lowest predictive deviance. Next, we explored whether the relative importance of predictors changes across different parameter settings. Since models based on grid cells including a minimum of three species sometimes gave different results than models based on a minimum of 5 or 10 species (Table S8), we report results for all data sets. We also tested whether simplifying models by removing predictors improved model fit (reducing predictive deviance) using the gbm.simplify function. Finally, we used the function gbm.interactions to test whether interactions were detected among predictors.

**Distribution of distinct flower and fruit colors**

To explore the global distribution of distinct colors, we calculated 1 x 1 degree grid-cell species richness for each flower and fruit color category separately. We scaled each color’s grid cell’s species richness by the corresponding grid cell’s total species richness (Fig. S3), thereby creating a proportional index of color richness (1, all species in the cell of a certain color; 0, no species in the cell of a certain color). We visualized these patterns by plotting the scaled color richness grids as well as the most dominant color per grid cell onto global maps. We further split the data set into the different WWF biomes and visualized the relative color composition of flowers and fruits in each biome using bar plots. We repeated this step for the different flower color categorization schemes (Table S3) to assess potential caveats of color categorization. Finally, we tested whether the abundance of a specific color within a biome differs significantly from random expectations using *χ*² tests.

To explicitly test whether temperature, aridity, or UV-B irradiance explain differences in the distribution of flower and fruit colors, we used multinomial logistic regressions. We treated flower or fruit color category as response variable, specifying “white” as reference for flowers (the most common color) and “green” as reference for fruits (since most fruits start out as green, see Sinnott-Armstrong et al., 2018). We included plant clade as a random effect grouping variable to account for phylogenetic non-independence. We started with a model including an interaction between all climatic variables and compared this model using AIC against models including additive effects or single variables only (Table S3). We fit models using the mblogit function (mclogit; Elff, 2022) and extracted estimated marginal means for the fitted models to plot the effects of climatic variables on flower and fruit colors (Lenth, 2023). We ran initial models averaging each climatic variable across the ooccurrences of each species and then repeated analyses 100 times by randomly selecting a single occurrence and its associated climatic variables per species, thereby alleviating bias through mid-domain effects stemming from averaged variables. We evaluated whether models based on species averages gave the same results as models using single occurrences.

**References**

Cai L, Kreft H, Taylor A, Denelle P, Schrader J, Essl F, et al. 2023. Global models and predictions of plant diversity based on advanced machine learning techniques. *New Phytologist* 237: 1432-1445.

Chamberlain S, Barve V, Mcglinn D, Oldoni D, Desmet P, Geffert L, Ram K. 2023. rgbif: Interface to the Global Biodiversity Information Facility API. Website: <https://CRAN.R-project.org/package=rgbif>

Delmas CEL, Kooyman RM, Rossetto M. 2020. Evolutionary constraints and adaptation shape the size and colour of rain forest fruits and flowers at continental scale. *Global Ecology and Biogeogr*aphy 29: 830–841.

Elith J, Leathwick JR, Hastie T. 2008. A working guide to boosted regression trees. *Journal of Animal Ecology* 77: 802-813.

Fick SE, Hijmans RJ. 2017. WorldClim 2: new 1km spatial resolution climate surfaces for global land areas. [*International Journal of Climatology* 37: 4302-4315](https://rmets.onlinelibrary.wiley.com/doi/abs/10.1002/joc.5086).

Hijmans RJ, Phillips S, Leathwick J, Elith J. 2023. dismo: Species distribution modeling. R package version 1.3-14, [https://CRAN.R-project.org/package=dismo](https://cran.r-project.org/package=dismo)

Hilgenhof R, Gagnon E, Knapp S, Aubriot X, Tepe EJ, Bohs L, Giacomin L, Gouvêa YF, Martine CT, Orejuela A, Orozco CI, Peralta IE, Särkinen T. 2023. Morphological trait evolution in *Solanum* (Solanaceae): Evolutionary lability of key taxonomic characters. *TAXON* 72: 811-847.

Jin Y, Qian H. 2019. V.PhyloMaker: an R package that can generate very large phylogenies for vascular plants. *Ecography* 42: 1353-1359.

Kembel SW, Cowan PD, Helmus MR, Cornwell WK, Morlon H, Ackerly DD, Blomberg SP, Webb CO. 2010. picante: R tools for integrating phylogenies and ecology. Bioinformatics 26: 1463–1464.

Lenth R (2023). emmeans: Estimated marginal means, aka least-squares means. R package version 1.10.7. Website https://cran.r-project.org/web/packages/emmeans/emmeans.pdf.

Lindelof, K., Lindo, J.A., Zhou, W., Ji, X. and Xiang, Q.-Y. (2020). Phylogenomics, biogeography, and evolution of the blue- or white-fruited dogwoods (*Cornus*)—Insights into morphological and ecological niche divergence following intercontinental geographic isolation. *Journal of Systematics and Evolution* 58, 604-645.

Lu L, Fritsch PW, Matzke NJ, Wang, H., Kron, K.A., Li, D.Z., Wiens, J.J. 2019. Why is fruit colour so variable? Phylogenetic analyses reveal relationships between fruit-colour evolution, biogeography and diversification. *Global Ecology and Biogeogr*aphy 28: 891–903.

Olson DM, Dinerstein E, Wikramanayake ED, Burgess ND, Powell GVN, Underwood EC, et al. 2001. Terrestrial ecoregions of the world: a new map of life on Earth. *BioScience* 51: 933-938.

Pebesma E, Bivand R (2023). Spatial data science: with applications in R. Chapman and Hall/CRC Press, Boca Raton, FL, USA. [doi:10.1201/9780429459016](https://doi.org/10.1201/9780429459016), <https://r-spatial.org/book/>.

Sabatini FM, Jiménez-Alfaro B, Jandt U, Chystrý M, Field R, Kessler M. 2022. Global patterns of vascular plant alpha diversity. *Nature Communications* **13**: 4683.

Sinnott-Armstrong MA, Downie AE, Federman S, Valido A, Jordano P, Donoghue MJ. 2018. Global geographic patterns in the colours and sizes of animal-dispersed fruits. *Global Ecology and Biogeogr*aphy, 27: 1339–1351.

South A. 2011. rworldmap: A new R package for mapping global data. *R Journal* 3: 35-43.

Stournaras KE, Lo E, Böhning-Gaese K, Cazetta E, Matthias Dehling D, Schleuning M, Stoddard MC, Donoghue MJ, Prum RO, Martin Schaefer H. 2013. How colorful are fruits? Limited color diversity in fleshy fruits on local and global scales. *New Phytologist* 198:617-629. doi: 10.1111/nph.12157.

Töpel M, Zizka A, Calió MF, Scharn R, Silvestro D, Antonelli A. 2017. SpeciesGeoCoder: Fast categorization of species occurrences for analyses of biodiversity, biogeography, ecology, and evolution. Systematic Biology 66: 145–151.

Trabucco A, Zomer R. 2018. Global aridity index and global potential evapo-transpiration geospatial database. CGIAR-CSI GeoPortal. <https://cgiarcsi.community/data/global-aridity-and-pet-database/>. Accessed 23 May 2023

van Kleunen M, Pyšek P, Dawson W, Essl F, Kreft H, Pergl J, et al. 2019. The global naturalized alien flora (GloNAF) database. *Ecology* 100: 1–2.

Zhang J, Qian H. 2023. U.Taxonstand: an R package for standardizing scientific names of plants and animals. *Plant Diversity* 45: 1-5. DOI: [10.1016/j.pld.2022.09.001](https://doi.org/10.1016/j.pld.2022.09.001)

Zizka A, Silvestro D, Andermann T, Azevedo J, Duarte Ritter C, Edler D, et al. 2019. CoordinateCleaner: standardized cleaning of occurrence records from biological collection databases. *Methods in Ecology and Evolution* 7: 744-751.

Zizka A, Antonelli A. 2016. speciesgeocodeR. Version 2.0. [https://CRAN.R-project.org/package=speciesgeocodeR](https://cran.r-project.org/package=speciesgeocodeR)

Zomer RJ, Xu J, Trabucco A. 2022. Version 3 of the Global Aridity Index and Potential Evapotranspiration Database. *Nature Scientific Data* 9: 409.
